# Examining the short-term anxiolytic and antidepressant effect of Floatation-REST

**DOI:** 10.1371/journal.pone.0190292

**Published:** 2018-02-02

**Authors:** Justin S. Feinstein, Sahib S. Khalsa, Hung-wen Yeh, Colleen Wohlrab, W. Kyle Simmons, Murray B. Stein, Martin P. Paulus

**Affiliations:** 1 Laureate Institute for Brain Research, Tulsa, Oklahoma, United States of America; 2 Oxley College of Health Sciences, University of Tulsa, Tulsa, Oklahoma, United States of America; 3 University of California San Diego, La Jolla, California, United States of America; Brown University, UNITED STATES

## Abstract

Floatation-REST (Reduced Environmental Stimulation Therapy) reduces sensory input to the nervous system through the act of floating supine in a pool of water saturated with Epsom salt. The float experience is calibrated so that sensory signals from visual, auditory, olfactory, gustatory, thermal, tactile, vestibular, gravitational and proprioceptive channels are minimized, as is most movement and speech. This open-label study aimed to examine whether Floatation-REST would attenuate symptoms of anxiety, stress, and depression in a clinical sample. Fifty participants were recruited across a spectrum of anxiety and stress-related disorders (posttraumatic stress, generalized anxiety, panic, agoraphobia, and social anxiety), most (n = 46) with comorbid unipolar depression. Measures of self-reported affect were collected immediately before and after a 1-hour float session, with the primary outcome measure being the pre- to post-float change score on the Spielberger State Anxiety Inventory. Irrespective of diagnosis, Floatation-REST substantially reduced state anxiety (estimated Cohen’s d > 2). Moreover, participants reported significant reductions in stress, muscle tension, pain, depression and negative affect, accompanied by a significant improvement in mood characterized by increases in serenity, relaxation, happiness and overall well-being (p < .0001 for all variables). In reference to a group of 30 non-anxious participants, the effects were found to be more robust in the anxious sample and approaching non-anxious levels during the post-float period. Further analysis revealed that the most severely anxious participants reported the largest effects. Overall, the procedure was well-tolerated, with no major safety concerns stemming from this single session. The findings from this initial study need to be replicated in larger controlled trials, but suggest that Floatation-REST may be a promising technique for transiently reducing the suffering in those with anxiety and depression.

**Trial registration:** ClinicalTrials.gov NCT03051074

## Introduction

The history of Floatation-REST dates back to the 1950’s when Drs. Jay Shurley and John Lilly at the National Institute of Mental Health became interested in understanding how the human brain would respond to an environment devoid of external sensory input. It was discovered that rather than falling into a deep sleep or losing consciousness, participants maintained full awareness [1, 2]. Initial designs employed various masks intended to shield the brain from sensory input [1], whereas the first fully immersive floatation tank wasn’t built until 1957, when Dr. Shurley constructed his laboratory at the Oklahoma City Veterans Administration hospital [2]. In this first iteration of Floatation-REST, the participant was immersed vertically in a tank of water with an opaque helmet surrounding their head connected to a series of breathing tubes for ventilation. Due to the confined nature of the helmet, very few individuals participated in these early experiments outside of NASA astronauts in training for the mission to the moon [3, 4].

In the 1970’s, Glenn Perry (in collaboration with John Lilly) invented a horizontal version of the float tank that removed the need to wear a helmet [5]. This newer iteration has individuals lay supine in a shallow pool of water saturated with a high concentration of Epsom salt, allowing individuals to effortlessly float on their back, with the eyes, nose, and mouth comfortably hovering above the water surface. While this change in design exposed floating to a much wider audience, many still found the tanks too confining and claustrophobic in nature. Consequently, floating went through a long period of dormancy up until this past decade, where the practice has witnessed a rapid rise in popularity, likely bolstered by the creation of more spacious tanks and pools. Hundreds of commercial “float centers” have started to open across North America and Europe, where individuals will pay money to float, with sessions typically ranging between 45–90 minutes in duration.

Despite this recent surge in float centers, there has been very little research investigating Floatation-REST. The majority of past floatation research occurred in the 1980’s and 1990’s, primarily in small samples of healthy participants. The most consistent observation to date has been significant reductions in levels of subjective stress and increases in relaxation as measured from pre- to post-float [6–12]. Concomitant with these subjective findings, floating has also been reported to decrease blood pressure [12–17], heart rate [11, 13], as well as plasma cortisol [17–19]; but see [20]. A meta-analysis of 27 Floatation-REST studies found a large overall effect size for stress reduction [21], with most studies focused exclusively on healthy populations.

Clinical research investigating Floatation-REST, although limited, has reported largely beneficial effects across a range of different stress-related conditions, including: hypertension [14, 16], chronic tension headaches [22, 23], chronic muscle tension pain in the back and neck [24], and stress-related pain with “burnout depression” [7, 25]. Thus far there have only been two published Floatation-REST studies focused on individuals with anxiety [23, 26], both in participants with self-reported symptoms of “generalized anxiety." These studies examined the long-term effects of repeated float sessions, but did not assess the short-term effects arising from a single float session. The first study [23] was an uncontrolled investigation at a hospital that collected retrospective surveys in patients who utilized their Floatation-REST facility as part of a stress management program. They found that after ~7 float sessions, patients with generalized anxiety reported improvement in symptoms when assessed an average of 7 months later. The second study [26], a pilot trial in 50 participants randomized to either a waitlist control group or 12 sessions of Floatation-REST, observed a significant reduction in symptoms of generalized anxiety in the float group that was maintained at 6-month follow-up. No studies to date have examined the effects of Floatation-REST in posttraumatic stress disorder (PTSD), panic disorder, agoraphobia, social anxiety disorder, or major depressive disorder, and with the exception of a few individual case studies [27, 28], there has been no other research investigating the impact of Floatation-REST in patients with clinically diagnosed anxiety, depression, or any other mental health disorder.

Anxiety and depression are the two most common mental health disorders, with the proportion of people who will develop a disorder at some point in life (i.e., lifetime morbid risk) estimated to be 42% for anxiety disorders and 30% for major depression [29]. While viewed as separate conditions, comorbidity is often the rule rather than exception, with well over 50% of cases displaying a mix of both anxiety and depression [30, 31]. The cost and toll to society is tremendous, with depression now considered to be the leading cause of worldwide disability [32, 33], and anxiety the sixth leading cause of worldwide disability [34]. The age of onset is typically in adolescence and young adulthood, with symptoms often persisting throughout life without treatment [29, 35, 36]. Pharmacotherapy (e.g., selective serotonin reuptake inhibitor) and psychotherapy (e.g., cognitive behavioral therapy) are the two most commonly prescribed treatments for both anxiety and depression [37, 38]. Recent meta-analyses and large-scale clinical trials suggest that approximately 50% of patients improve with treatment [39, 40], with substantially poorer outcomes and adherence in patients with comorbid anxiety and depression [41, 42]. Given the insufficient treatment response and adherence to currently available therapies, it is important to explore novel ways of helping patients with anxiety and depression.

While the extant research (reviewed above) suggests that Floatation-REST may be a useful technique for stress reduction, there has been essentially no research exploring whether its stress-reducing properties can be effectively applied to individuals with clinically-diagnosed anxiety and depression. Consequently, this study took a transdiagnostic approach by recruiting a heterogeneous sample spanning the spectrum of different anxiety and stress-related disorders including PTSD, Generalized Anxiety Disorder, Panic Disorder, Agoraphobia, and Social Anxiety Disorder, as well as comorbid unipolar Major Depression. Since this was the first Floatation-REST study to examine these different clinical disorders, close attention was paid to issues of safety and tolerability. We were also interested in characterizing the range of different emotional and subjective changes that might arise in this environment, to detect whether any clinically meaningful changes were being induced. We hypothesized that a single session of Floatation-REST would lead to an acute reduction in symptoms of anxiety and depression irrespective of diagnosis.

## Methods

### Ethics statement

All study procedures were approved by the Western Institutional Review Board (WIRB), and all participants provided written informed consent prior to participation. During the consent process, all participants were queried about their understanding of the protocol to ensure they had the capacity to consent and understood that testing was completely voluntarily and could be stopped at any time. The trial was registered on ClinicalTrials.gov (https://clinicaltrials.gov/show/NCT03051074), and this open-label study represents the first float session in the registered trial. Since there were multiple sessions in the registered trial, subsequent revisions have been made to the protocol, but all details for this open-label study were submitted within 21 days of enrollment of the first participant. The protocol for this trial, supporting TREND checklist, and raw data are available as supporting information; see S1 Protocol, S1 Checklist, and S1 Data.

### Participant recruitment

**Fig 1:**
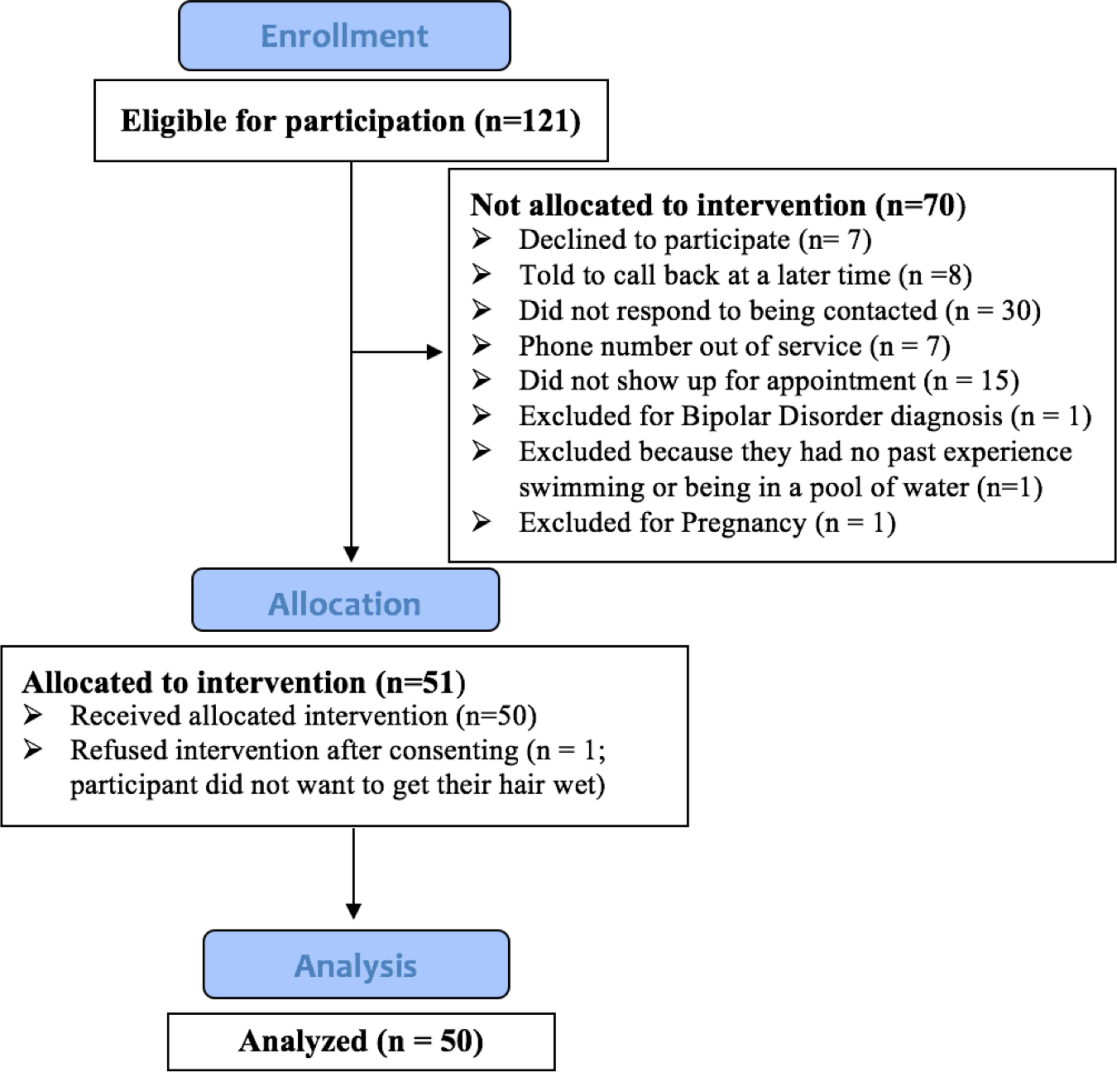
Flow diagram for study recruitment.

Participants for this study were recruited through the Tulsa 1000 (T1000) database maintained at the Laureate Institute for Brain Research (LIBR). The T1000 is a naturalistic study that aims to recruit and longitudinally follow 1000 treatment-seeking individuals from the local community, many of whom have anxiety and/or depression. T1000 subjects were recruited from mental health providers or through general advertisements, and were excluded if diagnosed with any of the following disorders: Schizophrenia Spectrum and Other Psychotic Disorders, Bipolar and Related Disorders, Obsessive-Compulsive and Related Disorders. Each participant received the Mini-International Neuropsychiatric Interview (MINI) version 6.0 [43], a validated, structured, diagnostic interview with questions that parallel symptoms in the Diagnostic and Statistical Manual of Mental Disorders (DSM-IV-TR)[44]. The MINI was administered by interviewers trained in the assessment of anxiety and depression, and all psychiatric diagnoses were confirmed via an extensive review of the clinical history by a board-certified psychiatrist. Based on our inclusion and exclusion criteria (see below), 121 participants from the T1000 were eligible at the time of study recruitment (Fig 1).

#### Inclusion and exclusion criteria

The specific inclusion and exclusion criteria (Table 1) for recruiting participants from the T1000 database were selected based on several considerations. First, since our hypothesis was focused on testing the reduction of self-reported anxiety and depression, we targeted participants across the spectrum of different anxiety and stress-related disorders, many with comorbid unipolar depression. Second, since we were recruiting from a convenience sample assessed at variable intervals in relationship to the onset of this study, we aimed to maximize our chances of selecting individuals who would still present with high levels of anxiety on the day of Floatation-REST. To accomplish this goal, we focused on recruiting participants with very high levels of anxiety sensitivity defined as an Anxiety Sensitivity Index (ASI-3) total score ≥ 30. Anxiety sensitivity refers to an individual’s fear of experiencing anxiety-related symptoms and is a core construct underlying the initiation and maintenance of pathological anxiety [45]. Importantly, individuals with high levels of anxiety sensitivity have a propensity for greater chronicity of illness and a higher likelihood of experiencing future anxiety symptoms [46–48]. We also included a cutoff score of 8 or greater on the Overall Anxiety Severity and Impairment Scale (OASIS), which has been shown to correctly classify 87% of individuals as having a current anxiety disorder diagnosis [49]. Both the ASI-3 and OASIS were re-administered the day of Floatation-REST, and the updated scores are presented in Table 2. Third, since the T1000 is a naturalistic study based on a community sample, we allowed participants who were stably medicated into the study. However, we added exclusion criteria for more severe forms of psychopathology and substance use in order to minimize potential safety risks. And lastly, in order to mitigate effects related to differential expectations and experience, we recruited participants who had never tried Floatation-REST before, but had at least some life experience either swimming or being in a pool of water.

**Table 1:** Inclusion and exclusion criteria.

Inclusion criteria	Exclusion Criteria
1. DSM-IV diagnosis on the MINI of an Anxiety Disorder (Generalized Anxiety Disorder, Social Anxiety Disorder, Panic Disorder, Agoraphobia) and/or Posttraumatic Stress Disorder (PTSD) 2. Overall Anxiety Severity and Impairment Scale (OASIS) score ≥ 8 3. Anxiety Sensitivity Index (ASI-3) total score ≥ 30 4. If taking medication, must be stably medicated prior to participation (defined as having taken the medication for 6 weeks or longer) 5. Between 18–55 years of age 6. No prior Floatation-REST experience	1. Comorbid Bipolar Disorder or Schizophrenia 2. Active suicidality with intent or plan 3. Currently receiving inpatient treatment 4. Current Substance Use Disorder ≥ moderate 5. History of neurological conditions (e.g., epilepsy, stroke, severe traumatic brain injury, Parkinson’s disease, Alzheimer’s disease or other forms of dementia) 6. Any skin conditions or open wounds that could cause pain when exposed to saltwater 7. Inability to swim or lay comfortably in a shallow pool of water

**Table 2:** Participant demographics and baseline functioning on the day of Floatation-REST.

	Anxious Group	Severely Anxious Subgroup	Healthy Reference Sample
**Number of subjects**	50	17	30
**Sex (male/female)**	16/34	5/12	12/18
**Age (years)**	36.8 _(10.9)_	32.3 _(11.2)_	32.5 _(10.4)_
**Education (years)**	14.1 _(2.2)_	14.0 _(2.1)_	14.4 _(2.0)_
**BMI (kg/m**^**2**^**)**	29.3 _(5.2)_	29.6 _(5.7)_	26.3 _(5.8)_
**Anxiety sensitivity (ASI-3)**	26.6 _(14.8)_	43.0 _(9.0)_	7.9 _(7.0)_
**Anxiety severity (OASIS)**	9.6 _(4.2)_	12.8 _(3.3)_	*
**Depression severity (PHQ-9)**	11.4 _(6.0)_	16.3 _(4.1)_	*
**Level of disability (SDS)**	13.5 _(7.8)_	20.2 _(5.7)_	*
**Level of stress (PSS)**	25.7 _(5.9)_	29.8 _(5.5)_	*
**Average life happiness (HM)**	4.4 _(2.3)_	3.2 _(2.4)_	7.7 _(0.5)_
**Net-time happiness (HM)**	-7.3 _(31.2)_	-30.0 _(26.2)_	58.9 _(18.8)_

Numbers in parentheses represent the standard deviation. ASI-3: Anxiety Sensitivity Index total score; OASIS: Overall Anxiety Severity and Impairment Scale; PHQ-9: Patient Health Questionnaire; PSS: Perceived Stress Scale; SDS: Sheehan Disability Scale; HM: Happiness Measure. The severely anxious subgroup is a subset of the participants in the anxious group who had an ASI-3 total score ≥ 30 and an OASIS score ≥ 8 on the day of Floatation-REST.

*Not collected in the Healthy Reference Sample.

#### Reference sample of non-anxious participants

As a point of reference for the float-related findings in anxious individuals, we provide results collected in a sample of 30 healthy non-anxious individuals who were tested in a separate study that involved functional neuroimaging. These individuals were screened to be free of any current or past psychiatric illness (as determined by the MINI) and were not taking any drugs or medications. Although there were several aspects to this other study that differed from the present study (which we describe below), there were also a number of similarities: (1) all 30 non-anxious participants had no prior Floatation-REST experience, (2) the data we show is derived from their first float session, which took place in the same open float pool, and (3) we collected many of the same self-report measures both before and after their float session. There were no brain scans the day of their first float session, and the experimental procedures were largely identical to the present study with the following exceptions: (a) float sessions were 90 minutes in length and participants were encouraged to float for the entire 90 minutes (whereas the anxious participants were instructed that they could float for “up to 60 minutes” and could end the float at any time), and (b) participants were encouraged to keep the lights off for the duration of their float session (whereas the anxious participants were given the choice to keep the lights on or off). Due to these differences, we did not perform any statistical comparisons between the anxious and non-anxious samples. Therefore, the results from this group of non-anxious participants are presented in this paper merely as a point of reference to provide context for the findings observed in the anxious sample.

### Measures

All self-report measurements were administered electronically to participants via an Apple iPad using REDCap (Research Electronic Data Capture), a secure web-based application for the electronic collection and management of research and clinical trial data (www.project-redcap.org). REDCap servers are housed in a local secure data center at LIBR with all web-based transmission encrypted and all participant data de-identified using a unique identifier code to ensure patient confidentiality. Three different types of self-report measures were administered: baseline measures, pre/post-float measures, and follow-up questions. The baseline measures were focused on assessing current symptomatology and overall mood and level of functioning during the time period of the Floatation-REST study. The pre/post-float measures were collected at two time points, approximately 30 minutes before and after each float session, in order to assess for fluctuations in state affect and mood. At each time point, participants rated how they felt “right now, in the present moment”. Along these lines, when completing the post-float measures, participants were explicitly instructed not to retrospectively rate how they felt during the float itself. In contrast, the follow-up questions were primarily aimed at gathering additional information about each participant’s actual float experience, including an assessment of potential adverse events.

#### Baseline measures

**Anxiety Sensitivity Index (ASI-3):** The ASI-3 is an 18-item questionnaire that has been shown to have good reliability and validity, and improved psychometric properties over the original measures [48]. Questions are answered using a 4-point scale and total ASI scores can range from 0 to 72. Normative data collected in a large sample (n = 4,720) of healthy North Americans indicate a mean ASI-3 total score of 12.8 (SD = 10.6) [48]. A meta-analysis [46] found that patient groups with anxiety and depression commonly have a total ASI score above 30, and other studies have used a cutoff score ≥ 30 to recruit individuals with very high levels of anxiety sensitivity [50, 51].

**Overall Anxiety Severity and Impairment Scale (OASIS):** The OASIS is a 5-item questionnaire that can be used across the different anxiety disorders as a continuous measure of anxiety severity and impairment over the past week [52]. Each item is rated on a 5-point scale and the ratings are summed to obtain a total score ranging from 0 to 20. A cut-score of 8 has been shown to correctly classify 87% of individuals as having a current anxiety diagnosis [49].

**Patient Health Questionnaire (PHQ-9):** The PHQ-9 is a 9-item measure for assessing the severity of depressive symptoms over the past 2 weeks [53]. Scores of 1–4 are considered indicative of minimal depression, 5–9 mild depression, 10–14 moderate depression, 15–19 moderately severe depression, and 20–27 severe depression.

**Perceived Stress Scale (PSS):** The PSS is a 10-item questionnaire used for measuring an individual’s perception of stress in their life over the past month [54, 55]. The PSS has good psychometric properties [56], with mean normative scores in healthy populations around 13 (SD = 6) [55].

**Sheehan Disability Scale (SDS):** The SDS assesses how much the respondent’s mental health issues are perceived to have affected their daily activities in three functional domains: work/school, social/leisure activities, and family life/home responsibilities [57]. Total disability scores range between 0 to 30, with scores ≥ 5 signifying impairment [58]. A review of studies using this measure indicated significant impairment in functioning in patients with anxiety disorders, who have mean total disability scores typically ranging between 14–18 [59].

**Happiness Measure (HM):** The HM is a short trait measure of emotional well-being [60] comprised of 4 questions assessing how happy or unhappy an individual generally feels. The first question asks the person to choose a statement that best describes their average happiness on a scale from 0 (Extremely unhappy) to 10 (Extremely happy). The remaining questions asks the individual to rate the average percentage of time they feel *happy*, *unhappy*, and *neutral* with percentages totaling to 100%. A net-time happiness score is calculated by subtracting the % time unhappy from the % time happy. The HM has been reviewed in relation to other well-being measurements and has been shown to be a quick and efficient measure with good reliability and discriminative validity [60].

#### Pre/Post-float measures

**Primary outcome measure—State-Trait Anxiety Inventory (STAI-Y State form):** The Spielberger State Anxiety Inventory is a widely used 20-item self-report questionnaire designed to assess an individual’s level of anxiety at the present moment with total scores ranging from 20–80 [61]. The items assess for the presence or absence of current anxiety symptoms, and the measure has been shown to have excellent internal consistency and good convergent and discriminant validity [61]. Many other clinical trials have used this measure, including pharmacological (e.g., [62, 63]) and non-pharmacological (e.g., [64, 65]) interventions for anxiety.

**Positive and Negative Affect Schedule—Expanded Form (PANAS-X):** The PANAS is one of the most commonly used measures of mood, comprised of two 10-item scales used to extract measures of positive and negative affect [66]. 13 additional items were added from the PANAS-X [67] to compute the specific affect scales of Serenity, Joviality (referred to here as “Happiness”), and Fatigue. The PANAS-X has high internal consistency, and good convergent, discriminant, and construct validity [66, 67]. Participants completed this version of the PANAS-X using the “at the present moment” instructions.

**Karolinska Sleepiness Scale (KSS):** The KSS is a single item measure of present moment sleepiness that has been validated against relevant behavioral and electroencephalography measures [68, 69].

**Wong-Baker Pain scale:** This commonly used pain measure has participants rate their current level of pain from 0 to 10 using drawings of faces that range from smiling to crying [70].

**Visual Analogue Scales (VAS):** Each VAS measure contained a digital slider that participants could move along a horizontal line. Seven VAS measures contained a slider that started on the far left and had participants rate how they currently felt on a 100-point scale that went from “Not at all/None” (far left) to “Extremely/The most I have ever felt” (far right). These VAS measures were used to capture potential changes with regard to subjective: Relaxation (How relaxed do you feel right now?), Muscle tension (How much muscle tension or tightness do you feel right now?), Stress (How stressed or anxious do you feel right now?), Depression (How sad, down, or depressed do you feel right now?), Content/Peaceful (How content or peaceful do you feel right now?), Refreshed (How refreshed do you feel right now?), and Energy (How much energy do you have right now?).

In addition, we also included a modified VAS that assesses Overall Well-Being using a bipolar valence scale that goes from “Pretty Bad” (far left) to “Pretty Good” (far right), with the slider starting in the middle at “Neutral” and asking, “In general, over the past hour, how have you felt?”

#### Follow-up questions

After completing the post-float measures, participants answered some additional follow-up questions. One question asked, “At the end of your float, how did you feel about the duration?” and contained three answer choices: (1) I wanted to get out before my time was up, (2) It was the perfect amount of time, (3) I wish I could have stayed in longer. Another question asked, “What other techniques have you tried to help you relax and feel less anxious and stressed?” Participants were provided with 12 different options and were instructed to check all techniques that they have tried at least once: anti-anxiety medication, psychotherapy/counseling, massage, exercise, alcohol, breathing techniques, cigarettes, marijuana, progressive muscle relaxation, meditation, yoga, and other. The next question asked, “How did the relaxation you experienced during and after today’s float session compare to the other relaxation techniques you have tried?” and contained three answer choices: (1) I experienced more relaxation with other techniques, (2) Floating was equally as good as the other techniques I have tried, (3) I experienced more relaxation with floating than any other technique I have tried. Two final questions asked, “Would you be interested in floating again sometime in the future?” and “Do you think floating in these specialized pools has the potential to be an effective therapy for reducing stress and anxiety and improving people's mood?”, with each question containing three answer choices: Yes, No, Maybe.

#### Side effect checklist

After completing the follow-up questions, participants completed a 43-item side effect checklist that was created for this study in order to assess the safety of Floatation-REST in this clinical population, and probe for the presence of potential adverse experiences. The instructions asked, “Did you notice or experience an **increase** in any of these items during or shortly after your float today? Please only mark items that showed an increase from your typical day-to-day experience.” For each item, participants had to select one of four choices (None, Mild, Moderate, or Extreme) and for any response other than “None” a free-response box allowed them to describe their experience in more detail. Two-thirds of the items described a range of different negative experiences, many probing various psychiatric symptoms including panic, dissociation, flashbacks, suicidality, mania, psychosis, and negative thought content. In order to not bias the participant, the other one-third of items described a range of different positive experiences, including items probing for the presence of peak life experiences.

#### Debriefing interview

In order to further assess for the occurrence of adverse events, as well as gather more qualitative information about the float experience, each participant completed a short debriefing interview with the experimenter at the end of each visit using a series of open-ended questions: Overall, how was your float today? What did you think about, if anything, while you were floating? Did anything surprise you during the float or happen unexpectedly? Did you learn anything about yourself during this experience? All responses were recorded with a digital audio recorder and later transcribed (see S1 Debriefing interview transcriptions).

### Floatation-REST intervention

**Fig 2:**
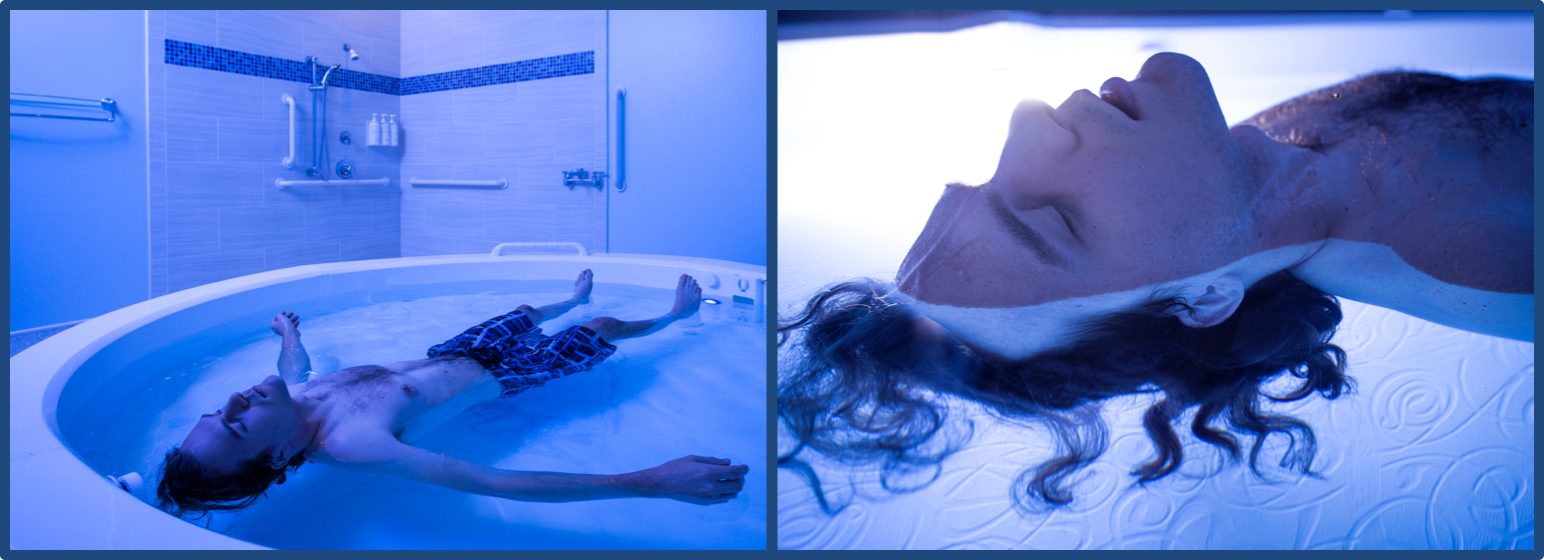
Floatation-REST in an open circular float pool. The circular fiberglass pool is 8 feet in diameter and contains 11 inches of reverse osmosis water saturated with ~1,800 pounds of USP grade Epsom salt (magnesium sulfate), creating a dense salt water solution that is maintained at a specific gravity of ~1.26, allowing participants to effortlessly float on their back while the water hovers just above the ears. A small blue LED light remains illuminated throughout the float session, and can be turned off by the participant through a round air switch (both of which can be seen in the picture, located immediately adjacent to the participant’s right foot). Unlike the picture, clothing is usually not worn while floating since anything touching the body can generate somatosensory stimulation, potentially detracting from the float experience.

The Float Clinic & Research Center at the Laureate Institute for Brain Research contains an open float pool (Fig 2) that was custom-designed for research purposes by Floataway (Norfolk, United Kingdom). The open float pool was designed to help reduce the barriers to entry for anxious populations, including: (1) elimination of the enclosure commonly found on most float tanks in order to reduce feelings of claustrophobia, (2) use of a high-powered disinfection system for sterilization of the water, and (3) providing the participant with complete control over the experience so that they can freely enter and exit the pool at any time, as well as have the lights on or off at their choosing.

Since the open pool has no enclosure, the room around the pool was constructed to be waterproof, soundproof, lightproof, and temperature-controlled in order to provide a similar experience to fully-enclosed float tanks. Silent heaters were placed under the pool to maintain the water at a constant temperature and a dedicated heating, ventilation, and air conditioning system maintains the air at a constant temperature. The temperature of the water and air was calibrated to approximate the surface temperature of the skin (~95.0 °F), and could be adjusted remotely by the experimenter in a nearby control room. An intercom system allowed the participant to freely communicate with the experimenter throughout the float session should any issues arise, and specialized speakers placed around the perimeter of the pool allowed the experimenter to communicate with the participant and play music. There are no cameras inside the float room and the outside door was locked to ensure complete privacy throughout the float session.

While floating, a blue LED light remains illuminated in the background and could be turned off by the participant using an air switch. Once triggered, the air switch activates an infrared wave detection system so that participants can remain floating and still turn the blue light on and off simply by waving their arm in the air. In addition, a small sensor placed slightly above the water line discerns when the float pool is occupied. Both the occupy sensor and wave detection system are linked to digital clocks in the control room, allowing for the automated calculation of the total amount of time that a participant is floating and the total amount of time that a participant is floating with the lights off.

The float experience is calibrated so that sensory signals from visual, auditory, olfactory, gustatory, thermal, tactile, vestibular, gravitational and proprioceptive channels are minimized, as is most movement and speech. Visual stimulation is minimized by having an entry door and gasket system which expunges all sources of outside light. In addition, there are no windows inside the float room, and the adjacent room contains a private bathroom that also has no windows, and no lights (which are automatically shut off during the float itself). Thus, when the entry door to the float room is sealed and the blue LED light inside the pool is turned off, the float room is completely dark. Auditory stimulation is minimized by constructing the float room using multiple layers of sound dampening walls with thick insulation and added soundproofing material, restricting most outside airborne sound from entering the room. Structural sounds transmitted via vibrations in the floor are absorbed by having the pool rest on a bed of 48 butyl rubber springs, effectively isolating the pool from the building and preventing all structure-borne noises from entering the water. Olfactory stimulation is minimized by only using unscented cleaning products and having the participant shower beforehand to help remove body odors. In addition, the water disinfection system (described in more detail below) does not emit any odors during the oxidative process. Gustatory stimulation is minimized by having participants eat several hours before the float, while refraining from eating and drinking during the float. Thermal stimulation is minimized by setting the temperature of the water and the air to closely match the temperature at the surface of the skin, which is typically a few degrees cooler than core body temperature. All temperature sensors were calibrated using a Thermoworks precision thermometer (Utah, USA) certified by the National Institute of Standards and Technology (NIST). Throughout each float session, the water temperature was maintained at 95.0 °F (±0.3 °F) and the air temperature at the rim of the pool was maintained at 93.5 °F (±0.5 °F), slightly lower than the water temperature based on the relative humidity in the air. This temperature setting helps minimize the need for thermoregulation while reducing the perceptual boundary between air, body, and water, a unique feature of the float experience. Specific gravity of the water was measured using an H-B Instrument Polycarbonate Hydrometer (Pennsylvania, USA), with a specific gravity range of 1.20–1.42 and NIST calibrated to achieve an accuracy within 0.002. The density of the water and salt concentration was maintained at a specific gravity between 1.25–1.26 for all float sessions. Consequently, stimulation from tactile, vestibular, gravitational, and proprioceptive channels is minimized by the body’s immersion in the dense saline solution, buffering the body against the forces of gravity and allowing the individual to effortlessly float on their back in a state of stillness. The importance of “stillness” is also emphasized during the pre-float instruction set (S1 Floatation-REST procedure), further helping to reduce both movement and speech.

#### Water disinfection

Beyond the natural sanitation created by the high concentration of salt, the water inside the float pool is reverse osmosis water that is circulated through a powerful disinfection system which includes surface skimming, 10-micron filtration, 6 ultraviolet (UV) lights, and 35% Hydrogen Peroxide (H_2_O_2_) maintained at ~50ppm. The unique combination of UV with H_2_O_2_ generates a free hydroxyl radical that is nearly twice as powerful as chlorine at oxidizing and destroying microorganisms [71], while also ensuring that no dangerous byproducts or scented odors are emitted into the air during the oxidative process (a major advantage over other disinfection systems which use chlorine, bromine, or ozone). Between every participant, the water is continuously circulated through the disinfection system, with a minimum of 4 turnovers.

#### Procedures

Participants were instructed that they could float for “up to 60 minutes” and could end the float at any time. Since this was the first Floatation-REST study investigating participants with moderate to severe anxiety and depression across a spectrum of different clinical diagnoses, several extra precautions were taken to ensure the safety of every participant. All procedures were administered by individuals with training in mental health and medical care (including a clinical neuropsychologist and a medical doctor). Each participant was provided with a thorough overview of the procedure during the informed consent process and was encouraged to ask questions and express any worries or concerns that they might have. Participants were also informed that they could ask questions or speak to the experimenter at any time during the float through the intercom system. A microphone located inside the float room provided a real-time continuous audio feed to a nearby control room, where the experimenter remained throughout the float session so that they could quickly address any issues that may arise. In addition, a debriefing interview took place after every float session so that the experimenter could assess whether or not any adverse events had taken place, and address them accordingly prior to the participant leaving. Additional procedures were created to help standardize testing across participants (see S1 Floatation-REST procedure).

### Statistical analysis

Sixteen different measures were collected before and after each float session. The score for each measure was converted into a POMP score, standardized units representing the “Percent Of Maximum Possible” for each measure, ranging from 0–100% [72]. Pre- to post-float change scores were computed for each measure and the effect size (Cohen’s d) was estimated by dividing the group’s mean change score with the standard deviation of changes [73]. A 95% confidence interval for each effect size was estimated by percentile approach using 10,000 bootstraps. Within the Anxious Group, the pre-post changes were compared by linear mixed-effects models (LMM) using Time (Post vs. Pre), age, gender and medication status as fixed-effects, and subject as the random (intercept) effect. It is important to note that this study was neither designed nor powered to test for subgroup differences, and therefore, all subgroup results are presented as estimated effect sizes. One subgroup analysis compared participants who were “severely anxious” on the day of testing (defined as participants who continued to meet our strict inclusion criteria of having an ASI-3 total score ≥ 30 and an OASIS score ≥ 8) with the remainder of the anxious sample. Additional analyses assessed for differential effects based on other variables, including: diagnosis (Generalized Anxiety Disorder, PTSD, Panic Disorder, Agoraphobia, Social Anxiety Disorder), sex, medication status (divided into those currently taking prescribed medication for their anxiety/depression and those who were unmedicated), and level of visual stimulation (based on whether the participant floated with the lights on or off). All analyses were performed on RStudio version 1.0.136 with R version 3.3.2, using the R packages boot (version 1.3–18) for bootstrap, lme4 (version 1.1–12) for LMM, and lmerTest (version 2.0–33) for Satterthwaite’s degrees of freedom and p-values. False Discovery Rate (FDR) for multiple outcome measures were controlled by Benjamini-Hochberg’s method at a 5% level.

## Results

### Sample characteristics

Fifty participants who met our inclusion and exclusion criteria underwent Floatation-REST. Table 2 provides details about the participant demographics and their baseline level of functioning on the day of Floatation-REST. The sample spanned the spectrum of different anxiety disorders, with many comorbidities, including a mix of Generalized Anxiety Disorder (n = 26), Social Anxiety Disorder (n = 16), Panic Disorder (n = 12), Agoraphobia (n = 8), and Posttraumatic Stress Disorder (n = 17). Nearly every participant (n = 46, or 92% of the sample) also presented with comorbid unipolar Major Depression. Forty percent of the sample (n = 20) was stably medicated on a selective serotonin reuptake inhibitor (SSRI) or serotonin–norepinephrine reuptake inhibitor (SNRI), and 8 participants were also taking a benzodiazepine (of note, none of the participants reported taking a benzodiazepine the day of their float session). On the day of Floatation-REST, most participants were acutely anxious (average OASIS score = 9.6) and depressed (average PHQ-9 score = 11.4), with average scores in the clinical range of severity. Participants also presented with high levels of perceived life stress (average PSS score = 25.7) and anxiety sensitivity (average ASI-3 total score = 26.6), as well as marked impairment in social and occupational functioning (average total disability score on the SDS = 13.5). Consistent with their high level of distress and impairment, participants reported spending most of their time in a state of unhappiness (average net-time happiness = -7.3). The severely anxious subgroup, comprised of the participants who continued to meet our strict inclusion criteria of an ASI-3 total score ≥ 30 and an OASIS score ≥ 8 when measured the day of Floatation-REST, also reported more severe symptoms (of both anxiety and depression), along with greater levels of distress and disability.

### Safety and tolerability of intervention

There were no serious adverse events or major safety concerns arising during this initial float session. Most participants (48/50) chose to float for the entire hour (S1 Fig). Only 2 participants ended their float session early, with one participant exiting 48 minutes into their float, and the other exiting after 22 minutes. The latter participant informed us during debriefing that she had exited early due to the salt water causing a stinging sensation on her back where she apparently had several small cuts that she was unaware of beforehand. Roughly half of the participants (n = 24) chose to float with the blue light on for most of the session (average time in dark = 2.1 minutes), whereas the other half of participants (n = 26) chose to float with the blue light off for most of the session (average time in dark = 53.4 minutes)(S1 Fig). At the end of the float, when participants were queried about the 60 minute float duration, 24 participants (48%) said they wanted to stay in longer, 15 participants (30%) said it was the perfect amount of time, and 11 participants (22%) said they were ready to get out before the hour had elapsed.

**Fig 3:**
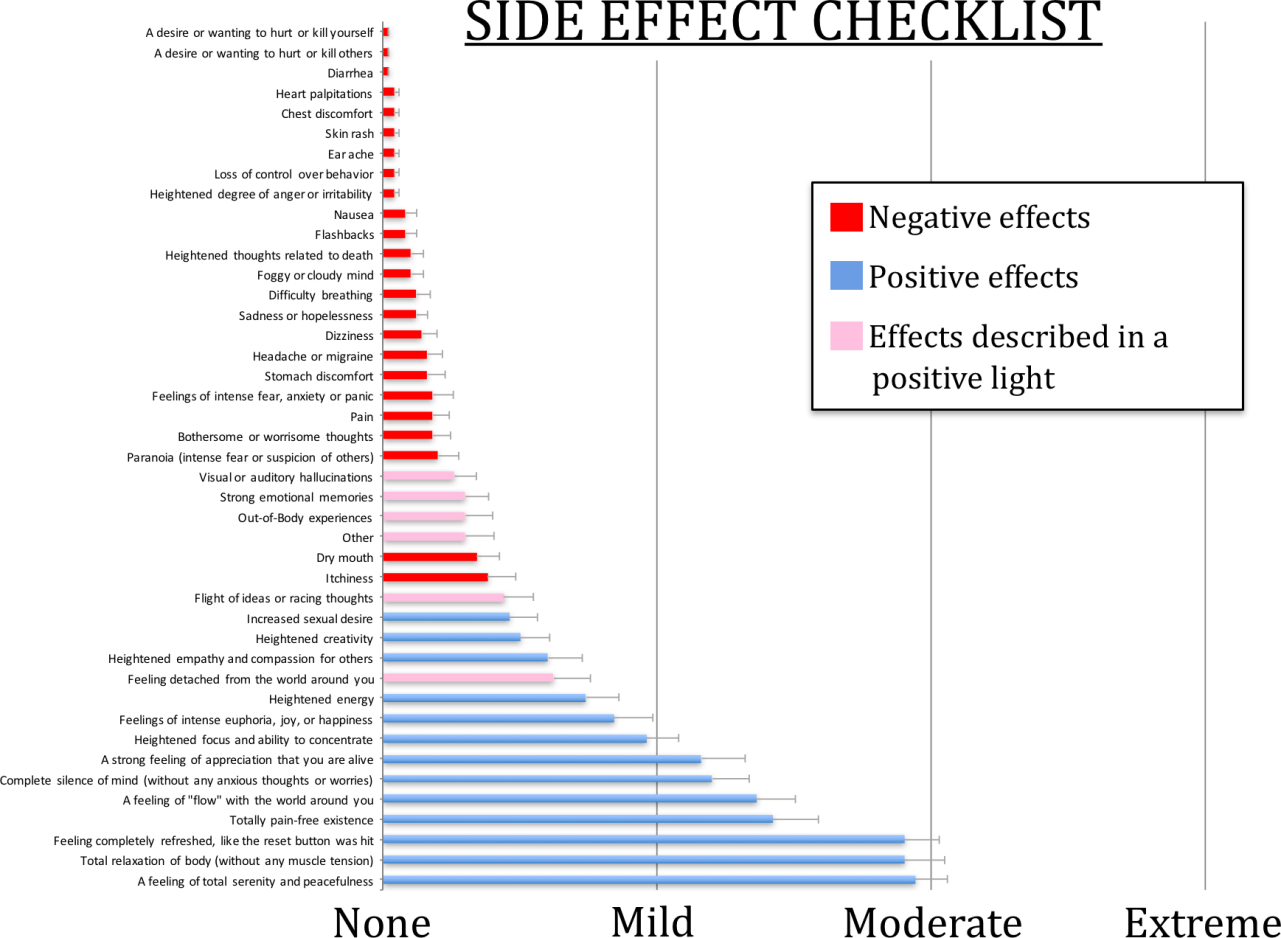
Side effect checklist. After the float session, participants completed a 43-item side effect checklist. For each item participants selected one of four choices (None, Mild, Moderate, or Extreme) and each choice was automatically scored as a number (0, 1, 2, or 3). Shown here is the average score across the group of 50 anxious and depressed participants, with error bars representing the standard error of the mean (SEM).

On the side effect checklist (Fig 3) there were very few instances of negative side effects, with the two most prominent being occasional reports of mild itchiness and dry mouth. Two-thirds of the items described a range of different negative experiences, and the other one-third of items probed a range of different positive experiences. For any response on the checklist other than “None”, a free-response box allowed participants to describe their experience in more detail. Items in pink (Fig 3) refer to potentially negative experiences that were described by participants in a positive light (e.g., for *Out-of-Body experiences* one participant wrote, “At times I felt completely out of my body in a pleasant way” and for *Feeling detached from the world around you* they wrote, “I felt detached from the world in a good way while floating as if I was in a sanctuary”). Reports of visual or auditory hallucinations were a low base rate event. When hallucinations were reported, they were always nondescript and described in positive terms, with the most common report being visions of various colors and shapes: “*I saw these lines that were moving yellowish*, *gold in color”*, *“Visual lights and color*, *very pretty and relaxing*”, “*It was completely dark*, *but a halo of light was coming up from the pool*”, “*I saw little flashes of light like starbursts randomly in different places*. *It was beautiful*.”

One subject reported a notable adverse experience comprised of extreme feelings of intense anxiety and paranoia (“*For the first twenty or so minutes*, *I had severe anxiety*. *The next 20 were spent calming myself down and trying to enjoy the float*”) accompanied by mild difficulty breathing and heart palpitations, as well as bothersome thoughts (“*For the first half*, *I felt irrationally frightened and in danger*”). During debriefing, she reported that she had experienced a panic attack which started at the very beginning of her float, and proceeded to heighten in intensity during the first 20 minutes. The feeling, however, was not strong enough to cause her to exit the pool, and by the end of her float session she had fully recovered and reported a marked absence of fear, anxiety, or panic, that was replaced by “extreme” positive feelings on the side effect checklist including total serenity and peacefulness, total relaxation of body, and complete silence of mind (without any anxious thoughts or worries).

Far more common were reports of positive experiences, with many participants endorsing positive effects in the “moderate” to “extreme” range of intensity. Despite there being twice as many negative options as positive options listed on the checklist, reports of positive experiences overshadowed all of the negative experiences. The top 10 rated effects were all positive (Fig 3), with the top 3 being “A feeling of total serenity and peacefulness”, “Total relaxation of body (without any muscle tension)”, and “Feeling completely refreshed, like the reset button was hit.” The preponderance of positive experiences was further highlighted in the debriefing interviews, where many participants reported that the float experience had a very powerful effect (see S1 Debriefing Interview Transcriptions).

### Impact of Floatation-REST on clinically relevant symptoms

**Fig 4:**
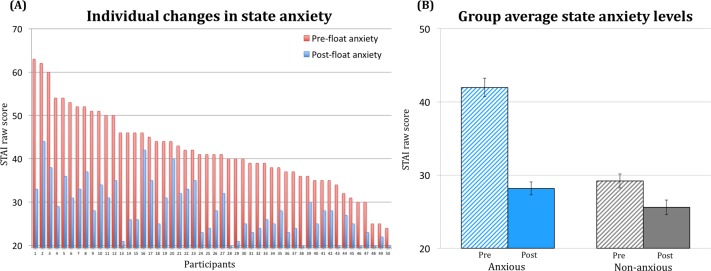
Impact of Floatation-REST on state anxiety. (A) The float experience caused a reduction in state anxiety that was evident across all 50 participants, leading to a significant pre- to post-float change on the Spielberger State Anxiety Inventory (STAI) at the group level [t(49) = -15.16, p < .0001, d = 2.15]. (B) Despite a large baseline difference, the anxious group’s average post-float anxiety had reached levels slightly lower than the pre-float anxiety reported by the non-anxious reference sample. Error bars represent the SEM.

**Fig 5:**
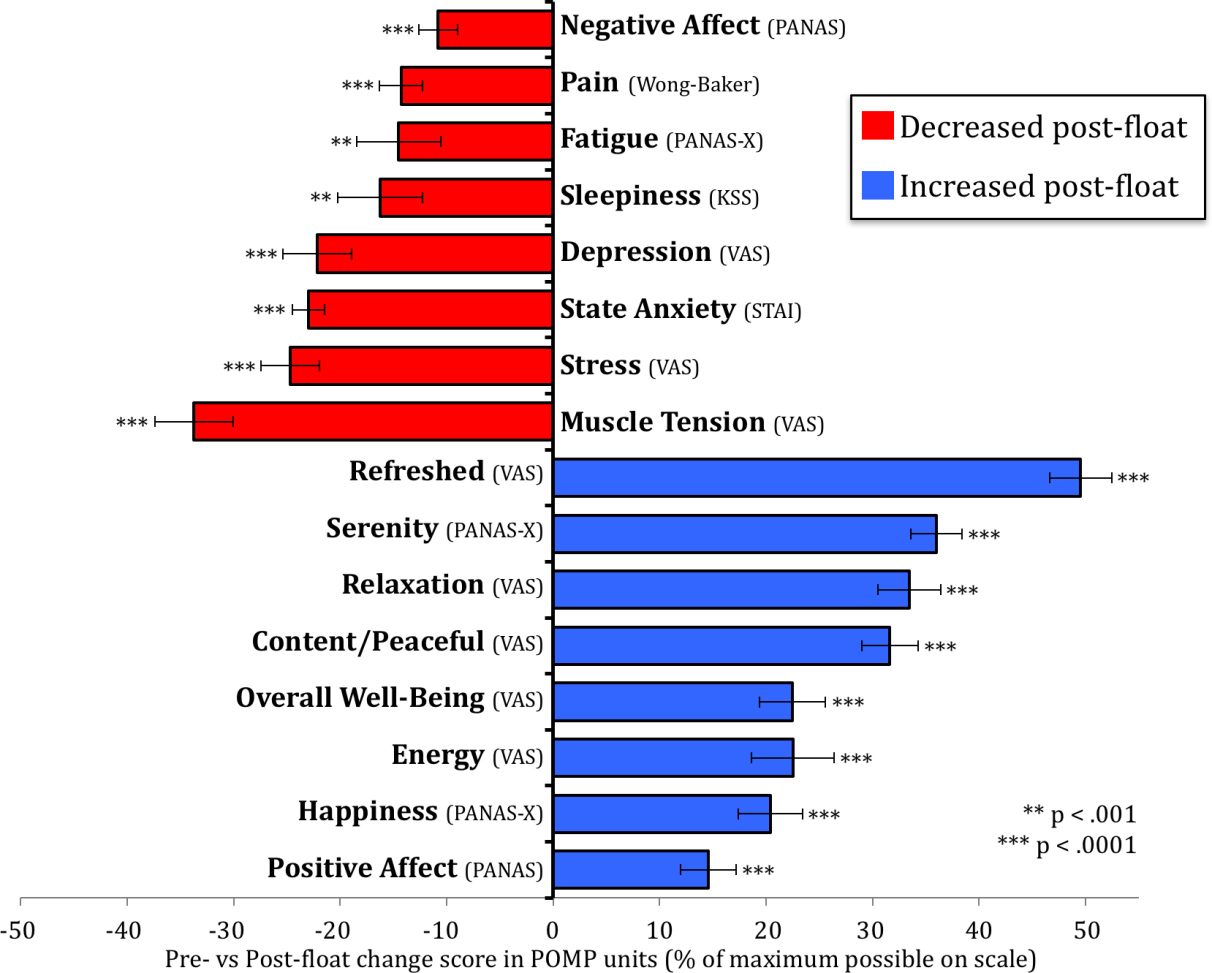
Impact of Floatation-REST on mood and affect. Change scores from pre- to post-float are shown for all 16 measures. To facilitate comparisons across measures the score for each measure was converted to POMP units representing the percent of maximum possible on each scale. All measures showed a significant pre- to post-float change with the significance level denoted with asterisks. Error bars represent the SEM.

**Fig 6:**
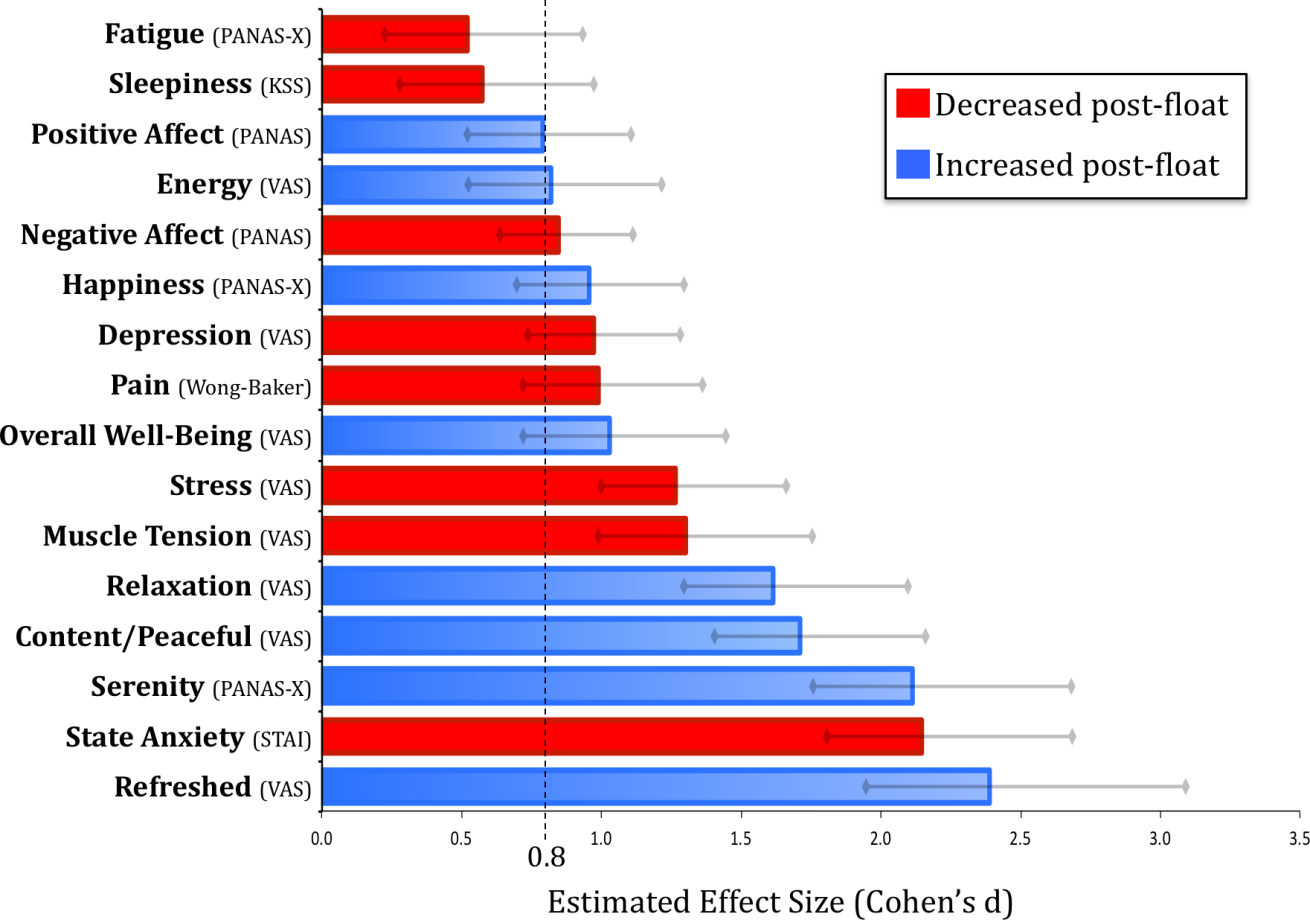
Estimated effect size of a single float session in patients with anxiety and depression. The estimated Cohen’s d is shown for each pre- to post-float change score, with grey lines representing the 95% confidence interval. The dashed black line demarcates the starting point (d = 0.8) for what is considered a “large effect size” [73].

On our primary outcome measure, the float experience induced a reduction in self-reported state anxiety that was evident across all 50 participants (Fig 4A). Moreover, when compared to the non-anxious reference sample, the anxious group’s post-float state anxiety approached non-anxious levels (Fig 4B). Across the different pre/post-float measures there were a number of significant effects found in the anxious sample (Fig 5). Significant reductions were observed in state anxiety, stress, muscle tension, pain, depression, and negative affect (all changes were significant at p < .0001). There was also a substantial improvement in mood characterized by increases in serenity, relaxation, happiness, positive affect, overall well-being, energy levels, and feeling refreshed, content and peaceful (all changes were significant at p < .0001). In reference to the non-anxious sample (comprised of participants who were also naïve to floating), the anxious group reported experiencing float-induced changes that were considerably larger in magnitude (S2 Fig). Effect size estimates for each of the variables in the anxious sample revealed that most effect sizes ranged from large to very large (Fig 6). The largest effects were reductions in state anxiety (d = 2.15) and increases in serenity (d = 2.11) and feeling refreshed (d = 2.39).

#### Exploratory analyses

Subgroup analyses revealed similarly large effect sizes irrespective of diagnosis, sex, medication status, and level of visual stimulation during the float (S3 Fig). There were only a few modest differences between conditions or diagnostic categories. For example, individuals who floated with the lights on (vs off) tended to experience smaller effects on several measures including serenity (d = 1.79 vs 2.51), overall well-being (d = .78 vs 1.39), stress (d = -.95 vs -1.72), and negative affect (d = -.65 vs -1.05), whereas state anxiety reduction showed a similar effect regardless of having the lights on or off (d = -2.19 vs -2.15). In general, measures of fatigue and sleepiness displayed the smallest effects and most variability between subgroups; for example, the Social Anxiety Disorder subgroup had very small effects with regard to fatigue (d = -.17), whereas the PTSD subgroup had large post-float reductions in fatigue (d = -.96). On key symptom-related variables—such as reductions in state anxiety, stress, depression, and negative affect—the effect sizes were consistently large across all subgroups (S3 Fig). Further analysis revealed that the largest effects occurred in the most severely anxious participants (S4 Fig).

On the follow-up questions, all 50 participants requested to float again, and 47 of the participants thought that floating had the potential to be an effective therapy for reducing stress and anxiety and improving mood. Nearly three-quarters of the sample (37 participants) reported that they achieved more relaxation with floating than any other treatment or technique they had tried in the past (S5 Fig).

## Discussion

This study found that a single one-hour session of Floatation-REST was capable of inducing a strong reduction in state anxiety and a substantial improvement in mood in a group of 50 anxious and depressed participants spanning a range of different anxiety and stress-related disorders (including PTSD, Generalized Anxiety Disorder, Panic Disorder, Agoraphobia, and Social Anxiety Disorder). The findings from this open-label study suggest that Floatation-REST may be a promising technique for acutely reducing symptoms of anxiety and depression, although the persistence of these effects is presently unknown. With regard to our primary outcome measure, the reduction in state anxiety was evident in every participant regardless of sex or medication status (Fig 4A). Moreover, the anxiety reduction was robust, with an estimated Cohen’s d > 2 across all disorders and subgroups (S3 Fig). Beyond the immediate dissipation of anxiety, the float experience also induced a significant decrease (p < .0001) in self-reported stress, muscle tension, pain, depression, and negative affect, along with a significant increase (p < .0001) in serenity, relaxation, happiness, positive affect, overall well-being, energy levels, and feeling refreshed, content and peaceful (Fig 5), with estimated effect sizes ranging from large to very large across variables (Fig 6).

On key symptom-related variables—such as reductions in state anxiety, stress, depression, and negative affect—the effect sizes were consistently large across all tested diagnostic categories (S3 Fig). An exploratory subgroup analysis revealed that those with the most severe anxiety reported the largest effects (S4 Fig). This latter finding is notable given the fact that the severely anxious participants reported having the most severe impairments in life functioning (Table 2), and also tended to be the most resistant to other forms of treatment; approximately two-thirds of the severely anxious participants were currently taking an SSRI or SNRI, and over three-quarters had tried psychotherapy. Indeed, most participants in this study reported having tried a number of other techniques to help them relax and feel less anxious and stressed (S5 Fig). Of potential clinical relevance, nearly 75% of the entire sample, and 82% of the severely anxious subgroup, reported that they had achieved more relaxation with Floatation-REST than any of the other treatments or techniques they had tried in the past (S5 Fig). While demand characteristics, expectancy effects, novelty effects, and retrospective recall biases may be inflating these subjective comparisons with other therapeutic modalities, the debriefing interviews (S1 Debriefing interview transcriptions) revealed that the float experience had a powerful positive effect on many of the participants. In future studies, it will be important to assess whether such positive effects can be maintained, or even further improved, with repeated sessions of Floatation-REST.

In comparison to previous float studies [21], the effect sizes observed in our anxious and depressed sample were about twice as large. Since many of the previous studies tested healthy participants, this noted disparity in effects may be driven by the possibility that Floatation-REST provides the largest effect to those who bring the most stress into the float experience. Such an interpretation is consistent with the larger float-induced changes observed in the severely anxious subgroup (S4 Fig), and the relatively small float-induced changes observed in the non-anxious reference sample (S2 Fig). While this study was not designed to directly compare anxious to healthy samples, the limited data we have on this matter suggests that Floatation-REST may temporarily lower state anxiety to near-normal levels (Fig 4B).

Although mood and anxiety disorders are heterogeneous in terms of their diversity of symptoms and emotional triggers, recent efforts have attempted to develop more effective treatments that can work in a transdiagnostic manner [74–76]. Transdiagnostic treatments have the obvious advantage of being easier to disseminate and more widely applicable, especially given the high rate of comorbidity between the different mood and anxiety disorders. To our knowledge, this is the first Floatation-REST trial in individuals across the spectrum of anxiety and depression, with results showing clear signs of short-term benefit in PTSD, Generalized Anxiety Disorder, Panic Disorder, Agoraphobia, Social Anxiety Disorder, and Major Depression. Previous float research with psychiatric populations did not assess the short-term effects following a single float session, but did assess the long-term effects (up to 6 months post-treatment) following 12 float sessions, and found evidence for sustained long-term benefit in individuals with generalized anxiety [26] or burnout depression [7, 25]. Together, these findings suggest that Floatation-REST may have the potential to be a viable transdiagnostic therapy for relieving symptoms of anxiety and depression.

Anxiety and depression affect over a quarter of the population, yet more than three-quarters of patients never receive treatment [35, 77]. This utilization problem is even worse in patients with social phobia [78, 79] or multiple comorbidities [77, 78, 80]. Novel non-pharmacological therapies for anxiety and depression are desperately needed, and the promising results of this initial Floatation-REST trial warrant further investigation, especially given the insufficient response rates and adherence to currently available treatments [39–42], and the paucity of novel medications reaching market over the past two decades [38]. In addition, many medications come with a host of side effects, which further contributes to the poor rates of adherence. The barriers to treatment utilization are complicated, but one of the most common reasons why patients with mood and anxiety disorders fail to receive treatment is due to the notion that they want to solve the problem on their own [81]. In this regard, Floatation-REST may offer an attractive alternative option that enhances self-efficacy and improves treatment utilization by providing anxious patients with the opportunity to learn new ways of coping with distress on their own. In addition, Floatation-REST appeared to be well-tolerated by this sample, with minimal evidence of harm, adverse events or major safety concerns arising during the initial float session. Positive experiences outweighed all negative experiences, and consequently, 96% of the participants chose to float for the entire hour, and 100% of the participants requested to float again. The non-pharmacological nature of Floatation-REST, combined with its lack of side effects, ease of use, and rapid onset of benefit, are additional positive attributes that may further improve treatment utilization and adherence.

### A note on “sensory deprivation”

In contrast to the prevailing positive experience reported post-float, there were clear signs of pre-float anticipatory anxiety and avoidance behavior. Fifteen participants failed to show for their scheduled appointment and never rescheduled (Fig 1), and another fifteen participants called to reschedule their appointment, often at the last minute. While it is not uncommon for anxious individuals to avoid novel experiences, it is worth noting the heightened anticipatory anxiety that anxious patients may have about Floatation-REST, as this creates a clear barrier to entry. One potential reason for the anticipatory anxiety of Floatation-REST may stem from its association with “sensory deprivation”, a loaded term which engenders many historical, and often incorrect, stereotypes related to a loss of control, hallucinations, paranoia and panic [82, 83]. The relaxing and serene state induced by Floatation-REST in the current study appears to be very different (in fact, polar opposite) to the unpleasant and anxious states that were sometimes reported in sensory deprivation research from the 1950’s that did not involve floating in a pool of water [82, 83]. Similarly, Chamber-REST research, which has participants lie in a dark sound-attenuated room, has also found a general lack of negative effects induced by the experience, even in experiments lasting 24 hours in duration, whereas positive benefits of Chamber-REST have been observed across a number of conditions including autism, smoking addiction, and even snake phobia [27]. Given the striking discrepancy between the positive effects found in modern day REST research and negative effects found in early sensory deprivation research, the term *sensory deprivation* has largely been replaced by *restricted environmental stimulation therapy* (“REST”) [82]. We strongly agree with this replacement, as it helps to avoid confusion and steer clear of historical stereotypes that may heighten the barrier to entry for anxious populations. In this paper, we refer to the first term in the REST acronym as “reduced” instead of the more commonly used term of “restricted” in order to further minimize any potential negative associations that may be elicited by this latter term.

### Considerations for replication

As mentioned in the Introduction, there are now hundreds of recreational float centers (http://floatationlocations.com/where-to-float/) where consumers can pay money to float, typically ranging between $40-$100 per session. It is not yet clear whether the anxiety reducing effects found in the different clinical populations studied here can be fully replicated in recreational facilities. In contrast to the open pool design employed in this study, most recreational facilities have enclosed floatation tanks. Enclosed tanks may heighten the anxiety in patients, especially if they have any claustrophobia. In addition, many enclosed tanks fail to ventilate the air near the surface of the water, allowing for the build-up of high levels of humidity and carbon dioxide, which can further heighten anxiety.

Additional precautions were also taken to provide participants with complete control over the float experience, including having the ability to turn the lights on or off using an infrared wave detection system. In contrast, many float centers do not offer this option, and some float tanks do not even have a light. Likewise, all participants in this study received a thorough introduction prior to their float experience by a mental health professional, and were continuously monitored throughout the experience using an intercom system. These factors may have increased the participants’ overall level of safety and comfort, and may have also diminished the base rate of adverse events. Thus, the optimized setting created in this study may have contributed to the positive results.

### Limitations & future directions

Although the findings from this initial study provide early indications for a clinically meaningful signal in anxious and depressed individuals, many important questions remain. The study was limited by its open-label design and lack of a control group. Moreover, since this was only a single-float study, it will be imperative for future studies to explore the effects of multiple float sessions to determine whether there is any evidence for sustained long-term benefit or any sign of adverse effects from repeated sessions. In addition, no studies have examined how long the acute effects persist after a float is over, and a better understanding for the duration of the acute effects will be important prior to determining other factors such as the optimal “dose” of floating. It will be incumbent upon future research to explore how long the benefits last after a single float session, and whether the persistence of such effects changes following multiple float sessions. Replication using a randomized controlled design with longitudinal follow-up will be a critical step for assessing the efficacy of Floatation-REST in anxious and depressed populations. It will also be important to identify a good comparator condition that controls for the effects of expectation and assesses the degree to which the relaxation benefits can be obtained in a setting outside of the float pool. Notably, the selection of a comparator should be based on the specific hypothesis and there is unlikely to be a “one size fits all” solution as the specific target of treatment could change based on the clinical population under investigation. The current investigation targeted state anxiety, and the findings revealed that the reduction of state anxiety was consistently the largest effect across all subgroups and diagnostic categories (S3 Fig), highlighting this metric as a useful target for future investigations.

Despite over a half-century of investigation, the science of Floatation-REST is still in its very early stages. Given the large number of sensory systems altered by the float experience, it is currently unclear what factors are driving the positive effects. Culturally speaking, modern society is in the midst of a technological renaissance that has created a near constant state of connectivity. Addiction to social media and technology has become rampant [84], making it increasingly difficult to unplug for even a single day [85]. And while many find the prospect of being alone and doing nothing for even a short period of time to be aversive [86], the current results suggest that occasionally ‘disconnecting’ from technology and providing the nervous system with a respite from outside stimulation might actually be conducive for mental health. Beyond the potential mental benefits, Floatation-REST also seems to provide clear physical benefits, as our data revealed large reductions in muscle tension and pain, with muscle tension showing the largest reduction out of all the measures (Fig 5). Since the body can harbor stress [87], often in the form of muscle tension and pain, it remains possible that many of the positive benefits derived from Floatation-REST are being driven through the body-based nature of the intervention. Reductions in somatomotor activity and exteroceptive sensory input may also play a role in the positive benefits, but it is currently difficult to discern the precise extent. For example, having the lights off during the float session provided some advantages (in terms of effect size) on measures of serenity, well-being, stress, and negative affect, but it did not alter the overall level of state anxiety reduction, which remained high regardless of whether the lights were on or off. Thus, it seems plausible that the clinical impact of Floatation-REST could be a byproduct or summation of all the different sensory and motor systems that are altered by the float experience, rather than any single system driving the change by itself. Many possibilities exist and future research will need to decipher how floating affects the nervous system in order to isolate the active ingredients.

## Supporting information

S1 Checklist (PDF)

S1 Floatation-REST Procedure (PDF)

S1 Fig Floatation-REST immersion time. Each participant’s total float duration (light blue) and total time spent with the lights off (gray).
(TIF)

S2 Fig Comparison to the non-anxious reference sample. Change scores from pre- to post-float are shown for all of the measures completed by both the anxious sample and the non-anxious reference sample. Error bars represent the SEM.
(TIF)

S3 Fig Effect size estimates for different subgroup analyses. The estimated Cohen’s d is color-coded and displayed for each pre- to post-float change score for the different subgroup analyses. The acute float effects were found to be large irrespective of diagnosis, sex, medication status, and level of visual stimulation.
(TIF)

S4 Fig Floatation-REST in the severely anxious subgroup. Average change scores from pre- to post-float in the severely anxious participants (n = 17) in comparison to the remainder of the anxious sample (n = 33). Error bars represent the SEM.
(TIF)

S5 Fig Subjective comparison of the relaxation induced by Floatation-REST to other relaxation techniques. (A) Percentage of participants (out of the group of 50) who selected one of three possible answer choices comparing the relaxation induced by floating to (B) other relaxation techniques they have tried in the past.
(TIF)

S1 Debriefing Interview Transcriptions (PDF)

S1 Protocol (PDF)

S1 Data (XLSX)
