# Supplementary material for: Examining the short-term anxiolytic and antidepressant effect of Floatation-REST
Source: PLoS One. 2018 Feb 2;13(2):e0190292. doi: 10.1371/journal.pone.0190292 (PMC5796691; doi:10.1371/journal.pone.0190292)
Supplement: S1 Floatation-REST Procedure — (PDF) [file pone.0190292.s002.pdf]

Floatation-REST procedure

In order to minimize bias and demand characteristics, several points were emphasized prior to floating. During the informed consent process, each participant was explicitly told, “this is a research study on a technique that we know very little about, so we cannot make any promises that floating will help or benefit you in any way.” Before completing the self-report questionnaires, participants were reminded, “there are no right or wrong answers, we just ask that you answer as honestly and accurately as possible.” In addition, participants were told that this initial study was focused on learning more about what people actually thought of the float experience, while reiterating that this could only be achieved if participants were honest and forthcoming.

Before floating, each participant watched a short instructional video providing a basic overview of the procedure and was given a tour of the float facilities. All participants were instructed that they could float “for up to 60 minutes” and could stop floating at any time. They were also instructed on how to turn the blue light on and off using the air switch and wave detection system, and told that the choice was up to them as to whether or not they floated in the dark or with the blue light illuminated. A shower, located adjacent to the pool, was used before and after the float session, and all accessories (e.g., unscented soap, shampoo, and conditioner) were provided. At the beginning and end of each float session, the song “Relax” by Blank & Jones (Relax Edition One, 2005) was played through the speakers, signaling to the participant that an hour had elapsed. Participants were instructed to use the restroom beforehand, disrobe in the changing area, take a shower, and then enter the float pool. They were also read the following script: “*Throughout the day, our brain and body are constantly bombarded by sensory information from the external*

world. In this study, we aim to understand what happens when you get a chance to disconnect from this constant stimulation by floating in an environment with reduced levels of light and sound, and reduced pressure on the spinal cord. While floating, try to find a place of stillness of both body and mind. You have complete control throughout the experience and can stop at any time. During the float we encourage you stay awake and when the float is over we will turn on some music for you. There is no rush, so please take your time exiting the pool." After showering and redressing, participants completed their post-float measures followed by the debriefing interview.
