# Supplementary figures and images for: Examining the short-term anxiolytic and antidepressant effect of Floatation-REST
Source: PLoS One. 2018 Feb 2;13(2):e0190292. doi: 10.1371/journal.pone.0190292 (PMC5796691; doi:10.1371/journal.pone.0190292)

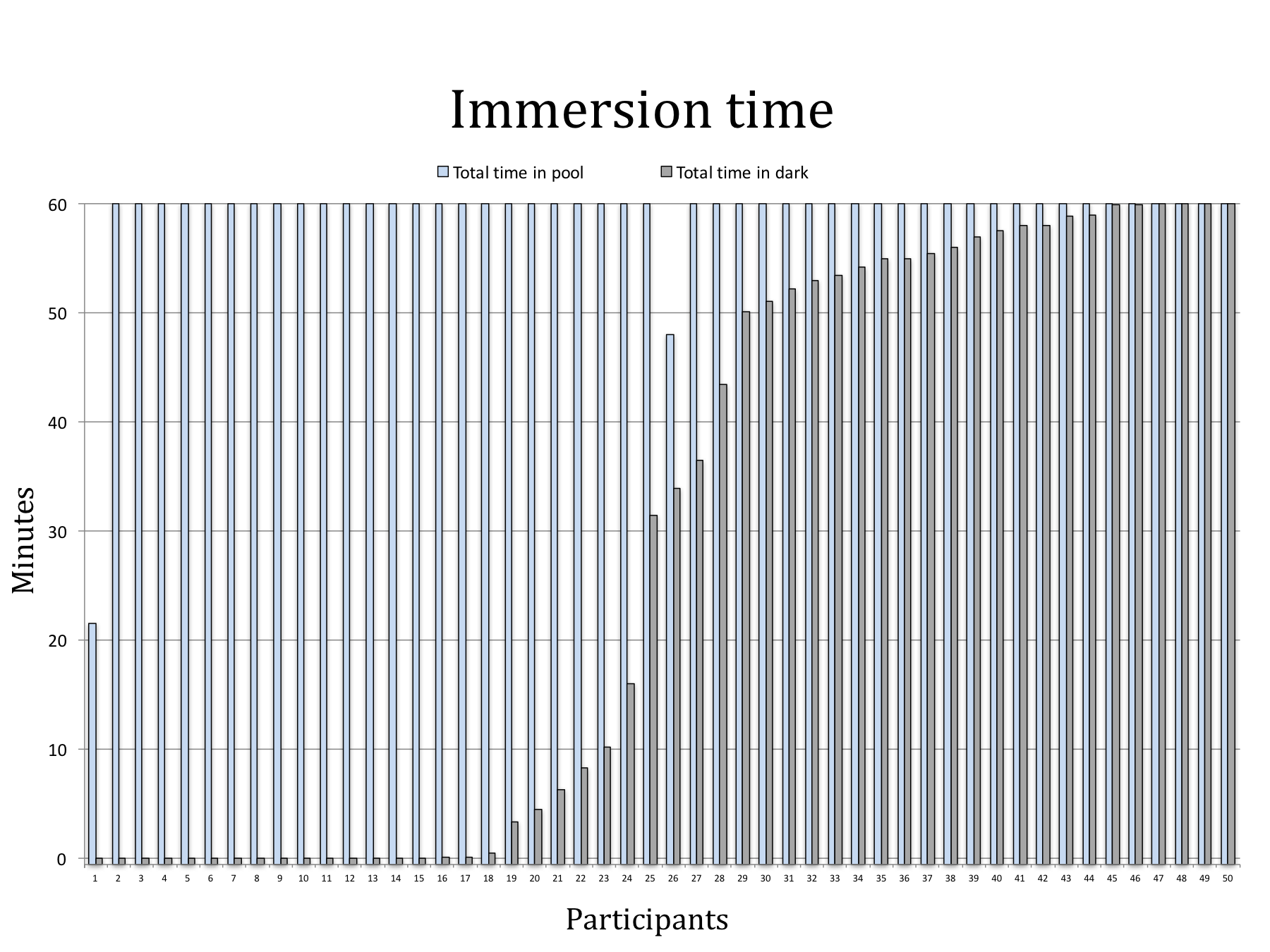

Supplement: S1 Fig — Each participant’s total float duration (light blue) and total time spent with the lights off (gray). (TIF) [file pone.0190292.s003.tif]

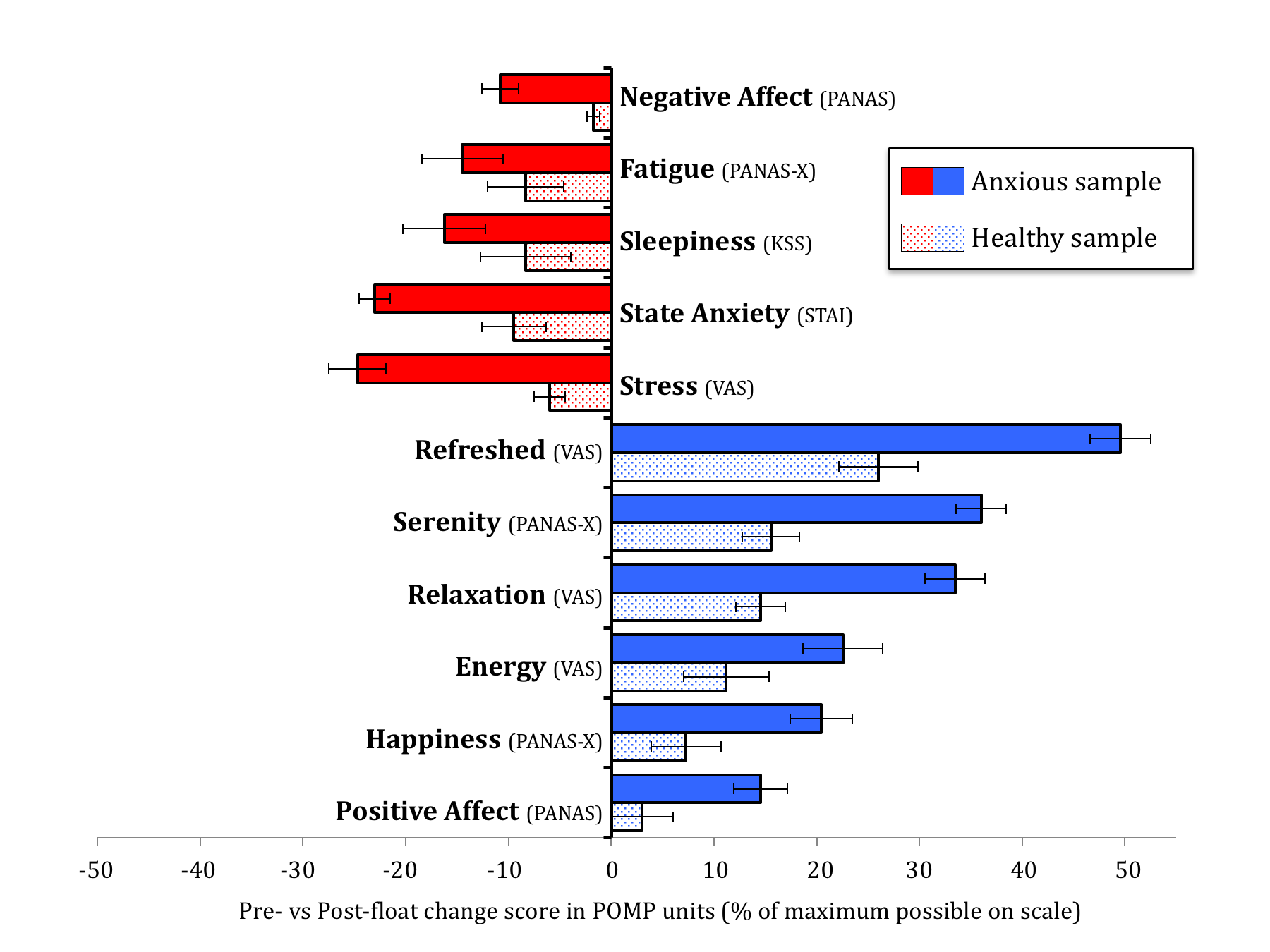

Supplement: S2 Fig — Change scores from pre- to post-float are shown for all of the measures completed by both the anxious sample and the non-anxious reference sample. Error bars represent the SEM. (TIF) [file pone.0190292.s004.tif]

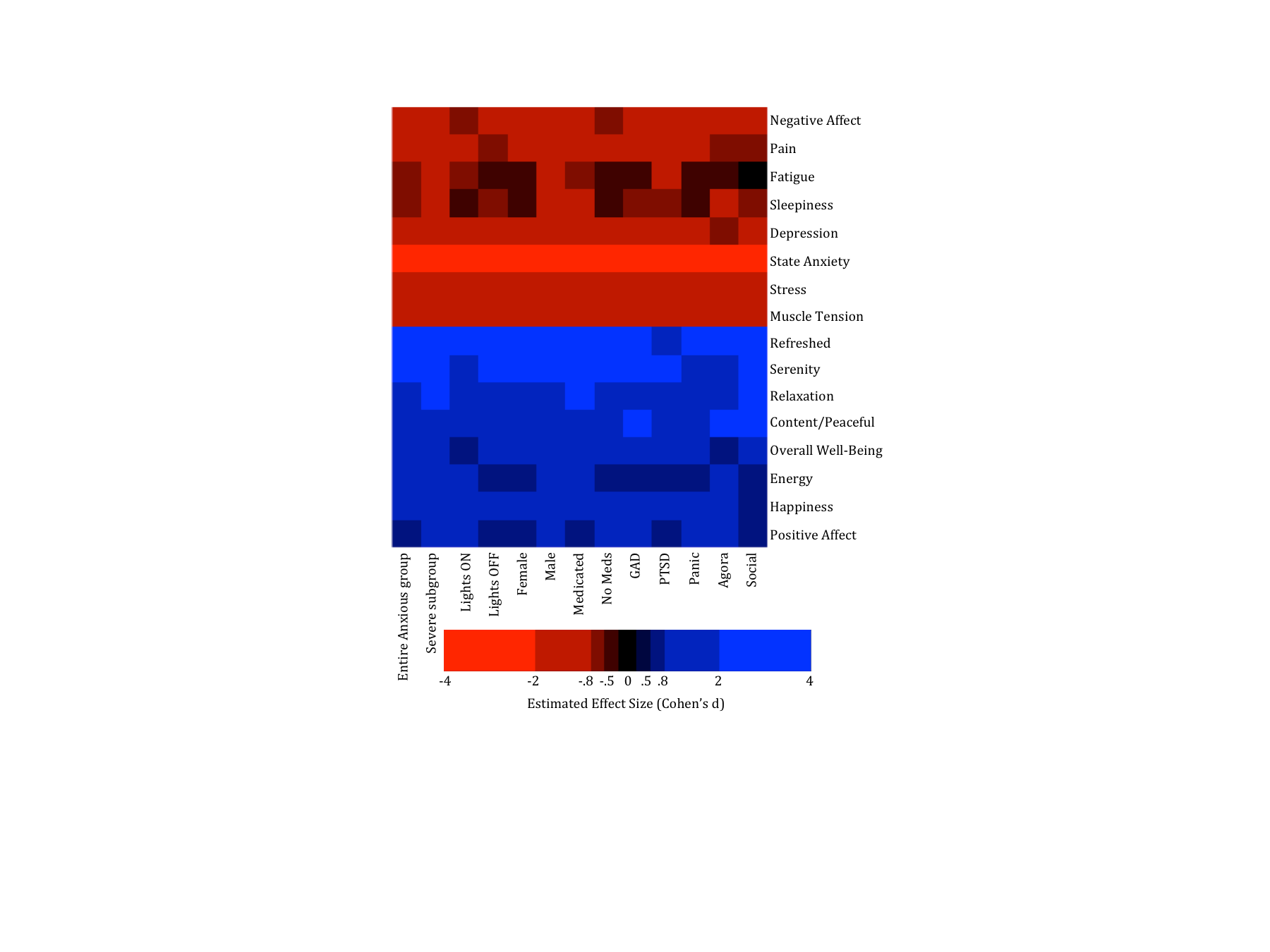

Supplement: S3 Fig — The estimated Cohen’s d is color-coded and displayed for each pre- to post-float change score for the different subgroup analyses. The acute float effects were found to be large irrespective of diagnosis, sex, medication status, and level of visual stimulation. (TIF) [file pone.0190292.s005.tif]

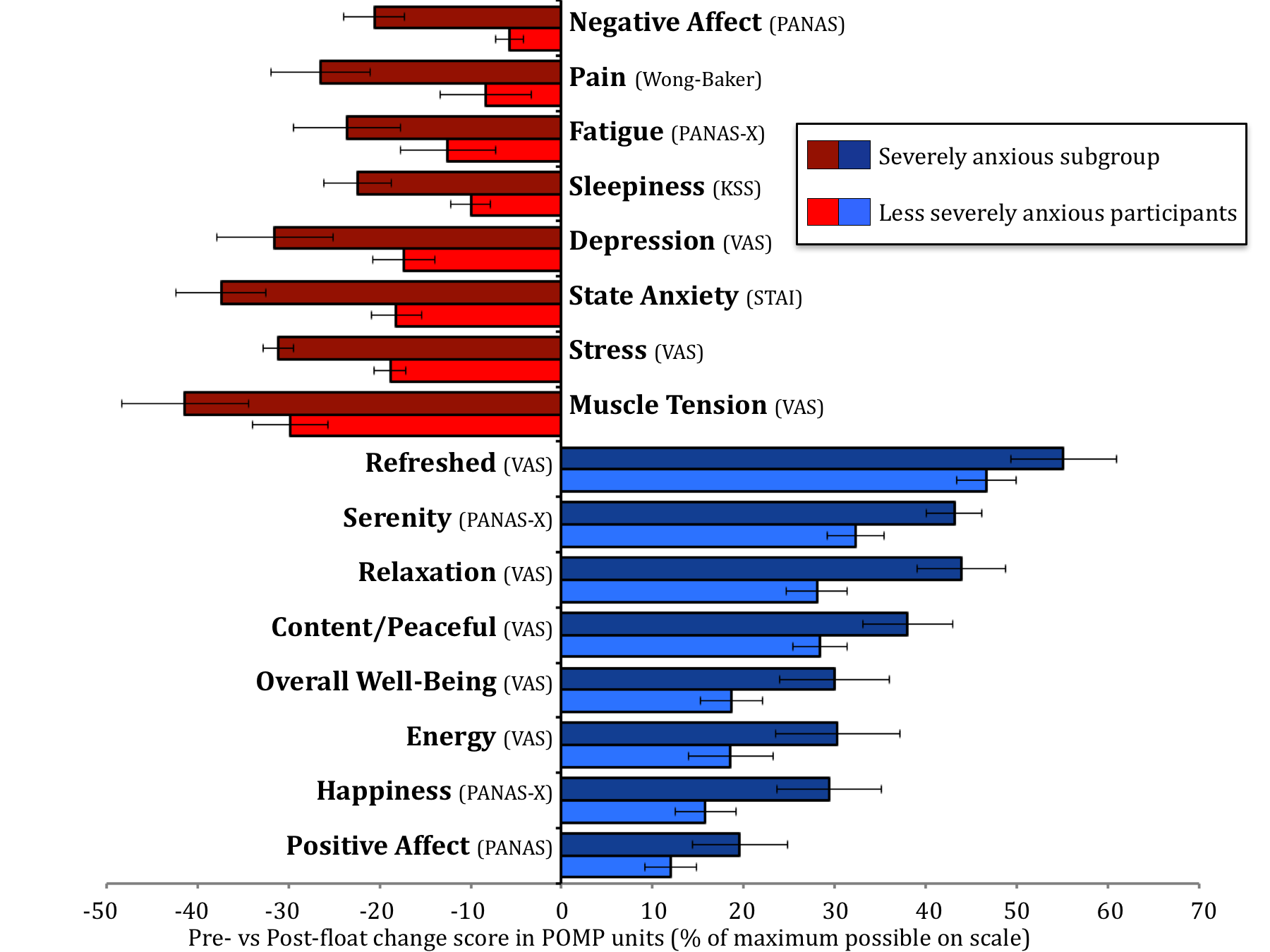

Supplement: S4 Fig — Average change scores from pre- to post-float in the severely anxious participants (n = 17) in comparison to the remainder of the anxious sample (n = 33). Error bars represent the SEM. (TIF) [file pone.0190292.s006.tif]

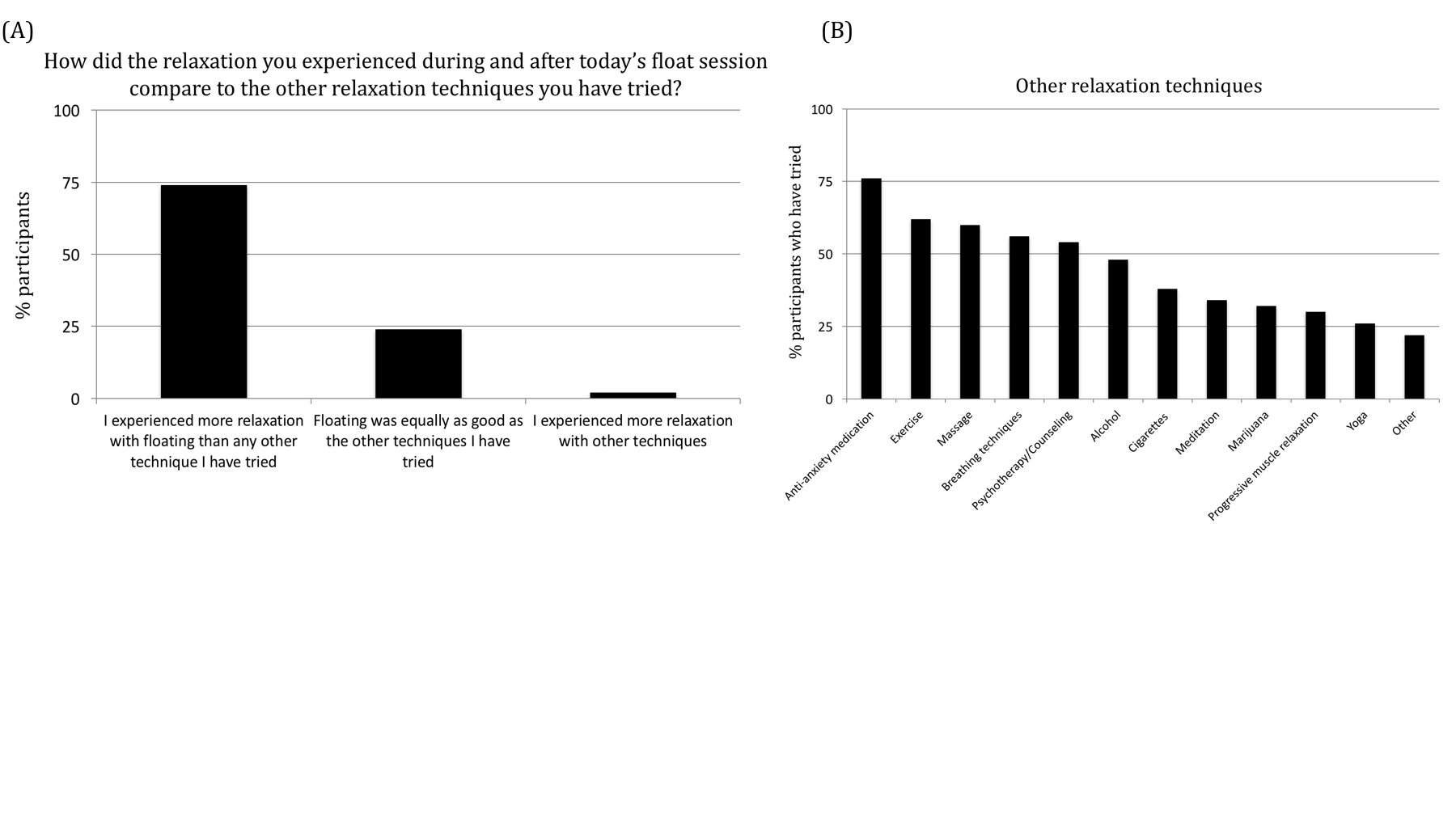

Supplement: S5 Fig — (A) Percentage of participants (out of the group of 50) who selected one of three possible answer choices comparing the relaxation induced by floating to (B) other relaxation techniques they have tried in the past. (TIF) [file pone.0190292.s007.tif]
