# Supplementary material for: Examining the short-term anxiolytic and antidepressant effect of Floatation-REST
Source: PLoS One. 2018 Feb 2;13(2):e0190292. doi: 10.1371/journal.pone.0190292 (PMC5796691; doi:10.1371/journal.pone.0190292)
Supplement: S1 Debriefing Interview Transcriptions — (PDF) [file pone.0190292.s008.pdf]

Overall, how was your float today?

It was pleasant.
—*Subject 33*

It was nice. It felt good.
—*Subject 20*

Good. It was like calm. [laughs]
—*Subject 35*

It's good. It was really good.
—*Subject 5*

It was good. I liked it.
—*Subject 42*

I enjoyed it. I know some things I'd better do differently next time, if you invite me back. Use the earplugs. I normally don't like anything in my ear; only I think I would use the earplugs. And next time, I won't take a shower before I come in 'cause that was three showers today; one at home, one before, and one after. Next time, I won't take a shower before I come in. Other than that, I just wish I'd been able to stay down longer. I think next time, I'll take my meds before I come in.
—*Subject 24*

It was nice. I mean I wouldn't say it totally took my mind off all my stress, but it helped slow down my thoughts and I got to thinking about them like one at a time instead of just jumbling everything up and getting so stressed out about everything at once I can't change. I was able to like sit there and think on it and—I don't know. I liked it though. [laughs]
—*Subject 9*

Well I was okay temperature-wise. It was great. I mean, there were times when I had these like out-of-body kind of experiences that it was like [laughs]—okay, closest thing I could explain to you in real life, but I've never had it—like anything like it. Almost like being inside some kind of virtual trip through space kinda thing. It was really cool. And it was weird 'cause like my eyes were closed, or if they were open it was totally dark in there. But it was like these little starburst things with—I don't know—it was really weird. It was very, very cool. And when I finally found what I would consider my most comfortable position—'cause I had a few that were comfortable, but the hands-behind-the-head thing was like, 'Oh man.' When I found that, I just really relaxed. It was like, 'Okay,' I don't know if I fell asleep then, but I was like in the zone there for a while. And then I got times where I was like, 'okay I'm gonna stretch a little bit'—and I found though, that the more I like tried to stretch then I became more fidgety. So if I could stay the way I was before… Plus, it was very good like 'cause I have a lot of times like back and joint stuff; and laying in there, I did not feel any like pain in my joints or anything. And that was really good. I mean there were a lot of different positives. I can see why people wanna do it. It was weird at first, because trying to get used to it and the salt part—sometimes cause like if you turn—do whatever, and the salt gets on you and then it kinda dries up, it becomes a little bit itchy at times. It's not bad. It's nothing like having a rash, but just, you know? It's not painful or anything; it's just slightly itchy in spots. Probably 'cause my skin is really dry from the winter too. That probably doesn't help it. I would advise

people, if they were coming in here, for like the week before, moisturize their skin so that their skin's not quite so dry. Even though I didn't get my whole face, you know, at parts, it feels—and my hair feels better. It's really weird.
—*Subject 16*

It was wonderful. Really good. I really, really, really liked it. [deep breath] Yeah, it was great.
—*Subject 30*

Weird, but good. Kind of just like I couldn't really quiet my mind as well as I hoped I would. But overall, it was different than I expected. I didn't think it would be like as pleasant as it was with the temperature being regulated and all that.
—*Subject 40*

It was great! It was wonderful. It was kind of like taking Klonopin or Xanax without the side effects; just feeling like alert—not that fogginess, you know, feeling sluggish or just like I just wanna withdraw. It was just great.Yeah! Yeah it was, yeah. Unlike anything I've ever felt. So it was good.
—*Subject 2*

Oh it was awesome! I feel so relaxed and I just loved it. It was so awesome! I think I may even have fallen asleep. I don't know. [laughs] It was awesome. It really was. I've never been so relaxed. I felt a little tension, like you said, in there before, but I just let go—just let it go! And it was ugh! Awesome. I'm so amazed by how my body's feeling still! It's lasting too—longer than—I got a massage one time and I've never felt so relaxed. This blows it away! I mean I feel so relaxed. I mean my mind is still just like, 'Hey, it's so relaxing.' You know, I don't know how to explain it any other way. It's just my whole body and my mind—I feel so good right now. I'm not in pain. My knees usually some, and my back usually hurts. I don't feel anything right now. I don't feel any pain or anything—nothing. I mean, I feel I've never been this relaxed. I feel so awesome. I really do! It's better than a pain pill. It's better—oh my g—I can't believe it! It really—it's freaking me out how good I feel. I really do. I do—I feel so... I mean, somebody could come in here and [laughs] jump on me and I wouldn't get mad. I wouldn't—I would not get mad. I'd be like, 'Go ahead!' [laughs]
—*Subject 38*

It was pretty good. I liked it.
—*Subject 10*

It was fairly relaxing, but I don't know if that neck tension makes sense for other people. I mean I put my hands behind my head a couple times if when I thought it was starting to get a little tense, but then I took 'em back off there and relaxed again so.
—*Subject 43*

It was pretty good. I thought it was mainly gonna go—like felt good at first, and then like get boring, then feel like I would have to like kinda like push myself to like stay there. But I think it was nice at first but kind of and then it did, like for a little while in the middle there, it was kinda just—you were kinda trying to like—it felt like you were trying to push through it or force yourself to relax a little bit

more, but then like once you kinda just kinda pushed through that, it kinda gets like—then you just chill. [laughs] Yeah it does, but once you—I got to that point, it felt—it was pretty good.
—*Subject 4*

It was amazing. It was like floating on air. No cares in the world. No worries.
—*Subject 14*

Very relaxing.
—*Subject 37*

The last 15 minutes I think were good, but the—the first half was unexpectedly anxiety-inducing. Like when I was taking a shower, it was fine; but when I first got in, like the panic set in and I had a hard time making it go away. My heart was racing. I felt a little bit tense. [Experimenter: How long do you feel like that sensation of panic lasted?] About 20 minutes and then it subsided after that. But I had to put some effort into calming down. I tried to figure out what it was—like, 'Was it just the floating or the light or the size of the room?' But I felt like if the size of the room or the pool were bigger, I'd have even more anxiety; or if it was darker—like I didn't turn the light off at all. The blue one, I didn't turn it off. I think one of my fears was that when I was floating, I kept splashing around and trying to control my environment because I didn't want to let go; I didn't wanna slip into the feeling you get when you're in sensory deprivation. Like I meditate and I enjoy that, but I can always move my arms and wake up and go do other things around my house. I think this was a little bit different. But when I was finally—like in the last 10… 15… 20 minutes, when I was finally enjoying the float, that felt very much like I was meditating.
—*Subject 22*

It was pretty good. Very calming and soothing. Good.
—*Subject 31*

Very relaxing. I mean really relaxing. I did do some breathing because with the earplugs, it's easier to pay attention to one's breathing and my heartbeat. I could feel my heartbeat in my ears, which is weird. But then I would drift off to sleep, and then I'd twitch. [laughs] You know? 'Cause every time I—you know. But so, you know, I did use some of the time to do some jewelry designing and, you know, that sort of thing; but then my mind just sort of drifted off and then I'd fall asleep and then I'd twitch and then I'd wake up and then I'd... [laughs] and then I'd breathe and then I'd, you know, drift off, twitch, wake up... [laughs] But like one of the questions in the survey was like, 'How do you feel right now?' and I'm kinda like man I'm just kinda like I either just had an hour-and-a-half massage—not the hour! The hour-and-a-half massage; or I had a really nice nap. 'Cause I'm just—I'm not foggy. I'm pretty neutral, but I'm really relaxed, you know? I was gonna write in there, 'like butta,' [laughs] but then I thought, 'No.' [laughs] I normally don't, but it was saying like 'describe' and I was like, 'I feel like butta!' [laughs] But that's pretty much, you know, very comfortable; the water temperature was perfect. What did you put it at? Do you know? [Experimenter: It ended up going closer to 95.] Okay, that's fine. That's perfect. And the water—you try

to keep your water heated at the same temperature. And so yeah. So that was perfect. I mean there was times where I couldn't tell the difference between the water and not the water. So then, if I felt even just a touch warm, I'd just push off from the side and then the water would slightly cool. And then I'm like 'Ah, go back to sleep.' [laughs] So that thing's great. Seriously, you oughta hire it out! [laughs] I mean cause the break from the external stimuli is necessary; and I think that's what meditation is trying to achieve. When you've got all this crap going on around you, well stick 'em in a float pool! I ended up not turning the light off, but it didn't make a difference to me, you know? Because I kept my eyes shut just in case, when I twitched then I'd get—you know, well, you know what I mean. But it wasn't a light that would bother me at all, you know? So just no big thing.
—Subject 29

It was really good. I actually thought I was pretty relaxed before I went in, but when I got out, I'm like, 'That was pretty amazing.' It's really weird. I don't know. I have had a similar feeling like when they give me like conscious sedation or something when I'm first like—I feel like everything is gonna like cramp up. And I have to like really concentrate on relaxing so that it… so I guess I must have a lot more tension than I think. [laughs] [Experimenter: It gets built up. And the brain has a way of sort of holding on to it.] Yeah! I could—well that's a thing I could really feel in there. But I think I was able to get there. And I could feel the back and neck tension and everything, but I think I resolved that pretty quickly. [laughs] I just had to remember to like let my head hang. [laughs] Well I did get relaxed enough that I think the right side of my head is heavier 'cause I'm kinda goin like this and I actually did get some in my eye just because I like... It was fine though. It wasn't a big deal so. I think that was the only thing, at the time, I did.
—Subject 36

Incredible! [laughs] I would recommend it. I mean I've never had chronic pain, but if people maybe have chronic pain, I mean there's like a weightlessness feeling. I mean, I just 'incredible' is the only word I can think of. I kinda did something a little bit that, to me, was euphoric; as I was laying there, of course, I figured out the light finally. Round peg, square hole. When I was laying there, like you know, just like this, if I bumped the pool, I'd kinda just ever so slightly just push myself off and I could feel like my hair, you know? And so I mean it just felt like I was just bobbing in the calm ocean. I mean I kinda liked the—cause like I told you, I'm still in the very beginning stages of meditation; and so to me, that freed my mind—was just laying there and just kind of ever so slightly pushing off and then I'd just… Yeah I had no idea where I was and then, 'ope, here's a wall!' and just pushed away from that. That was very therapeutic! I'm like I don't know if anybody's tried that, but… [laughs]
—Subject 1

It was really good. I really enjoyed it. I could go back in there for another two more hours. The time went by fast a little.
—Subject 44

Overall, how was your float today?

It was really good! A lot of it, towards the beginning, I was having a really hard time settling down. And I knew like as soon as you had said some people have issues with, you know, their neck having tension; I knew I was gonna be one of those 'cause for some reason that's always what I go to. And I was like 'okay.' So I was prepared for it. Ready to try to relax. And I felt like I did for a while, but then for some reason, I felt like my neck was getting tight still, and so I was like, 'Well maybe if I just, you know, try to move around or, you know, maybe it's just, you know feeling creaky for being in the same spot.' I don't know; and um so that was the only issue I really had while floating, but it was pretty good for the most part.
—Subject 13

It was good. I enjoyed it. It was relaxing so…
—Subject 12

It was pretty good. I got kinda bored, I think, the last probably 20 minutes. Besides that, it was okay.
—Subject 47

It was wonderful.
—Subject 3

It was good. [laughs] I had a lot of like anxious thoughts and worries like constantly. And I had to constantly remind myself to calm down and relax. It was difficult at first, and then like I kind of just relaxed; and then like I felt like I lost track of time and I was like, 'Well, how long have I been in here? I feel like I've been in here a long time.' And then I got like—I started to get really worried and really like irritated and I was just like, [deep breath] 'Okay…' But throughout like the whole time, I had to keep turning the light on and off because it would become like kind of a nuisance. Like too bright. But then, I would really worry about like not having a light on. I couldn't see my surroundings. I lost track of my position in the water. It freaked me out. And I had to like touch the—I had to put my feet on the floor a few times so that I could like ground myself so I knew where I was. I had to keep doing that. I had to sit up a few times and get water out of my ears so I could hear. I got really anxious I guess! [laughs] [Experimenter: How did your neck feel?] I had to keep moving it. Like it stiffens and like builds up the cartilage; like builds up the pressure if I stay still for too long. So I have to move it to pop it. And that happens in my shoulder too 'cause there's some tension points behind my shoulder blade that are also tense nonstop. So I had to keep moving my arm up and down. I was really restless. I'm that way normally anyways though. I move around a lot. I did have moments where it was really enjoyable. I liked being able to stretch out and not have the pressure of like pillows or a mattress or a chair on me. That felt really nice. [laughs]
—Subject 7

It was different. It was relaxing.
—Subject 8

It was good. I enjoyed it.
—Subject 10

It was good. You know, it took maybe a minute or two to kind of get acclimated to the feeling of it. And then I kinda tested the light being off and on a couple of times and I think I ended up just leaving the blue light on. And then I tried, you know, leaving my eyes open

and closing them—just kinda testing everything but, overall, it felt good. I didn't really feel any neck tension or anything. But I could see kind of like when I tilted head up, where—but I was able to kinda get in a real comfortable position and just tried to kinda relax.
—Subject 17

It was a really nice experience. At first, I noticed the neck tension you were talking about. I'd realize it was tense and so I'd try to relax and then I'd find myself like holding my head up again and I'd have to relax. After a while, I was able to—it felt like I was kinda completely relaxed. I would be fine like and just laying there and then I would get kinda restless in a way. I feel like that's just cause I'm constantly used to—going, going, going. I don't usually take time to like waste time, you know? [Experimenter: And just to have a moment to yourself is kind of unusual?] Exactly, yeah! So I was kind of like—my body just felt restless, and I was kinda thinking about, 'Well, what do I need to do?' Like this and that. But overall, it was really nice.
—Subject 18

It was different! I didn't really know what to expect going in there; but you know, once I was able to kind of relax into the water, it was kinda difficult to determine—especially if you don't move—kind of, you know, where the water stops and where you are. And so it does have kinda that—I don't know if 'detached' is the right word, but you do kinda have just that feeling of, you know, you're not quite sure where your position is in space. But it's not a feeling of fear. I mean it feels very secure. So I floated a little while with the light on and I thought that was fine cause it was so dim. And then I tried it with the light off for a little while. And I think, personally, I did a little better with the light on. It's simply because with the light off, I just found it—you know, mind was kind of looking for something to focus on a lot. And to the point of okay well I'm like, 'I think I need to move around a little bit.' It was very difficult to stay still for an extended period of time with the light off; so I would turn it back on and it was easier. I found it was easier for me. And so after I had the light back on for a little bit, I decided to try a little bit more with the light off. So I did. And it was easier the second time around, but still found myself kind of restless. And so I felt like it was more relaxing for me with the light on.
—Subject 19

It was really good. Strange. I would describe it as strange. At some point, I lost physical sensation and that like—at first, it was really, really odd; and then I kinda just accepted it because there was no reason not to, I guess. I don't know. And then I was—I'm not sure I was asleep. I don't know if I was asleep, but it was like time went by really fast and the thoughts—any thoughts I was having were dreamlike. And so it was really interesting in that regard. But I have never had like—I mean there were moments where like I was very aware that I could not feel my extremities and I couldn't feel anything. And it was like—I don't remember. Other than maybe being under anesthetic or something before like a procedure; maybe before like dental. I don't know. It was like you were completely un—unplugged or removed; I would use the word 'removed,' 'cause I don't

know how else to—that's kind of a—yeah, so yeah! And then, like whenever I got out of the tank, I was doing everything, getting dried off and everything; my mind was clear and I knew I had to take a shower and dry off and get it all, you know; and so I went through those motions like I normally would and then, like the whole time, I'm realizing like I'm still like not a hundred percent feeling like—well I say 'feeling'—I guess the word is 'feeling.' But it's not that I was in pain before, but it's like there was even less pain; like or there was even less tension—I don't even know the word! Because I wasn't in pain before I went in there; but then, whenever I came out, it was like this gradual transition to I would say—this is a good way to put it—like at one point, I went numb in there and then slowly, the numbness wore off and it's still wearing off. But it's like it's still there in a way. It's a very strange thing. This is the first time I've ever felt anything like that that wasn't involving like medicine or something, you know? So it was very, very odd, but nice and I would say also—and I put it in that—in the questionnaire; for some reason, I felt appreciation because I was in such a pleasant state. It was interesting too.
—AO035

It was good. It was good. It was very relaxing. I think I may have fallen asleep a little bit, it was a little bit harder to quiet my mind. I was thinking about what I have to get done next and today and what's going on, whereas usually it's afterwards. It's after I've already—I've gotten pretty good at not worrying about tomorrow. It's what's next in the day. So I did find myself going back and trying to return to my breath because I noticed that I was really gnawing on the afternoon and what I had to get done. And I think, for me, it's not a get-ready-for-the-day activity; it's an unwind-from—wind-down kind of activity.
—AM244

It was very relaxing. It felt safe. It didn't feel—I mean not having anybody else in the room was nice. It felt very private and secure. And being weightless was kinda nice.
—Subject 21

It was very nice!
—Subject 27

I actually really enjoyed it! I decided to not do clothes. I figured if we're doing it, we're doing it. It didn't seem to matter. At first, of course, my face itched everywhere, you know? Not touch it, so it itches. I initially was there and eventually decided to turn out the light; which I don't know if was necessarily a good thing because then it was much easier to kinda clear my thoughts without everything to distract and be able to focus on… I may have nodded off for like a second or just like started—I don't know if I started to fall asleep and jolted awake. I would guess somewhere like 20-40 minutes time frame to get relaxed. I think I finally found the sweet spot to not hold my head up. But it was really nice. I mean once it was dark, yes, of course you want to go to sleep because that's the only time you are still in the dark.
—Subject 28

Good!
—Subject 34

Overall, how was your float today?

It was awesome! I really liked it.
—Subject 39

It was actually amazing. I was, like I said earlier, a little nervous about it, but it was really amazing! I was really surprised. And yes, there was no way that I couldn't have floated! [laughs] Like I did, I felt like a little [inaudible word] picture. It was awesome.
—Subject 45

It was a very pleasant experience; to the point that I would want to have one of those in my house. I really, really liked it. What I wrote in the questionnaire, or the survey, is that what I like about it is that it's so natural. And so I was kinda concerned about the floating 'cause I kinda was a little nervous about that, but I was amazed at how quickly my body just rose to the top of the water like I would even lift my leg up at one point, but my head was still above water and I thought that was really cool. But I was just wanting to test it out to see what would happen [laughs] But yeah, I did what you were saying to do as far as like moving my head around to try to relax my neck and one thing that I noticed is that my head felt really heavy and it was almost like a blood-rushing-to-my-head type of experience. It didn't last very long. Probably maybe five minutes or so. And I even put my hands behind my head too—and towards like the very end—I'm not sure how long I was asleep, but I did fall asleep. And I think I like the lights off too, because I noticed I had my eyes open whenever the lights were off and then I had my eyes closed and it just kinda seemed like there was no difference whether or not… And so I felt like it helped me to really focus and just kinda—I was really trying to use the opportunity to just let go of a lot of stress… But it was, overall, a great experience. I definitely would do it again.
—Subject 46

I really enjoyed it.
—Subject 48

It was great! Very relaxing. And I wasn't really aware of the water at a certain point and it was really nice.
—Subject 49

Perfect. [laughs]
—Subject 50

What did you think about, if anything, while you were floating?

Oh, jeez. Everything and anything; planting my garden, talking to God, thinking about my kids. Yeah, just mostly thinking about [how] I could hear myself breathing.
—Subject 33

I tried to just practice thinking about my breaths. I learned a cyclic breathing like in through your nose, out through your mouth. So I tried to do that the whole time.
—Subject 20

[laughs] Just absolutely nothing.
—Subject 35

The first part, it was fairly easy to keep my mind fairly clear, but as time progressed, it was hard to quiet the chatter and stuff like that. [Experimenter: In terms of the chatter, would you rate it as more positive thoughts or memories or more negative?] It was just more tasks of what needs to happen today. And more stresses of what the rest of my day looks like after I leave. And things like that. [Experimenter: How did you cope with those sorts of thoughts inside the pool? Was it different than having those thoughts outside the pool?] You know, I kept trying to let my mind go and then bring it back to quiet it down, but after so long, I just sort of fell into it and started out, 'Yeah I need to do this, I need to do this. This is expected to happen.' And all that sort of stuff too.
—Subject 5

Just like past memories of like my dad and going to see my new nephew and everything. [Experimenter: What was it like to experience the memories in that environment? Was it different than having them out here?] Oh yeah, yeah. [laughs] It was a lot different. It was kinda of like just being there and I guess that would be the best way to put it. [Experiment: These are old memories of your father and stuff?] Yeah. [Experimenter: Were they positive or negative?] Positive.
—Subject 42

Everything was going very slow. Even the thoughts. Everything just slowed way down and it was kinda like, 'Oh, let me get through this! This pain is gonna get me!' [laughs] The pain in my shoulders is always there. And my neck 'cause there is nerve damage in them, but other than that…
—Subject 24

Mostly like the stress with our little house fire and stuff. And losing the kids after that. But it wasn't like unbearable like how it is every other day when I'm thinking about it. It was—I don't know—like I could think about it without getting depressed or feeling like I wanna just take a shot or pop some pills.
—Subject 9

Oh just the crap from today mainly. And my work. I've had a lot of stuff going on with my work; trying to decide what I wanna do next year. And right now I'm teaching and coaching. I've been trying to decide whether, now that I'm here, whether that's something I wanna do. Our school's trying to—there's just lots of change—stuff that's going on, and it's been going on a lot in the past probably

month or so, because in education, this is the time of the year it starts, you know—till you figure out what you're gonna do next year and I don't know. And then today, my principal got up and said about that thing, and so it's just kinda—it's just kind of re-stirred all of that stuff and it brings about a little anxiety. [Experimenter: What was it like to experience those thoughts in the pool versus out here?] I would say that I didn't—my body didn't probably get as tense because it was like forcing my body to relax even though my mind was going, because when I do it like out here, your body has these normal reflexes that, you know, the neck and the shoulders and different… And another thing I do a lot is I grit my teeth and I do all that stuff; and I noticed in there I wasn't doing that as much. It's kind of like, like I said, almost like putting your body in a tranquil state while the mind is still working. [Experimenter: It's a very interesting juxtaposition between those—] Yes! Versus the fight of the environment. There, you're not fighting that environment. And because there're so many distractions all around you, whether it's noise, or visual, or auditory. And in there, there's not that. So I think that's where sometimes clarity and things like that can be from.
—*Subject 16*

I did think a lot about my bike. And just the road. And a student, random client [Experimenter: So no real negative thoughts or anything?] No. They were all really positive thoughts, you know? Or good memories; like how the questionnaire said. Nothing really negative. Nothing uncomfortable. Just the queasiness was the only uncomfortable feeling.
—*Subject 30*

Anxiety that I've been kind of focused on today, specifically, and stuff I need to do when I'm home. That's pretty much it. [Experimenter: Did those thoughts feel any different in the pool than if you had them in day-to-day life?] they came a little bit clearer, 'cause I—my mind was able to focus on them specifically and not like, you know, thinking about work things as well as that being in the back of my mind. They were more in the like forefront of my mind. [Experimenter: Did you have any difficulty coping with those thoughts?] Not any more than normal.
—*Subject 40*

Letting go. I kept thinking about... just I felt so grateful. Grateful for the experience. Grateful for just how wonderful it felt. Grateful for just… it's kind of like life happens here, and you can just go through a door and not take part in it. You know what I mean? Just separate yourself from life for just a little while, you know, just pausing. Kind of like, you know, like in movies, like you press the pause button and you move and everybody else… like life just stands still. Like that. It just felt like life stood still. I remember feeling grateful. And the letting go part was more of—I would just keep sensing my body while I was breathing, trying to feel where I was holding on to tension. And you don't realize it because you relax and then you concentrate on your feet and you go and you tell your feet to relax. And you can feel, 'Okay it's relaxing a little more than I relaxed before.' So I kept doing that. And every time, I just was able to relax more and more. So that was good… And at one point, I felt like I wanted to move, just slowly, from side to

side. Just like, especially when the music came back on, it felt like I just wanted to—I felt happy! [laughs] [Experimenter: Did you have any negative thoughts or memories during the float?] No. I was just—I felt totally present. I didn't think about—deliberately, did not want to think about anything else. It was a gift to me, so I wanted to use it while I was there.
—*Subject 2*

Honestly, I was just trying to relax. My main concern was I was trying to relax. Just let go. Relax. That's what I was trying to do.
—*Subject 38*

Thought about my day for little bit, at first. But then, eventually, just kinda tried not to think about anything. [Experimenter: Did any negative thoughts or memories come up?] No.
—*Subject 10*

Oh, I put it on a deal. Probably like thoughts about the future. Thoughts about spiritual stuff. I mean it was pretty—not really focused on the present or worrying about present stuff; just kind of wondering about the future and, you know, like I said, spirituality type stuff kinda played in. [Experimenter: Was there anything different about having those thoughts while you were floating in the pool versus having them out here?] I don't know. Maybe I didn't dissect them as much. I probably just like opened myself up to something to come to me and then just was at peace with it after that, as opposed to trying to break it down. Like if I was sitting at home, you know, in the daylight or something, or just with the lights on—TV on or something like that, I might sit there and kinda dwell on it and try to break it down more. But while I was floating and feeling calmer, I was just opening myself up, waiting for something to kinda come to me or wishing for it to come to me. Kinda something like that. I think about that off and on throughout, you know, every day. I think about spiritual type stuff; though I mean just not your run-of-the-mill regular, I don't know, traditional religion type stuff. Just I don't consider myself a religious person, but I consider myself a spiritual person. You seem like a pretty intelligent guy though. I don't need to explain that to you. Religion sucks and spirituality is awesome. I think because religions start wars.
—*Subject 16*

I mean not really in particular. I felt pretty present. I don't know. I wasn't—I mean like there's like a couple like thoughts from past or like worrying about the future, but like not very much. Like they were just kinda brief and they would just kinda pass by.
—*Subject 4*

At first, my mind was just kind of like everywhere, but once I calmed down and just let the water take over, I didn't think about anything. [Experimenter: Did any negative thoughts come up during the float?] No.
—*Subject 14*

I thought I was in the ocean. [laughs] I thought about all that peaceful stuff. I didn't have any tension in my neck, where I had to do that. It just came automatically.
—*Subject 37*

What did you think about, if anything, while you were floating?

In the first twenty minutes, when I was very anxious, I was trying to figure out why I was scared of my environment. So I was trying to imagine like—or figure out exactly what I was afraid of. So I was going through possible scenarios in which it could end poorly to try to figure out which one was bothering me. So things like that, at first. But then, towards the end, I was trying to—when I was trying to calm down, I was thinking of things that calmed me down. [Experimenter: When you were having those anxious thoughts, inside maybe during the first part of the float, did those anxious thoughts feel any different than having the thoughts outside the pool, or was it kinda the same?] It was very different. I'm not normally an anxious person of my environment. [Experimenter: So it really was kind of learning to feel safe in this new environment?] Yes.
—*Subject 22*

I had some mind chatter going on, but I couldn't really—I wasn't, you know, not any specific thoughts.
—*Subject 6*

How much everything is going to be uncomfortable when I leave that room. I'm serious! It's, you know, a flood of sensitivity and just all this other stuff. And it's like somebody is putting a wet blanket over you all the time. And just, you know, and just kind of feeling like how sore my muscles are. And, you know, there was a point where, when I was floating, I lifted my arms up and I had this opportunity to feel how much difficulty I was having with my strength; and so, you know, that was kind of discouraging. But I used to swim every night; and so I think this is actually going to prompt me to go get [laughs] a membership for swimming again. And it gives it an opportunity to work all the muscles at once. But it was just a relief.
—*Subject 11*

It's just like—I don't know. It's hard to say. I mean I guess you're there, but, you know, you kinda just feel like you're not, you know? I guess you're just kinda lost in the moment. I mean, it was nice. It was very calming. [Experimenter: You weren't necessarily thinking about past things or future things?] Not really.
—*Subject 31*

You know how when you're falling asleep, just random thoughts go through? And I don't even remember them. I don't—yeah. Just random stuff, and then random thoughts, 'blah blah blah,' twitch! [laughs] Jewelry design, random thoughts, 'blah blah blah.' Twitch! So…
—*Subject 29*

For a while, I wasn't thinking about anything. And then, when I was thinking, it was actually pretty good. Just like envisioning things that I wanna see. So it was pretty positive.
—*Subject 36*

[laughs] Just that I'm not used to going for long periods of time without communicating with my husband. So that was kind of a little bothersome, but he's a big boy. He can take care of himself. That was probably the only thing I really thought about, was, you know, I hope he doesn't need me for something.
—*Subject 1*

What did you think about, if anything, while you were floating?

Nothing really. I did fall asleep a little bit. I was relaxed and I was just thinking…
—Subject 44

A lot of what I was thinking about was kinda the future of like, oh, things I need to do this week. None of them were really positive or negative thoughts. A lot of them were more just neutral. Almost kind of like a time to gather my thoughts 'cause a lot of the time, I'm typically more scatterbrained. And while I normally remember to do everything, it's always in a weird order; or, you know, I'm in the middle of doing something that needs to be done and I'm like 'Oh shoot! I have this, this, and this that I need to—' you know? [laughs] This was kinda nice cause it almost gave me a chance to kinda plan out what I'm supposed to be doing this week. [laughs] And I don't know if that's what it was supposed to do, but it helped me in that way at least!
—Subject 13

It started off with, you know, just kind of the normal thoughts that I have. Just, you know, 'What's going on at work?', 'What's going on at home?', 'What do I need to do when I get there?' And then, as I kinda got relaxed into it, I was able to kinda push those out and kinda really just focus on just being there and it was really kinda weird cause like when I got to that point where it was really easy to keep those outta there, I started getting like a light was like a halo effect kinda. It was like wrapping itself around my head and it was kinda narrowing as it went along. And so like just kind of imagine, as one of those thoughts would come in, I would just imagine it going [laughs] into the center of that. [Experimenter: Had you ever experienced anything like that before?] I hadn't, hm-mm. So it was kinda strange at first; it was like 'Eh!' [laughs] [Experimenter: And it lasted a while?] It did. Yeah. And I was able to like—I stopped—I think I coughed halfway through; or I don't know—it felt like halfway through. And it kinda knocked it out, and I was able to get it back. And so that was kinda cool. So I was actually kinda disappointed 'cause I was like, 'Oh, it's kinda fun to watch.' It was like a really slow strobe light. So yeah. So mm-hm, weird.
—Subject 12

I just thought about like my vegetable garden. [laughs] Like, 'What am I gonna put in my vegetable garden?' But I tried to keep it positive whatever I was thinking 'cause I know I can't stop myself from thinking. You know, I tried so at least I thought, you know, I'd make it positive, you know? Yeah, I didn't have any negative thoughts so that was good.
—Subject 47

I did have swirling thoughts. It's not like I went complete still-of-mind, but it wasn't negative and it wasn't intense or chattery. Very relaxed. And that's not very norm for me so.
—Subject 3

I had to keep reminding myself to calm down; and like grounding myself. And then I would worry about like things I had to do, and school, and summer classes, and babysitting, and my sister's moving, my friends… [laughs] [Experimenter: What was it like having the mind chatter while you were floating versus say having it out here in the normal world?] It was like the same thoughts

that I have, they were just kind of like further away; like hazier whenever I was floating. And I didn't really have anything to distract myself, which was really irritating. [Experimenter: So you're used to having the thoughts, but then you can move to something else and thoughts can be temporarily moved away?] Yeah. But they were still kind of like hazy 'cause I knew they weren't supposed to be something I was thinking about I guess. I'm not really sure. They just, I don't know, felt hazy. [laughs]
—*Subject 7*

I don't know. Just the normal, I guess, stresses. And then, I don't know. I think, at one point, like I just kind of felt one—I don't know. Almost like—I don't know—I kinda kept thinking, you know, that maybe this is what it feels like to be in utero, you know? Like I don't know. I don't know. But it was a bit challenging to try to let go of current and, you know, like later and tomorrow and just kinda be in the moment. [Experimenter: Did it feel the same having the thoughts while you were floating versus out here?] I think like physically, I don't think my body was taking as much of a hit as it normally would; because with my stress and anxiety, my body is, you know—I exert the physical signs and symptoms. So I think in there, my mind might've been kinda stressing a little bit, but my body was definitely not taking a hit. It wasn't holding it.
—*Subject 8*

I mean I tried to think about my breathing more once I got used to, you know, floating. And so for a couple minutes, I mean, maybe five minutes of just sort of getting oriented to, you know, like actually floating, after that, a lot of it was trying to like let some of that tension out. Like I know, especially like in my shoulders, in my neck, in my jaw; 'cause I feel like those are kinda places that when I'm trying to go to sleep at night like that, I have to be cognizant of not, you know, to not like just let them remain tense. Especially like in my jaw because, you know, I think sometimes you just kinda sorta start breathing and you're listening to that. And for me, sometimes I don't think about how much my jaw's clenched. You know, I still also thought a lot about like current things like anxieties and worries, you know? So there were still some like rapid thinking like kinda running of thoughts. But they didn't, you know, they didn't seem as like alarming. They were still, you know, there in my mind. And I still thought about them, but it didn't seem quite as immediate as they normally do.
—*Subject 15*

I was able to kinda just zone out and just kind of relax. But occasionally, I would think about like, you know, 'What am I gonna do later today?' Or, you know, about maybe something I heard on the radio on the drive over. Just really benign stuff like that.
—*Subject 17*

That was basically it. Just kind of—I was like kind of playing with the water; like moving and stuff. And then it felt cool 'cause my hair was like moving effortlessly. But that's really all I remember. And the rest of it was pretty just calm and still. And I think I fell asleep towards the end.
—*Subject 18*

What did you think about, if anything, while you were floating?

[laughs] It sounds goofy… But, you know, we started out without music. And so initially, I was just kinda thinking about, you know, consciously trying to relax every single little muscle. And then, once I felt like I had kinda that piece of it down, went ahead and had the lights turned off. And what I found myself kind of [laughs] imagining, once I kinda got comfortable, was, you know, 'I wonder if this would be similar to what maybe astronauts feel.' Because of kinda that weightlessness and not really having an up or a down or, you know, you're just kind of suspended in space and, you know, if you could pass safely through space comfortably and safely, it might feel like this. You know, and if you were gonna do that, what would you see? You know? So it was kinda like imagining, you know, some of these photographs from the Cassini being sent back. And you know, it was just—I found that to be kind of neat; stimulating on a very low-key level. So you know, memories I had of maybe different times I've had massages done and, 'Okay, how does this compare to that?' And I found, oddly, that I would get distracted from thinking just by my own heartbeat and breathing. [laughs]
—Subject 19

It was thoughts—okay so at the beginning, before I got comfortable, it was just like, 'This is really nice. This is really interesting.' Kind of just fascinated by it all. But then, whenever I actually like got still—then I don't think I fell asleep. See, that's the thing is I don't think I fell asleep, but I might have. And the thoughts I was having turned into dreamlike thoughts. So I think I was having [laughs] dreamlike thoughts, but not actually asleep. That's how I would describe it.
—Subject 21

[no response]
—Subject 23

Well at first, I was trying to get my body to where I could relax. And then, my brain kept going to, 'What are you gonna cook for dinner?' And I'm like, 'No, don't think of that!' [laughs] Try to focus on like a what piece of paper. And I would just try to not think of anything. And then, I'd find myself jerking a muscle and waking up. Then I'd try to think of the beach. Something relaxing with water. Floating in the water. [Experimenter: So no negative thoughts during the float?] No.
—Subject 26

[laughs] As I put in the little form thing too, a lot of it was trying to get out of the thought patterns, which I normally have. Which you're trying to achieve stillness. So mostly, it was day-to-day stuff that kept popping into my head as far as, you know, work. It was like, 'Okay, I gotta do this tomorrow. And you know, over the weekend, I'm gonna do this.' And there's a whole bunch of, 'Okay, stop thinking! You're supposed to be still.' And then, also switching into, 'Okay concentrate on your breathing.' Basically reminding myself to try for the stillness or whatever. 'Okay, concentrate on your heartbeat instead. Okay, I wish I could turn off the music that's starting 'cause this really sucks.' [laughs] Just at the very start, because it did turn off; which was great, because I had splashed a little bit because I was looking for

a button to turn off the music. That's just me though!
—Subject 27

Initially, a lot of kinda the thoughts when you're falling asleep at night. Some recent bad news and kinda comparing it to slightly further back losses and some of those things. Some of the pragmatic task-oriented to-do list things. Then, eventually, able to kind of at least, one, be able to look at it a little bit differently, but then be able to kinda tuck it away. [Experimenter: How did you cope with these memories?] [sighs] I've kind of learned to accept; to just own them. They're not going to not be there. And just kind of like greet them and be like, "Yeah! There we are!' And move from it. Generally, probably not a whole lot of bringing it up. But unfortunately, the timing of bad news today meant that would be a day I would have to go through again.
—Subject 28

It's interesting. I mean it's hard to say 'cause that's my first time. I like the music on 'cause I feel like that kept me a little… silence tends to send your mind, you know, crazy. So I liked the music. But I don't know. I've never had it with music the whole time, so I really don't have anything to compare it to. I just think I did. [Experimenter: Any thoughts that you were thinking of? No negative thoughts or memories?] Huh-uh! Huh-uh! [Experimenter: So you weren't thinking about anything specific while you were floating?] Um random like, 'I'm itchy!' I thought the water was getting in my earplugs and it was [laughs] stuff like that!
—Subject 34

That I was just in peace and all my worries were gone and I didn't have to take medication to get there.
—AL454

Like in the beginning, I think I was thinking a lot about, 'Relax your body. Relax your body. Where are my hands going? Where are my hands going?' [laughs] So kinda thinking about like where my body position was. And then, after that, when I finally was able to like relax and just be, I like was thinking about like I have some work issues and so I was thinking about that. And then my wife. I was thinking about how much she would enjoy this. [laughs] Because she needs to de-stress. And then I went back to work. And then I was like doing breathing exercises about the short and then the extra long. And then thinking about just focusing on my breathing and just listening to it instead of anything else around me. And that really helped me like settle down. And then that's when I like was starting to like blank out my mind. And I feel like that was probably after a good fifteen… twenty minutes. But yeah, then I was good the rest of the time.
—Subject 45

I was thinking about what I have to do coming in the near future. But it was in a positive. So I seen it being successful. So it was mostly that. I didn't, too much, think about past memories or anything negative. So the whole experience was pretty pleasant and positive.
—Subject 46

What did you think about, if anything, while you were floating?

Well, lots of things. [laughs] I mean it kinda seemed like it kinda went in stages. [Experimenter: No negative thoughts?] No! And from that survey, it kinda surprised me that it had all these questions about worrisome and what have you, because I didn't have any anxiety at all while I was floating. Really is kinda introspective in a lot of senses. Just really—yeah, again with these stages, first of all, it is kind of distracting at first. Maybe 'cause of, you know, laying naked in a pool. So, you know, kind of getting past that. Then my mind would settle down and went to other thinks. And where introspective, I guess, is really kind of a lot of it was the—I thought about people that have passed and also about how to be—or I guess, maybe, the need to be more present in my current family's lives. Things like that. My mind wasn't really racing, but it'd go from a couple different topics, and then I just thought to myself, 'I need to try to just clear my mind," which was really hard to do. There's probably five or six times where I had a little jerked where—it wasn't any major—a lot of times, I'll have a big major jerk if I'm falling asleep on the couch or in a chair or something. These were just kind of mild ones. But they were enough to know that I was getting to the point of almost falling asleep.
—*Subject 48*

Just like random thoughts like before you fall asleep. Kind of thoughts like that, but not really—I was surprised about how clear like my mind was actually.
—*Subject 49*

I was just—I think I only thought of like for a few minutes—and that was at the beginning, when I got in—I was like, 'Okay.' So I got out my list. I was going through my list I was gonna take for my trip this weekend. Yeah and I'm like, 'I guess I got everything ready.' [Experimenter: No negative thoughts or memories?] No!
—*Subject 50*

Did anything surprise you during the float or happen unexpectedly?

No.
—Subject 33

No.
—Subject 20

Huh. No.
—Subject 35

I felt a lot more aware of my surroundings. So, every time that I was getting ready to hit the side, I felt like it coming on. I don't know if I was just realizing that the space was getting closer or what, but it seemed like every time that it would be like, 'Oh yeah, I'm getting ready to hit the side over here.' I mean I was—had no idea which way was what in the room, but…
—Subject 5

No. Not really.
—Subject 42

Mm-mm.
—Subject 24

When I first got in there, it was like—I don't know. I had like a little bit of anxiety 'cause I'm like, 'What if somebody come in here and drown me or something?' [laughs] But no. It wasn't a bad experience at all.
—Subject 9

That whole out-of-body thing. It was like feeling—it's so hard to explain. It's probably the first 5-10 minutes, there was like this emotional thing that almost made me wanna cry. It was like I was just—all of a sudden just aware of this complete—I don't know if it was like an emotional release that—'cause I know you're a doctor. The closest thing I can explain it to, is in some past relationships, sexually, I felt that when I've had an orgasm and I've cried. Because I—and I used to do that a lot—all the time. Because I've gotten older and things don't happen as easily. I haven't done that in a long time. And when I got in there, and I just relaxed, that was like the closest—not in a sexual way, but in a very emotional way. And I didn't cry, but I came really close to it. And I couldn't explain why. That was the—weird. And I'm, you know, so it's probably the first time I've really relaxed in a long time. [laughs] Yeah, it was really weird. But that whole out-of-body experience was like 'Woah, is this what it's gonna feel like after you die?' You know? But it was a pleasant thing. It was very pleasant. So it was kinda cool.
—Subject 16

[no response]
—Subject 30

Nope.
—Subject 40

No. No. The colors that I saw, I was happy to see them because sometimes I see those colors when I meditate.
—Subject 2

[no response]
—Subject 38

Nothing. Nothing, really. Yeah.
—Subject 10

No.
—Subject 43

Did anything surprise you during the float or happen unexpectedly?

Um, I don't know. I mean it did—I mean, it was a different sensation just being there. Like, just still. So, I guess that was really—it was unique in that sense.
Um, I think it kinda surprised me how—not how quickly—but how—just kinda how it just happened. It was kinda just you're in like one state and then it just kinda transitioned; kinda something like that [laughs].
—*Subject 4*

Mm-hm. Well, kinda like close to the end, I felt like was falling. I guess I was waking up.
—*Subject 14*

No.
—*Subject 37*

No. But because I've read up on it a year ago. When they first became popular or when Tulsa got its first, I read up on it and why it's used and why people like it. So I knew what should have happened,
and I think if I didn't know what was going to happen, I would've been more freaked out.
—*Subject 22*

No.
—*Subject 6*

I really thought, um, I was going to go into this deep relaxing state and just letting go; I never thought that it would have the opposite effect and give me a little bit more energy and clarity. That's what I was really surprised about.
—*Subject 11*

No. Mm-mm.
—*Subject 31*

[no response]
—*Subject 29*

Well, like I said, just that feeling like all my muscles are gonna like… That's almost—when I first got in, it felt like there was—'Oh my gosh, my body's gonna cramp up!' And which it didn't! But I've had cramps before and I hate that feeling! [laughs] So it was a little scary, but I kinda had to pay attention because, you know, like you talked about the back and neck tension. And I was paying attention to that, but I'm noticing like my toes, for instance. Like my toes are gonna cramp up! And I had to like really pay attention to them. And just relax them. Like everything I guess was tense, so I had to like relax everything on purpose.
—*Subject 36*

No, not really. I knew because I love water and, you know, if I had it my way, I would have one of—I would love to have one of those in my house. I mean, I'm just, you know, I'm a bath person anyway, and I like to relax in water. And so, you know, that was just like… I didn't—you know, you mentioned that you might feel a little of the, you know, of the build-up, but you know, that— I'm kinda used to the, you know, going to the beach, or used to feeling, you know, so that was not a sensation that alarmed me at all.
—*Subject 1*

Did anything surprise you during the float or happen unexpectedly?

Not really, no. The water was just almost perfect.
—*Subject 44*

Nope. And the water and the towel next to the float pool itself was a great idea, 'cause I definitely utilized that. [laughs] You don't even think about it. You're waving your hand and you're like, 'Okay, it's in my face now.' [laughs] So, that was definitely handy.
—*Subject 13*

No. I mean the—like I don't know that I actually fell asleep, but I was really—I was trying to focus on staying awake so I could kind of, you know, live the sensation I guess. And I know it, again, was when I coughed, it was—you know, I don't know if I woke up or if I just came out of kinda that, you know, being able to not think about anything—sensations. So and that was really about it. I mean I was surprised that I didn't fall asleep faster 'cause, you know, I have to wear a CPAP when I sleep. And I have a really hard—you know, usually I sit down in a chair for 5 minutes and I'm out. So I thought, you know, I'll just be sitting here and I'll fall asleep as soon as soon as I close my eyes.
—*Subject 12*

No.
—*Subject 47*

[begins crying] Excuse me. These aren't like sad tears. Like I'm very emotional in the opposite way; of appreciation. And it was for me, for my calming, my experience, for my healing. Like thinking about walking out of that door, I was like, 'Wow, I'm gonna have to go back in the world.' And, you know, a lot of the normal things that I have to experience. And I did feel a sense of dread. But it did turn—make me go right back into more of a positive of… that I got that experience. That I know it exists. That there is something. Does that make sense? [Experimenter: How would you separate those (Epsom salt baths at home versus floating) in terms of differences?] I mean I tried real hard to just be in the moment and be there and make it for myself, but life isn't gonna stop. And you're still having to do a duty and a process and know what you need to do before and after. And animals. And phones. And it's completely different. Like you shut that door and that's a world. I guess I wasn't expecting that. [Experimenter: How about the pain?] That is amazing too, because I mean I carry it all the time. And there—you're right about having to find it and like let go. And I had to. The mindfulness did help because I would think I was completely relaxed, and I would start, 'Okay, how does this feel? How does that feel?' And I was like, 'Okay, I can even relax more! That is still tense. And I can let that go.' And I did a lot of those like when you were young, you would really just stretch your whole body out and you could really feel how tense that was. I did a lot of that! And it felt really, really good! And I did do the I'm-just-relaxing-with-my-arms-behind-my-head. So the stillness was still there. But I really did try to find ways and other ways of what it felt like! And what was relaxing. And so yeah, like even breathing and thinking about your breathing. Like just think about it, remind yourself of it, because you get—you still—even when

you're in a relaxed state, you can forget and it helps to remind yourself and think about it; 'Well, how is your neck feeling? Well, how are your toes feeling?' You know? [Experimenter: Did the pain actually go away or was it always still present?] No, it went away! Like the tension was—it was a different tightness. I can't explain it very well, but it was not there because something else was holding it. There wasn't a weight… pulling you down. 'Cause I even could feel my spot. Like the main spot that I always feel when the injury happened and everything, and it still wasn't a pain spot even though I could still feel—and I know that's where it was and that is the issue. [Experimenter: How is that different or the same as when you take morphine?] I never go away from the pain with morphine. It's just more of a try-to-get-through… and you're still gonna have to do life things. You're gonna be painful, so you put a little bit of a coating on it. It's always completely still there. I mean I go through all kinds of stuff. I do a process of things that will not ever alleviate anything.
—*Subject 3*

[no response]
—*Subject 7*

Well, I know I popped my knuckle and it was a little louder than—[laughs] but I knew it was me. And I think like 5 or 10 minutes later, like I heard something and it sounded like a pop again. But I was like, 'I don't think that was me,' you know? But I went ahead and shut the light, you know, and thinking my eyes were going to adjust, but it was so dark in there, my eyes did not adjust. But I mean I knew at any time, I could, you know, get the lights back on. But you know, I just was kind of, in a way, trying to fight these normal anxieties. You know, knowing—I just kept—which is a little different for me 'cause normally whenever my anxiety gets going or I'm stressed, I get stuck there and I don't realize it. But like I knew it was a safe environment. And I knew, you know, even though my mind was trying to play tricks on me and run with, 'What was that noise?!' [laughs] Like, 'Is somebody creeping around in here?' You know? Like I, you know, just kept kinda like feeling my body like, 'Oh, this feels so good and this is a safe environment! Just kick it out!' Like in a way, I knew that it was a safe environment. And so if I heard something, it would kinda freak me out a little bit, you know, at first. Then I was like, 'I don't care.' [laughs] 'Whatever!' Which is, like I said, different; 'cause normally I'll get stuck there and I can't pull myself out.
—*Subject 8*

No. You know, I think I was just, again, like I was wanting to be aware of like when the music stopped and when the lights were gonna come on. But other than that, I mean there wasn't anything that was like surprising. I think, you know, like I said, it's interesting getting used to—'cause I mean, you know, you're gonna float, but I think like just adjusting to where you're thinking like, 'Okay, it's like alright to, you know, let go of that tension in your neck,' or, 'It's okay to like let go of your arms and like, you know, whatever you have going on in your shoulders because you can put them however you want.' You know? Literally, you don't have to be worried about, 'What if this…' You know, 'What if my arm falls asleep?' 'Cause I feel

like sometimes I do, then, when I actually sleep. 'Cause I might sleep on it or in a funny way. So maybe it's like surprising getting used to how much you can let go of those like normal sensations and normal—you know, 'cause, for me, I think sometimes you wanna feel like you're kind of like in control of something. And I think sometimes like that wanting to control something means that there's like a lot of tension put there; whether it's knowing like consciously or unconsciously. So I think it was surprising how much you can consciously kind of just, you know, say, 'Okay, I can feel maybe some tension like in my back or in my neck or in my shoulders.' And I can kinda just like let it be. There's, you know, the water, you know, is supporting me and I can actually—you know, I can feel the tension and I can start to like kinda just let it go and relax.
—*Subject 15*

Not really. Just, you know, occasionally I might hear a sound; like a piper or something and I'd think like, 'Oh, what was that?' And I think that [the amplification in room] just combined with my general like getting used to the experience and everything. I maybe like would pay attention briefly more to like a click or a pop than I normally would.
—*Subject 17*

Besides floating. [laughs] No, not really, I don't think. Now that I like look back, it feels like it went by so fast, but [laughs] I don't remember each.
—*Subject 18*

I don't even know how to describe it. I felt like my back, like upper back between my shoulders—I almost felt like they were more tense coming out than they were goin in. And I was taken by surprise at just how shaky I was, trying to get out. And so that was surprising. I mean I remembered what you said about moving at a slow speed. And that's great advice because if I had tried [laughs] to go any faster, it would've been disastrous. And so yeah, it took my eyes a few seconds longer, I think, to kind of focus 'cause suddenly we're having to key in to where we are in space and make sure we're upright and everything's working and that we're ready to stand up. So that was surprising. I was very surprised just how floppy they (muscles) felt.
—*Subject 19*

Yeah! The lack of physical sensation, at some point was. Whenever I realized that that had happened, that was very strange! That was really strange! It still is kinda blowing my mind a little bit.
—*Subject 21*

No, other than falling asleep. 'Cause I've never done that before. And probably the—well I may have fallen asleep twice because I do remember sort of twitching just a little bit, which I've not done before.
—*Subject 23*

No. It's just kinda funny that you bump into the sides. And I had wondered where my body was in the room, but nothing unusual.
—*Subject 26*

So, well, whenever the fish kept on pecking at my feet. I didn't know you guys had fish in the pool, but… okay I'm totally kidding!

Did anything surprise you during the float or happen unexpectedly?

Totally kidding! [laughs] No, nothing really! I put in the, 'totally kidding,' so they would know that I wasn't actually… Yeah, so but some fish would be nice! They have those fish in Mexico that eat the skin off your feet! And so yeah, throw a couple of fish in there! Except for it being saltier than the Dead Sea. No, nothing really unexpected! And again, I couldn't slide right into it, you know? The same as before, I was a little bit anxious right at the start. Just kinda getting comfortable with myself and then—the neck thing. So kinda making sure that my neck was loosened up or whatever. But nothing, yeah, crazy or unexpected!
—Subject 27

Well, I suppose nodding off—or if I did. Whatever I did that kinda jolted me. Beyond that, no, I was very fascinated with the crystallized sensation of the salt on me; but managed to like not pay attention to myself. My toes got cold. Like apparently like the tips of my toes were sticking up. And I believed—every once in a while, I'd tuck them back down under the water. And I think that the air temperature was perfectly fine. I just think that, maybe, my toes weren't getting enough circulation in there and they're like, 'I'm cold.'
—Subject 28

Probably the temperature! I was a little leery about being cold or not, but it was pretty perfect!
—Subject 34

Just to float, float above water.
—Subject 39

[no response]
—Subject 45

Well one thing; my body felt kinda heavy. Like especially I noticed when I was getting out of the pool. That's one thing that kinda surprised me. I didn't expect that. I feel very relaxed right now. [laughs] Almost like I could go to sleep. Very peaceful. [laughs]
—Subject 46

Well, the only kinda weird thing was my shoulder started to hurt a little, you know, after I was in there for quite a long time, because of the old injury I have. But at night, when I'm sleeping, I put this arm up above me like this, the pain goes away. So I put both arms up, and the pain completely went away that way. But it made me feel a little more vulnerable, I guess, which is kind of a weird feeling. The pain completely went away to where I could get back in that relaxed, almost out-of-it stage again.
—Subject 48

No.
—Subject 49

Yes! [laughs] Well, I was telling her like when I barely like put my first foot in, it was just floating already, and I was like, 'What?!' I was trying to put it down, but it wouldn't stay down 'cause it was floating. [laughs] And then like, 'Oh, that's what it means to have a different type of salt in it.' And once I started to go in, it was kind of like I already felt like the gravity was not as heavy.
—Subject 50

Did you learn anything about yourself during this experience?

I had a hard time being still, but I think I kind of knew that anyway. The only thing I had any issue was that there was a difference in the, for me, a difference in the air temperature, and it only affected my toes. So every once in a while, I'd dip my toes in, which of course—then a few seconds later, they would feel cold again. I don't think there was really that much difference in the air, but for me. Yeah, and the only other thing is—and it could be because I have a colder—
I think I have a cold, but my breathing, it just felt like the being in water, it still felt like everything was dry. Okay, it wasn't just my imagination [laughs]. It was very pleasant. I'll give it that. I mean I don't know what it's supposed to accomplish altogether, but it was a very pleasant experience.
—Subject 33

I didn't think I would get as relaxed as I was, but I was pretty relaxed. Yeah. I feel like I could sleep really easily right now.
—Subject 20

Just that I need to like stop and calm down; I mean [laughs] basically.
—Subject 35

Don't know if I learned anything about myself. It did sort of reiterate the need of having a meditation or something like that daily really just does affect everything throughout the rest of my day. So sometimes it's easy for me to get off that. And it's nice having something that's like, 'Oh no that's—you need to do that. That's… that's good for your mind.'
—Subject 5

I need to buy one of those.
—Subject 42

That I love to take a long hot shower. And I'd be one of those people who probably needed to say to, 'Don't take too long because you gotta get it ready for somebody else.' I don't know that there is anything negative to say about it other than the fact that I could've stayed in there a whole lot longer had I not done what I did with my arm.
—Subject 24

I wouldn't say 'learned something,' but I guess I learned something. That whatever that is in the water, that type of setting can help me not stress so hard and just like calm myself down. I don't know. Take the edge off of the thoughts. That just helped me not like overreact to my thoughts and all of that.
—Subject 9

I learned a couple things. One, I learned that I was able to go like somewhere else in my mind, which I've not. Different from that beginning feeling, when I kinda had that out-of-body experience thing. I don't know that I've ever done that before. And I didn't really know that—cause when I've heard people say that stuff, I'm kinda like, 'Psh!' You know, 'Whatever!' [laughs] That was really—it was really cool. I learned, again, that it's extremely hard for me to turn my brain off.
—Subject 16

Yes! Yes! I learned that through paying attention to my sensations—my body sensations—or maybe just being more aware of my breath even at times can help me relax more. I always tell my students 'deep

breathing. And think of positive thoughts.' And it's very easy to say all of that and try to help somebody get there. Thanks for the experience.
—*Subject 30*

I think I'm addicted to outside stimuli. [laughs] I really do. Not so much like a phone, but like a book or something to focus on. Yeah. I don't think I know how to just be anymore.
—*Subject 40*

Yes. That I could sleep with a night light on. I don't like being in the dark. It typically makes me feel very anxious, and so that is something that I wanted to do—I wanted to have the lights off, see how I'd do. I was able to let go. I was able to not be afraid at all in there and feel safe. So that, to me, it's not something that I learned about myself, but it feels like an accomplishment. It was wonderful. Nice Christmas present! [laughs]
—*Subject 2*

I wanna let go, but it's kinda hard for me. All that tension I felt—I was trying really hard. And then I thought 'You know, maybe I should just stop trying and just let it go.' Because I'm a people pleaser.
—*Subject 38*

I don't know. I don't think so, but I think I will come back after a few times.
—*Subject 10*

Yeah. I learned that, right now, I'm not capable of relaxing and clearing my mind for a full 60 minutes. I'm just keeping it honest.
—*Subject 43*

I kinda did. I felt more present, like I said earlier. I think it kind of feels like it put things in perspective, 'cause the only time you really think about like actually being here—just like, I don't know, being present or whatever. You don't really think about it until you're like basically gone like in there. [laughs]
—*Subject 4*

If I just take the time and kinda have a moment to myself to where I don't think about anything, I'll feel a lot better. Instead of thinking of all the negativity things; what I should have done. You know? Things like that.
—*Subject 14*

I should do this more often. Cause I had some anxieties about my week last week; when I came outta there, it just all went away. [laughs]
—*Subject 37*

Yes. I wasn't expecting to be anxious at all. Like I was actually really excited, because I'd read up on it, and those things are ninety-nine dollars for three sessions. I didn't realize I was that anxious of a person; or so easily terrified by my surroundings.
—*Subject 22*

No I don't think so.
—*Subject 6*

That a part of me is still in there. That I always feel so disconnected by my circumstances because they're so abnormal. And there's always this sense of dread that it's like, 'This is it.' Then, every now and

then, you have this opportunity to experience a part of yourself and it's reassuring to know that it's still there. It's not destroyed by brain damage or anxiety or just living with PTSD. That was a really good opportunity to alleviate so many different things and just get a nice quick opportunity to get closer to yourself. To know that you're still there.
—Subject 11

I guess. I mean, like anyone else, you have things on your mind. But I guess you can kind of put that stuff on the back burner. You can kinda put it away, once you're in the right setting. And you know, things may not seem as bad as they are, once you kinda give yourself a break and kinda be to yourself.
—Subject 31

[no response]
—Subject 29
Yeah! It's really weird. I was filling out the questionnaires, in the before and after. And it's hard with the questionnaires, because the words mean something totally different to everybody. I felt happier and more energized and everything; and I'm feeling less strong and less determined. I would've thought that those are positive things. And then I'm like maybe that wasn't what I thought it was.
—Subject 36

I think I can really mention from a small town, closed-mind, everybody, you know, kind of having the same way of thinking; the whole new age, I could see where that would be something I could get into. The mind, the body, the soul. I really felt like that could be something I could look into further.
—Subject 1

Mm-mm no.
—Subject 44

I don't think I learned anything new about myself, but I did kinda confirm things that I was thinking I knew. I know that I normally think all the time and that I rarely stop thinking. Even whenever I try to relax, it's harder sometimes. But I think it just kinda helped me almost come to terms with how I function. It kinda helped me figure out that's okay.
—Subject 13

I don't know. I guess just that kinda knowing that everything can be shut off.
—Subject 12

Yeah, I guess I did. I guess it was easier than I thought to not let negative thoughts get into my head because I thought for sure that, you know, that's the first thing that would happen. But it didn't. I guess maybe because I knew beforehand that this was gonna have to do with not having negative thoughts during this. So maybe I subconsciously—I don't know. [laughs] I don't know.
—Subject 47

I have a lot of negative thoughts about myself and stuck, hopeless feelings of life and myself. And I also know there's a lot of beauty, but it has just been stuffed. So I felt that grow. I felt that I was in there. Still strong. Still could fight through it. That there is a little bit of hope. And of course, I always—the mantra is to keep going, but there was more, I guess, sort of faith with that. Like you could touch it if you… I can't explain it. It was touchable. It was there.

Did you learn anything about yourself during this experience?

Things could be better. There was a reconnect in so many areas! And so yeah, my body and soul kind of like, 'Hello.' Sort of like the childhood innocence of—because that sensation is—I know it sounds weird—but motherly. Do you know what I mean? Like I even wrote that in one of the things. Like a Mother Earth kind of thing. And I really love this mural because of that! That's exactly the feeling without saying anything. And the reflection. It should be called, 'Reflection.'
—*Subject 3*

Nothing that I didn't already know really. And just I need to calm down. [laughs] And I need my thoughts to stop, which they don't. Ever. And I am very restless. I'm wondering if it's like me or if it's something that can be helped. But that's why I'm going to therapy. So I'm kind of debating whether I should change my prescription 'cause it's not changing much of anything. I don't feel like it is.
—*Subject 7*

You know, what I was talking about, you know, with that little anxiety that kinda popped up, you know, I would like to somehow figure out, like how I defeated that in there and twist it and somehow try to figure out how to apply it to, you know, my life outside of here, you know? 'Cause like I said, normally I would get stuck in it. And sometimes when I realize that I'm, you know, in it, you know, in that anxiety, where everything, you know—and I don't know. I would like to figure out how to—I mean as small as it might sound, like to me, that's a big thing. 'Cause I heard a noise and I'm like, [laughs] 'Is somebody gonna come push me under the water? Drown me?' Like, you know, I mean these are real things that go on in my head. You were talking about a little… you know, and that wasn't too bad. Like in a way, think it's just because, you know, people don't know how to relax. And then, you know, this part is obviously lighter weight than your head, and so you're like… [laughs] you know? But I did notice—and, you know, I think that's just my body storing, you know, those are the places that it stores that stress that I maybe don't even realize. Like I know it's normally here, but you know, after some time I felt, you know, my joints. Like I felt a lot here and then my knees a little bit, you know? And I'm just thinking that that is just, you know… So you know, outside of here, that's some stuff that I kind of wanna focus on too, is, you know, how to try to alleviate some of the physical symptoms. And I definitely have a lot more energy now. [laughs] Because I think like I don't know how everybody else is, as normal people, but with me and my stress and my mind going constantly, like it just takes away my energy. Like I know that that is why I'm just physically just drained all the time. And mentally! You know? It's because my brain. And I can't just slow it down. So I kinda feel like I just slept for three days. [laughs] You know?
—*Subject 8*

Well, I don't know. I feel like there was a couple different ways that I like kinda had my arms just positioned through the time. I felt like there was probably more ways I found to where I could just kinda like let them be and not like feel tense or like, 'It's gotta be here. It's gotta be there.' Probably how much

tension I like put in my jaw, because again, like I was listening to breathing. And as I was doing that—like I know that I tense my jaw, but I didn't know how often. 'Cause I mean I'm in here and I'm thinking like, 'Okay, this is, you know, like I don't have as, you know, as much tension in my neck as I normally do. Or in my shoulders or my arms.' And then I'm kind of like trying to just feel the rest of my body and where you'd feel like, 'Why would there be tension when you can literally just let it like support you?' And I could feel even more like just that, for some reason, wanting to like keep my jaw tight even though, clearly, you don't have to and there's no need at all.
—Subject 15

I felt like I could see something like this like being beneficial to me. It felt very soothing, kinda like meditating. But I really tried to—I don't know if focus, but really try to tune in like the weightlessness and the not feeling a whole lot. And just kind of seeing what that was like. And it did feel good. So I felt like, you know, if I was to do—I could see reaching out or seeking something like this out again.
—Subject 17

No, I didn't really. I don't think I really learned anything. I was just trying to stay like quiet and just like enjoy it.
—Subject 18

I did. After, you know, [laughs] as I was in there getting dressed, I was thinking, 'It's interesting that my mind is always looking for something to focus on.' That it's that driven to—it has to be doing something right now. All the time. And I don't know if that is a consequence of my background or if that is a consequence of like a heightened world technology that we encounter every day because we're so accessible all the time. But yet, we don't develop this type of alarm fatigue that a lot of clinicians develop when they hear the same alarms go off over and over. So it was just kind of food for thought. I was like, 'I didn't realize my mind's always searching for something to focus on and gets very restless like that when there's nothing to focus on.
—Subject 19

Probably! I don't know what yet, but [laughs] probably. Yeah, it's different! And I would say that I've learned that, if anything, right off the bat, that there is some obvious benefit for me to find a way to remove myself from the world round me. And so probably I need more of that!
—Subject 21

[no response]
—Subject 23

I learned that I could put my head halfway under water and trust that the earplugs were gonna work; 'cause I usually don't ever put my head under water 'cause water usually goes right in my ears. And that I can actually feel weightless without the pressures of my joints.
—Subject 26

No, not really.
—Subject 27

Did you learn anything about yourself during this experience?

Not necessarily. I was actually a little bit surprised at how easily I was able to re- relax because, as a person that normally relaxing is not the easiest thing to do, I had a feeling it was gonna be like, 'Oh heavens, let's start all over.' And normally, if I'm in like a dark place, I feel somewhat claustrophobic. And I suppose, since I had my arms out kind of like this, I didn't feel that. It wasn't like the MRI sensory deprivation, but it might as well have been. I didn't really need to move! I guess the knowledge that I had the ability was perfectly fine.
—*Subject 28*

Yes. I can't stay still for very long. [laughs] And an hour float seems to be a long time.
—*Subject 34*

Yes. I learned that medication isn't always the answer. That out there, I could search for, you know, other things to do like relaxing. Taking a hot bath might ease it before I take medicine.
—*Subject 39*

Yes. I need to figure out how to relax! [laughs] And just maybe I need to make an actual effort to do it; instead of being like, 'Oh yeah, I need to chill out,' and think it, and keep going. I actually need to do it because I feel—the way I feel now, if I could feel like this at other parts during my week, I feel like I would get more stuff done and more accomplished and I would have that clarity of being able to do what I need to do instead of having like the anxiety and worrisome. And so I feel like I need to learn how to relax. [laughs]
—*AQ987*

I did! One thing that I really want to change about myself is how I focus. 'Cause I don't think I'm very good at focusing. And also I feel like I can use this type of technique to help even manage my emotions to kinda focus and be more logical because I tend to be more emotional about things. And it helped me to focus and to think about like things I need to do in my future without like the emotion attached to it.
—*Subject 46*

Well, I mean I think just kinda like what I stated as far as, probably, the need to be more present around other family members.
—*Subject 48*

I just—I don't know if I learned anything about myself, but I was just very relaxed. Like honestly, I would compare it to getting out of like a yoga practice and feeling really refreshed. I feel more refreshed than I did waking up this morning. Like I feel very energized and like refreshed and just, yeah, I feel really good honestly.
—*Subject 49*

In that type of water, my neck doesn't hurt when I'm floating. [laughs] And I think that's it! Like that's just my body; how it gets there. Like I was telling her, I love floating already. Like in a regular pool with my daughter. And I struggle with balancing my body and breathing and then exhaling and inhaling in order to keep the body stay stable. But if you don't do that, your neck would go down. But in this water, it's like you're breathing normal and you're already floating straight. So your neck is not tense! So my body just relaxed.
—*Subject 50*
