# Supplementary material for: Examining the short-term anxiolytic and antidepressant effect of Floatation-REST
Source: PLoS One. 2018 Feb 2;13(2):e0190292. doi: 10.1371/journal.pone.0190292 (PMC5796691; doi:10.1371/journal.pone.0190292)
Supplement: S1 Protocol — (PDF) [file pone.0190292.s009.pdf]

Title of Project: Examining the effects of reduced environmental stimulation

Principal Investigator: Justin Feinstein, Ph.D.

Address:
Laureate Institute for Brain Research
6655 S Yale Ave
Tulsa, OK 74136-3326

Phone:
918-502-5169

Fax:
918-502-5135

Email:
jfeinstein@laureateinstitute.org

Sponsors:
Laureate Institute for Brain Research
http://www.laureateinstitute.org

A. Purpose of the study

The human brain is constantly bombarded with sensory information from the external world. This series of studies aim to explore the effects of reducing environmental stimulation using specially designed floatation pools that minimize visual, auditory, tactile, proprioceptive, and thermal input to the brain. Previous research has shown that "floating" in this unique setting can significantly reduce levels of anxiety, stress, blood pressure, and cortisol, while significantly increasing levels of both subjective and physiological forms of relaxation. Much of this past research contained various methodological weaknesses, including small sample sizes, lack of a control group, and no longitudinal follow-up. Moreover, very little is known about the potential benefits of floating in clinical populations, and essentially nothing is known about the effects of floating on the brain. The studies proposed in this protocol aim to further explore floating's potentially salubrious effect on the autonomic nervous system, while beginning to investigate its largely unknown effect on the central nervous system. We have attempted to improve upon the weaknesses of past research by using larger sample sizes, a control group, and a longitudinal design. The current project is focused on documenting the subjective, behavioral, physiological and neural effects of floating in healthy and anxious populations. The subjective effects of floating will be examined using self-report measures and the experience sampling method. The behavioral effects of floating will be examined using measures of interoceptive awareness and distress tolerance. The physiological effects of floating will be examined using waterproof and wireless tracking of blood pressure, heart rate, respiration, and movement, in addition to collecting measures of cortisol and magnesium. A portable electroencephalography (EEG) system will be used to measure sleep during the nights before and after a float experience. The neural effects of floating will be examined using waterproof and wireless EEG collected during the float experience, as well as using functional magnetic resonance imaging (fMRI) collected before and immediately after floating. Using a longitudinal within-subject design, we have a unique opportunity to assess not only functional brain changes, but also structural brain changes induced by repeated exposures to floating. An active control condition aims to control for the effects of simple relaxation by collecting all of the same measures while participants lay supine in a zero-gravity chair situated in a quiet, dimly lit room. This program of research constitutes the first systematic investigation of floating on the body and the brain, and the findings have the potential to illuminate the physiological and neural correlates of the deep relaxation induced by the floating experience.

Specific Aims:

- **#1:** Evaluate the subjective effects of floating
- **#2:** Evaluate the behavioral effects of floating
- **#3:** Evaluate the physiological effects of floating
- **#4:** Evaluate the neural effects of floating

B. Background and Significance

The history of floating dates back to the 1950's when Drs. Jay Shurley and John Lilly at the National Institute of Mental Health became interested in understanding how the human brain would react if it entered an environment largely devoid of external sensory input (Lilly & Shurley, 1961; Shurley, 1960). It was discovered that rather than falling asleep or losing consciousness, the participant maintained full awareness. The initial version of these float tanks had the participant immersed vertically in water while wearing an opaque breathing helmet. Due to the confined nature of the helmet, very few individuals participated in these early experiments. In the 1970's, Glenn Perry (in collaboration with John Lilly) invented a horizontal version of the float tank that removed the need to wear a helmet (Lilly, 1977). This newer version of the float tank (which is the same version being used in modern times) has

participants lay horizontally in a shallow tank of water that is saturated with high concentrations of Epsom salts, thus allowing participants to float effortlessly with their eyes, nose, and mouth above the water surface. While this change in design exposed floating to a much wider audience, many individuals still found the tanks to be too confining and claustrophobic in nature. Bolstered by the creation of more spacious float tanks, the past 5 years has witnessed a widespread resurgence in floating. So-called "float centers" have started to open around the world, where individuals will pay an hourly fee to relax inside a float tank. There are currently over 200 float centers in America and nearly 600 float centers worldwide (*cf.* http://floatationlocations.com/).

Despite this resurgence, very little research has systematically investigated the effects of floating. The majority of floatation-based research occurred in the 1980's and 1990's, and many of these earlier studies suffered from various methodological weaknesses (e.g., small sample sizes, lack of a control group, and no longitudinal design). Nevertheless, there have been several important findings. The most replicated result thus far has been the significant reduction in levels of subjective stress and anxiety as measured from pre- to post-float (Barabasz et al., 1993; Bood et al., 2006; Forgays & Belinson, 1986; Kjellgren et al., 2001; Koula et al., 1990; O'Leary & Heilbronner, 1990; Suedfeld & Eich, 1995; Suedfeld et al., 1983; Turner et al., 1993). Consistent with these data, floating has been shown to significantly decrease blood pressure (Fine & Turner, 1982; Jacobs et al., 1984; O'Leary & Heilbronner, 1990; Turner et al., 1993), heart rate (Forgays & Belinson, 1986; O'Leary & Heilbronner, 1990), as well as cortisol and other stress hormones (Turner & Fine, 1983; Turner & Fine, 1991; Turner et al., 1993; but see Schulz & Kaspar, 1994). A recent meta-analysis of 27 float studies found a large overall effect size for the amount of stress reduction (Dierendonck & Nijenhuis, 2005), with the vast majority of research focused on healthy populations. Clinical studies thus far have shown beneficial effects of floating in patients with hypertension (Fine & Turner, 1982), as well as chronic pain and stress (Bood et al., 2006; Bood et al., 2007; Koula et al., 1990; Kjellgren et al., 2001). Thus, floating shows great promise as a tool for reducing stress, but much more research needs to be conducted in clinical populations, especially psychiatric patients who suffer from conditions related to heightened levels of anxiety.

The relaxation response induced by the floating experience is very different from the unpleasant and often anxious state induced by other forms of sensory deprivation (Zubek, 1969; Suedfeld, 1989). This important difference is further emphasized by the conspicuous lack of negative adverse side effects associated with floating; nearly all float studies to date (see previous paragraph) have reported that participants find the overall experience to be pleasurable and relaxing. Distorted time perception, out-of-body experiences, and occasional visual and auditory hallucinations (typically of the hypnagogic variety) have been reported, but such effects seem to be entirely confined to the float experience and do not appear to linger after the experience is over or necessarily cause any distress (Norlander et al., 2000). Anecdotal reports further suggest that rather than depriving the senses, floating may actually enhance one's internal awareness of sensations from the viscera, although this has never been systematically investigated.

Several studies have examined the long-term effects of floating. For example, in some of Jay Shurley's original float research, he found individuals who were able to float for up to 12 hours (Shurley, 1966). Bood et al. (2007) had a group of patients diagnosed with stress-related pain float 1-2 times a week for up to 33 float sessions, with no reported adverse effects. Another study had participants float for 2.5 hours every week for 6 months and found no adverse effects and sustained reductions in anxiety throughout the 6-month time period (Pudvah & Rzewnicki, 1990). In conclusion, over a half-century of research has demonstrated that floating is a safe noninvasive intervention capable of reducing stress and anxiety.

C. Preliminary Studies & Description of Laboratory

The main investigators on this protocol (Justin Feinstein, PhD; Sahib Khalsa, MD, PhD; Kyle Simmons, PhD; Jerzy Bodurka, PhD; and Martin Paulus, MD) are all based at LIBR. Two of the investigators (Sahib Khalsa, MD, PhD & Martin Paulus, MD) are board-certified licensed psychiatrists who can assist in the event that any adverse psychological effects should arise during the course of the study and the primary investigator (Justin Feinstein, PhD) is a clinical neuropsychologist who has been extensively trained on treating patients in acute states of distress. This protocol represents our first effort to conduct float-related research. Nevertheless, our group has published numerous studies using other methodological tools listed in the current IRB protocol, including fMRI, EEG, and other psychophysiological measures.

In 2014, LIBR built the Float Clinic and Research Center (FCRC), a laboratory directed by Dr. Justin Feinstein that contains two custom-designed fiberglass floatation pools, including an open pool and an enclosed pool (Figure 1). Both float pools were manufactured in the United Kingdom by Colin Stanwell-Smith, an experienced engineer who is the founder and owner of Floataway (http://www.floataway.com/), a company that has been designing float pools since 1999. The only structural difference between the two float pools is that the enclosed pool has blue acrylic walls and a domed ceiling that is ~7.5 feet tall. Overall, the experience in both float pools is essentially the same when the lights are out. The only exception is that the enclosed pool can calibrate the air temperature and humidity a little more precisely than the open float pool, whereas the open float pool tends to be more appealing to individuals with claustrophobia.

Figure 1. Pictures of the open (left) and enclosed (right) floatation pools at LIBR's FCRC.

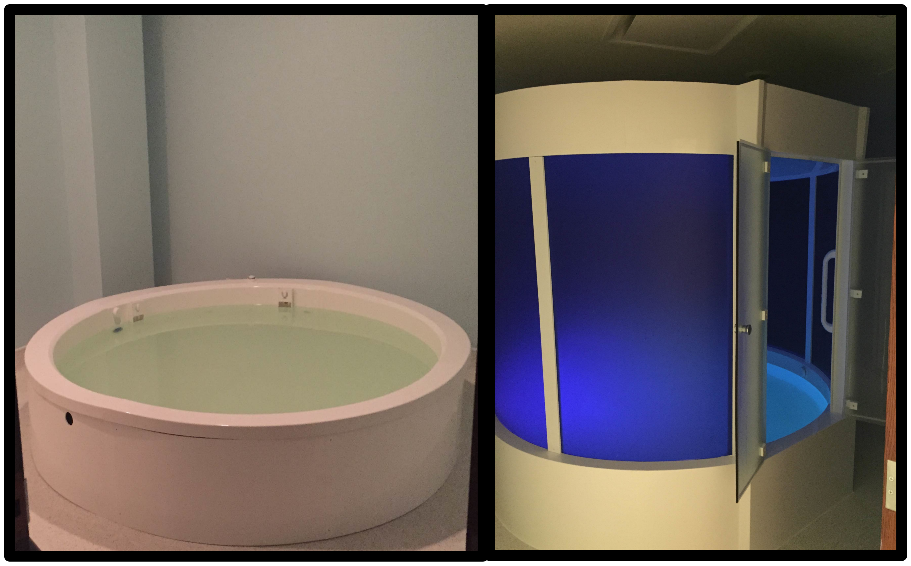

Both circular pools are the same size (8 feet in diameter) and contain 11 inches of reverse osmosis water (330 gallons) mixed with ~2000 pounds of USP grade Epsom salt (magnesium sulphate), creating a salt water solution with a specific gravity of ~1.3, which is denser than the Dead Sea. Beyond the natural disinfection created by such a high concentration of salt, the water in each pool is circulated through a powerful disinfection system. The disinfection system includes a 10-micron filter to trap any loose hair or skin particles, high-powered ultraviolet (UV) disinfection delivered via a series of 6 UV lights, and 35%

food-grade Hydrogen Peroxide (H_2O_2) maintained at ~50ppm. The combination of UV with H_2O_2 creates a free hydroxyl radical that is nearly twice as powerful as chlorine at oxidizing and destroying microorganisms, and also has the added advantage of producing no dangerous by-products during the oxidative process (Crandall, 1986). Between every floater, the water is circulated through this disinfection system a minimum of 3 full turnovers.

Both float rooms create an environment with minimal visual, auditory, tactile, proprioceptive, and thermal input to the brain. The float rooms are both lightproof, and thus completely dark when the entry door is sealed and the lights are turned off, reducing all visual input to the brain. Each float room was constructed with thick soundproof walls, restricting most outside noises from entering the room, thereby reducing auditory input to the brain. The high concentration Epsom salt water solution allows individuals to effortlessly float on their back while remaining completely still, reducing both proprioceptive and tactile input to the brain. The temperature of the water is calibrated to the temperature of the skin (~94° F) and the temperature of the air is calibrated to the temperature of the water, making it difficult to discern the boundary between air and water, thus reducing thermal input to the brain while minimizing the need for thermoregulation of the skin.

While both float pools dramatically reduce external sensory information, it is important to note that each floater is in full control over the experience. They can enter and exit the float pool whenever they choose. Each float pool also contains a blue LED light that can be turned on and off via an air-coupled light switch in the pool. Both float rooms contain a shower for cleaning before and after floating.

In 2016, we completed our first float-fMRI study in a sample of 40 healthy individuals, who each floated 3 times for 90 minutes per float. Half the sample was randomized to the float pool condition and the other half was randomized to the float chair control condition. In over 180 total hours of floating, there were no reported adverse events and the subjects found the overall experience to be pleasurable (see Figure 2).

Figure 2. Each bar represents a single subject's average pleasantness rating across three 90-minute float sessions using a bipolar valence scale that ranged from extremely unpleasant to extremely pleasant. Nearly every subject (n=40) reported the overall experience to be pleasurable. The only exception was a single subject in the chair condition who reported the sessions to be mildly unpleasant.

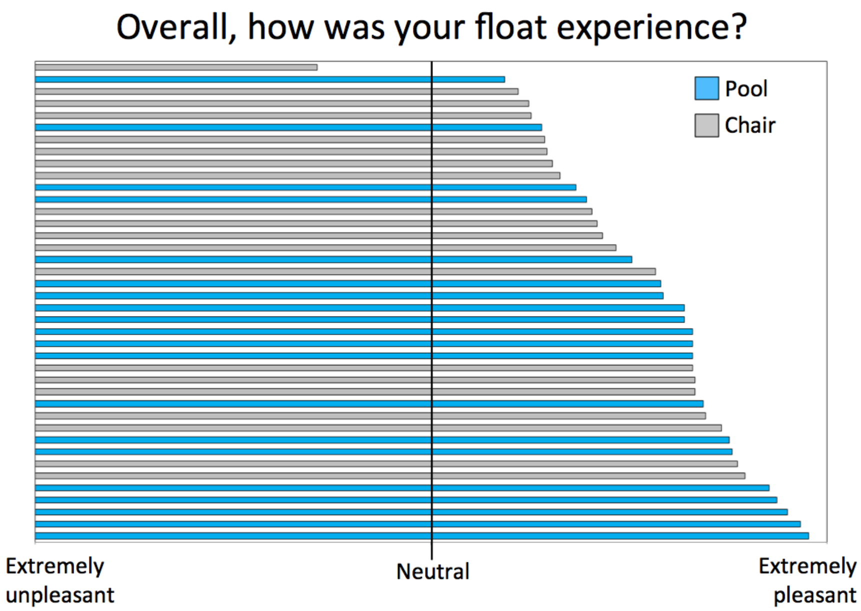

As predicted, subjects found the pool condition to be more pleasurable than the chair condition. They also reported that the pool condition induced significantly higher levels of Serenity (Figure 3) and significantly lower levels of State Anxiety (Figure 4). With regard to this latter finding, it is important to highlight the low levels of baseline anxiety reported in this healthy sample. We expect to find even larger effects with regard to post-float anxiety reduction when testing clinical populations who typically report much higher levels of baseline anxiety.

Figure 3. There was a significant (p<.001) Group (pool vs chair) x Time (pre-float vs post-float) interaction such that subjects in the pool condition reported substantially higher levels of Serenity post-float (d=1.17), a construct on the PANAS-X that measures how calm, relaxed, and at ease an individual feels. Scores were converted to POMP units representing the Percent Of Maximum Possible on the scale ranging from 0 to 100. Error bars represent the standard error of the mean.

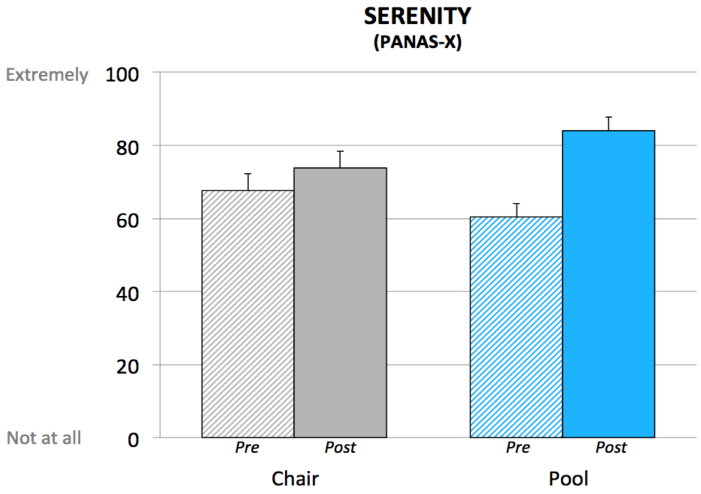

Figure 4. There was a significant ($p<.05$) Group (pool vs chair) x Time (pre-float vs post-float) interaction such that subjects in the pool condition reported lower levels of State Anxiety post-float (as measured by the Spielberger State Anxiety Inventory). Scores were converted to POMP units representing the Percent Of Maximum Possible on the scale ranging from 0 to 100. Error bars represent the standard error of the mean.

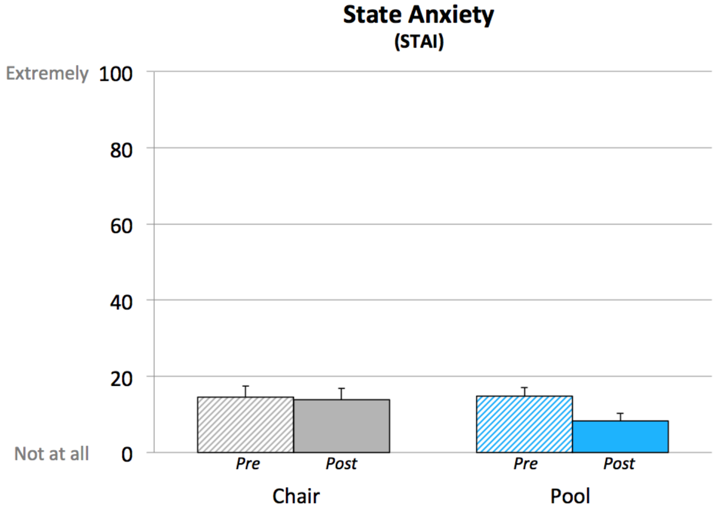

D. Research Design and Methods

Overall Study Design

We aim to recruit 300 adult participants (150 males and 150 females) between the ages of 18-80. Once a participant successfully meets the inclusion and exclusion criteria and signs the informed consent, they will be assigned into either the floatation group or control group. While we anticipate that most of the studies will use a between-subject design, once a participant has completed the study they may be offered the opportunity to switch to the other group and serve as their own control in a within-subject crossover design. Due to the paucity of previous float research examining important issues (such as the ideal length of time per float, the ideal number of float sessions, and the ideal frequency of float sessions), we have very little precedent to dictate these parameters. “Ideal” can be operationalized using a number of different measures that we plan on collecting (e.g., finding the combination of parameters that maximizes relaxation while minimizing stress/anxiety and leading to sustained gains in these domains over time). Thus, much of the preliminary work in this proposal aims to work out these details by flexibly manipulating the length of time per float (up to 150 minutes), the total number of floats (up to 30 floats), and the frequency of floats (ranging from daily to monthly). The range of these different variables was chosen to capture the typical range utilized by most clients of float centers. Importantly, all of these variables are within the range that has been documented to be safe by previous float studies (see Section B above).

As part of the core testing, in addition to floating, participants may also be asked to complete a number of different self-report, physiological, and behavioral measures (described below). For each float session, the combined total time for completing these other measures will never exceed 90 minutes, and will typically take no longer than 30 minutes.

Participants may also be given the opportunity to participate in 4 additional projects that examine:
(1) fMRI before and after floating or the control condition (henceforth referred to as the “fMRI project”),
(2) sleep starting 2 nights before floating or the control condition and continuing for up to 2 nights after

(henceforth referred to as the “Sleep project”), (3) mood starting 4 days before floating or the control condition and continuing for up to 4 days after (henceforth referred to as the “Mood project”), and (4) levels of magnesium, sulphate, cortisol, and other variables as measured in samples of blood, urine, and saliva collected before, during, and after floating (henceforth referred to as the “Bio-Samples project”). Each additional project will provide additional compensation for participation.

During the consent process, participants will be provided with their specific float protocol that will be detailed at the bottom of the informed consent. The float protocol will outline the length of time per float, the total number of floats, the frequency of floats, as well as the additional projects that they will be participating in.

Floating condition

Each float room has its own private restroom, changing area, and shower. The rooms are locked from the outside and have a red sign on the door alerting no one to knock when a float session is in progress. Prior to floating, the experimenter will give the participant a brief tour of the float facilities, teach the participant how to attach and detach the physiological equipment, and answer any questions that the participant might have. Participants will be instructed to use the restroom beforehand, disrobe in the changing area, take a shower, and then enter the float pool. Once in the pool, the participant will float comfortably in the supine position with their mouth, nose, and eyes all above the surface of the water. The participant is free to enter and exit the float pool at any time. The participant is also in control over the lights, and can turn a blue LED light on and off via an air-coupled light switch in the pool. An intercom system will allow for continuous communication between the participant and the experimenter throughout testing. At the end of the float session, participants will be cued that the float session is over with music and/or lights, at which point they will take a second shower, dry themselves and get dressed.

Control conditions

To control for the effects of simple relaxation induced by lying down in a comfortable and quiet environment, participants in the control condition will lay in the supine position in a zero-gravity chair (Human Touch PC510, Classic Power, Series 2). The material of the chair is made of leather and the cushioning is made of memory foam. The chair has an automatic control lever that the participant adjusts in order to recline the chair to the position that they find to be most comfortable. The chair is located in a quiet, temperature-controlled room, with dimly lit lighting. The zero-gravity chair will be referred to as the “floatation chair” and laying in the chair will be referred to as “floating.” All measures that are collected in the floating condition will also be collected in the control condition. With the expectation that resting in the zero-gravity chair for over an hour with no active task may be difficult for some individuals, we are adding a second control condition where participants will watch a non-arousing film while resting in the zero-gravity chair. The films will come from one of two different documentary series: BBC’s *Planet Earth* television series comprised of eleven 50-minute episodes or National Geographic’s *Cosmos: A Spacetime Odyssey* television series comprised of thirteen 44-minute episodes. Since the zero-gravity chair induces a mild state of relaxation (see figures 2 and 3), it can be considered an active control condition. In order to ascertain the full magnitude of effects related to floating we will also include two non-active control conditions (waitlist control and treatment-as-usual) comprised of participants who will complete the baseline and follow-up measures, but not undergo any float sessions in-between. Following completion, wait-list control participants will be offered the opportunity to float in the pool condition.

General Procedure

Prior to starting the study, the experimenter will go through the entire study procedure and informed consent document with the participant. Participants may be asked to complete a series of baseline self-report, physiological, and behavioral measures (see below) that will take between 1-3 hours to complete. These same baseline measures may also be completed at the end of the study and up to 1-year later. Participants who have been assigned to the floating condition must sign a Float Waiver form and will be provided with a Pre-Float Checklist.

Prior to each float session, the participant will complete a series of self-report measures (pre-measures), followed by floating, and then another set of the same self-report measures (post-measures). See below for a list of *Pre/Post Self-Report measures*. Behavioral and physiological measures (detailed below) may be collected before, during, and after floating. After each float session, participants will undergo a short debriefing where they will be given the opportunity to openly discuss their thoughts about the experience and ask any questions that they may have. The longitudinal aspect of this protocol will enable us to determine the cumulative effects of floating, especially as it pertains to the additional projects described below.

fMRI project

Participants who agree to participate in the fMRI project will have their brain scanned before and immediately after floating or the control condition. Each scan will last approximately one hour and will contain several functional tasks in addition to anatomical scans. The anatomical scans will be used to register the functional images, as well as to measure within-subject changes in white matter, cortical thickness, and brain volume over time. The maximum number of scans that a participant will undergo is 7 (3 before scans, 3 after scans, and 1 follow-up scan). The timing of the scans will typically take place at the beginning of the study, in the middle of the study, at the end of the study, and up to 1-year later.

Sleep project

Given the well-known relationship between stress and insomnia (e.g., Bonnet & Arand, 2010), as well as anecdotal reports of improved sleep after floating (e.g., Bood et al., 2007), and increased brain theta waves found during floating (Fine et al., 1993), it is reasonable to expect that floating might enhance the overall quality of sleep, particularly slow-wave sleep. Those participating in this part of the project will be sent home with a user-friendly, wireless and portable EEG system and/or a Fitbit watch which measures sleep duration using accelerometry. Participants will wear the EEG system for up to 2 nights before the float and 2 nights after the float. The Fitbit watch may be worn throughout the duration of the study.

Mood project

Very little is known about how long the effects of floating last. While anecdotal reports range from several hours to several days, this has never been empirically tested. Using the experience sampling method (Christensen et al., 2003) in combination with custom-designed software as well as Qualtrics and RedCap, we will track participant's mood during the hours and days following a float. Text messages will be individually sent to a participant's smartphone, with the message linked to a private and secure web address that is HIPAA-compliant. The texts will be sent at a rate no higher than once an hour and will begin and end at the participant's chosen wake time and sleep time. When clicked, the link will take the participant to a secure webpage where they will be asked questions about their current affective state using a subset of the questions administered in the *Pre/Post Self-Report measures*. A

human manikin will also be shown on the screen so that participants can draw the location(s) on the manikin where they feel different emotions. So for example, if they reported feeling anxious, they could draw a circle around the heart if they felt the anxiety in this location of the body. These drawings can then later be transformed into heat maps that will show the body areas where different emotional states are experienced (Nummenmaa et al., 2014).

Bio-Samples project

Blood and urine samples may be collected at different time points including baseline, before floating, immediately after floating, and two hours after floating. Follow-up samples may be collected the day after floating, as well as 1-month after the study is completed. In a single study, no participant will provide more than 16 samples of blood and urine. Collection of blood and urine will take no more than 15 minutes to collect per a set of samples. For all blood samples, a physician, R.N. or trained phlebotomist will utilize sterile techniques to draw blood by venipuncture or by the placement of a peripheral intravenous (IVP) line. Less than 100 mL of blood will be collected per subject for each blood draw, which is well within the safety limit of ~450 mL per blood draw. Up to four sets of blood samples may be obtained during each study visit.

Salivary cortisol samples may be collected in the morning, before, during, and after the float, and at nighttime in order to fully characterize each participant's circadian cortisol fluctuations. Each saliva sample should take no more than 1 minute to collect. Participants may also be asked to provide salivary cortisol samples using Salivettes™. Samples are stored and centrifuged prior to cortisol analysis. The analysis is performed using the Salimetrics Cortisol ELISA Kit (Saliva) - Assays, 1-3002 with a sensitivity of less than 0.007 micrograms per deciliter.

All samples will be deidentified (described in more detail below). Some of the samples will be sent to the Mayo Clinic laboratories in Rochester, Minnesota for processing of Magnesium levels in blood serum, erythrocytes, and urine. Additional blood samples may be collected as part of a Chem 14 mineral panel, a complete blood count, a lipid panel, as well as for measuring levels of sulphate and cortisol. Some of these samples will require 12 hours of fasting, in which case we will collect them in the morning. These additional samples, as well as the salivary cortisol, will be processed by a laboratory at St. Francis Hospital or by Dr. William Potter in his laboratory at the University of Tulsa. De-identified blood and saliva samples for genetic and epigenetic banking may be collected and stored (for later processing and analysis) in secure -80°C freezers located at LIBR or Dr. Kent Teague's laboratory at the University of Oklahoma.

Baseline Self-Report measures (not all of these measures will be collected during every study)

<u>Anxiety Sensitive Index (ASI-3 and ASI-R):</u> These instruments include items designed to measure the fear of arousal-related sensations, specifically along the dimensions/subscales of Physical, Cognitive, and Social Concerns. Each item is answered on a scale of 0-4 ("very little" to "very much"). The scales have been found to have adequate performance on several measures of reliability and validity (Deacon & Abramowitz, 2006; Taylor et al., 2007).

<u>Drug Abuse Screening Test (DAST-10)</u>: The DAST-10 (Skinner, 1982) is a brief version of the 28-item DAST designed to identify drug-use related problems in the previous year. It has demonstrated good internal consistency and temporal stability in psychiatric samples; the DAST-10 discriminates between psychiatric outpatient with or without drug use disorders (using scores between 2-4; Cocco & Carey, 1998). This measure consists of 10 yes/no questions. Responding yes to score greater than 2 of the

questions is considered an indicator that the individual should seek further evaluation for problematic drug use behaviors.

Fordyce Happiness Measure (HM): The Happiness Measure is a tool used to measure emotional wellbeing by providing evidence of a person's perceived happiness (Fordyce, 1988). The HM consists of three questions on happiness to assess how happy/unhappy the individual feels. The first question asks the person to choose a statement that best describes their average happiness on a scale from "0" (Extremely unhappy) to "5" (Neutral) to "10" (Extremely happy). The second question asks the individual to rate the percentage of time they feel "happy," "unhappy" and "neutral" with percentages totally up to 100%. The third and final question asks for the person to rate how they feel at the current moment on a linear sliding scale from -5 (Bad), 0 (Neutral) and 5 (Good). The HM has been reviewed in relation to general well-being measurements and has been proven to be a quick and efficient measurement tool with good reliability and discriminative validity (Fordyce, 1988).

Mini-International Neuropsychiatric Interview (MINI) version 6: A validated, structured, diagnostic interview with questions that parallel symptoms in the Diagnostic and Statistical Manual of Mental Disorders (DSM-IV-TR). The MINI was administered by interviewers trained in the assessment of anxiety and depression, and all psychiatric diagnoses were confirmed via an extensive review of the clinical history by a board-certified psychiatrist.

Overall Anxiety Severity and Impairment Scale (OASIS): The OASIS is a brief questionnaire (5 Items) that can be used as a continuous measure of anxiety-related severity and impairment across anxiety disorders. Each item is rated on a 5-point scale and the ratings are summed to obtain a total score. A cut-score of 8 has been shown to correctly classify 87% of individuals as having an anxiety diagnosis or not (Campbell-Sills et al., 2009). The OASIS has demonstrated excellent 1-month test–retest reliability, and convergent and divergent validity (Norman et al., 2006).

Patient Health Questionnaire (PHQ-9): The Patient Health Questionnaire (PHQ) is a self-administered diagnostic instrument for common mental disorders. The PHQ-9 is the depression module, which scores each of the 9 DSM-IV criteria as "0" (not at all) to "3" (nearly every day). Scores of 1-4 are considered minimal depression, 5-9 mild depression, 10-14 moderate depression, 15-19 moderately severe depression and 20-27 severe depression (Kroenke, Spitzer & Williams, 2001).

Perceived Stress Scale (PSS): The Perceived Stress Scale is a psychological instrument used for measuring an individual's perception of stress. It has become one of the most widely used instruments for measuring the degree to which situations in one's life are appraised as stressful. The PSS has adequate internal reliability and proves it is a valid method for assessing perceived stress in a person's life (Fliege et al., 2005). The PSS is suggested for examining the role appraised stress has in the etiology of disease and is a good measuring tool for levels of experienced stress (Cohen, Kamarck & Mermelstein, 1983).

Sheehan Disability Scale (SDS): The SDS assesses how much the respondent's mental health issues are perceived to have affected their daily activities in three functional domains: work/school, social/leisure activities, and family life/home responsibilities. Total disability scores range between 0 to 30, with scores ≥ 5 signifying impairment. A review of studies using this measure indicated significant impairment in functioning in patients with anxiety disorders, who have mean total disability scores typically ranging between 14-18.

State-Trait Anxiety Inventory (STAI-Trait form): This is a widely-used psychometric instrument designed to assess an individual's anxiety proneness. This measure has both a "state" subscale meant to measure temporary anxiety symptoms and a "trait" subscale meant to measure more long-standing anxiety

proneness. Each subscale consists of 20 items using 4-point scales ("not at all" to "almost always"). The STAI is a validated measure with good internal consistencies for both subscales and has high test-retest reliability for the trait subscale and low to moderate test-retest reliability for the state measure (Spielberger, 2010).

Pre/Post Self-Report measures (not all of these measures will be collected during every study)

Automated Self-Administered 24-hour Dietary Recall (ASA24): This is a freely available Web-based tool (http://appliedresearch.cancer.gov/asa24/) developed by the National Cancer Institute that enables automated self-administered 24-hour recalls of dietary consumption of food and computes precise amounts of nutrient intake (including amount of magnesium) based on the participant's responses. Since diet is an important factor that can affect magnesium levels in the blood and urine, participants who are enrolled in the Bio-Sample portion of the study will need to complete this measure that has recently been validated against the gold-standard (Thompson et al., *in press*).

Debriefing Interview: A list of questions that may be asked to the participant after each float.

Effects Checklist: Participants rate the degree to which they experienced a variety of positive and negative effects during the float experience and/or control condition.

Floating Scales: This set of scales was specifically created by our group to examine the effects of floating as they relate to an individual's impression of the experience. The scales cover a range of questions tapping into emotion, mood, time perception, and sensory processing.

Karolinska Sleepiness Scale (KSS): The KSS is a single item measure of present moment sleepiness that has been validated against relevant behavioral and electroencephalography measures.

Positive and Negative Affect Schedule - Expanded Form (PANAS-X): The PANAS is a widely used measure comprising 20-items assessing general states of positive and negative affect using 5-point scales (1 = very slightly/not at all, 5 = extremely). The expanded form contains 60 items that measure 11 specific affects: Fear, Sadness, Guilt, Hostility, Shyness, Fatigue, Surprise, Joviality, Self-Assurance, Attentiveness, and Serenity. To assess momentary affect, participants will rate how they feel "right now at the present moment". The PANAS has high internal consistency, good convergent, discriminant, and construct validity, and is one of the most commonly used mood measures (Watson et al., 1988).

State-Trait Anxiety Inventory (STAI-State form): This is a widely-used psychometric instrument designed to assess an individual's anxiety proneness. This measure has both a "state" subscale meant to measure temporary anxiety symptoms and a "trait" subscale meant to measure more long-standing anxiety proneness. Each subscale consists of 20 items using 4-point scales ("not at all" to "almost always"). The STAI is a validated measure with good internal consistencies for both subscales and has high test-retest reliability for the trait subscale and low to moderate test-retest reliability for the state measure (Spielberger, 2010).

Wong-Baker Pain scale: This commonly used pain measure has participants rate their current level of pain from 0 to 10 using drawings of faces that range from smiling to crying.

Physiological measurement during floating:

Before, during, and after the floatation and control conditions, as well as during some of the baseline testing, subjects may be being wearing several different recording devices in order to measure heart

rate, respiration, blood pressure, EEG, and movement (of note, tegaderm may used with the BioPatch and NeuroVerse Brainstation in order to create a waterproof barrier).

EEG System: For tracking changes in electrical potential on the scalp, subjects will be wearing the Neuroverse Brainstation (Neuroverse Inc., 4660 La Jolla Village Drive, Suite 740, San Diego, CA 92122). The Neuroverse Brainstation is a wireless, portable EEG device placed on the subject's forehead, and additionally covered with Tegaderm adhesive film prior to the start of the recording session. A mobile app (BrainVitals) implemented in iPhones and IPads will be used to control both stimuli presentation and EEG data recording. The Neuroverse EEG system is comprised of an EEG recording circuit board connected to ultrathin, flexible electronics sensors. The epidermal electronic system (EES) sensors are heterogeneous, ultra-thin film structures consistimg of FDA-approved, bio-compatible polymer backing materials or 3M Tegaderm™ and stretchable circuits made of biologically inert materials (silicon and gold). The EES sensors are adequate for recording electrophysiological signals (ECG/EMG/EEG). In addition to the superior "liquid proofing" granted by this system, advantages of the Neuroverse EEG system include subject comfort, rapid application and clean up, with no need for conductance gel, and importantly wireless remote control with high quality signal-to-noise (SNR) EEG in real-time. For its application, it adheres to the surface of the skin by soft contact, much like a typical sticker adhesive. A novel mobile iOS application (BrainVitals) developed for iPad and iPhone, will be used to provide recording control and stimulus presentation in the pre and post-float periods.

Zephyr BioPatch: For wireless real-time measurement of heart rate, heart rate variability, respiration rate, skin temperature, posture, and accelerometry, subjects will be wearing the Zephyr BioPatch (Zephyr Technology, 1 Annapolis St, Suite 200, Annapolis, MD 21401). This system consists of a small physiological monitoring module that connects wirelessly to a computer and is attached anywhere on the torso using two standard ECG electrodes. The device is a CE-certified, FDA-approved Class II device and is water resistant up to 1m (IP67). It is further protected from water using Tegaderm. The BioPatch is powered by a 3.7 V rechargeable lithium ion battery.

Blood Pressure System: For measuring blood pressure, participants will be wearing the QardioArm Blood Pressure Monitor (Qardio, Inc., 115 Sansome St, Suite 888, San Francisco, CA 94104). These systems consists of a module connected to a cloth band that is snugly wrapped around the upper arm and connects wirelessly to a computer, iPhone, or iPad via Bluetooth. The Qardioarm is powered by four 1.5-volt AAA alkaline batteries, and can be wirelessly triggered to collect blood pressure via an Apple iPad or iPhone. Once triggered, the cloth band constricts for approximately 30 seconds and then releases. The device has been validated for measuring upper arm blood pressure according to the European Society of Hypertension International Protocol (ESH-IP) revision 2010 (Shang et al., 2013). Participants will wear the device on their left upper arm underneath a reusable waterproof protector (LimbO waterproof protector, Thesis Technology Products Ltd, Brooks Green Farm, Brooks Lane, Bosham, Chichetser, West Sussex PO18 8JX, United Kingdom).

Movement detection: In order to objectively examine the number of movements and the percentage time still during each float session, we use a Microsoft Kinect infrared camera, as well as accelerometry devices attached to the Equivital system or worn as a band around the wrist. The Kinect uses a technique called structured light to obtain depth data. A grid of infrared light is shined onto a scene, and then the distortion of the grid is used to calculate each pixel's distance from the Kinect. The Kinect has an internal algorithm that decomposes this depth data into 20 skeletal joints when a human is in view, and these joints are used to track movement. The Kinect has three streams of data that can be enabled: the color stream, the depth stream, and the skeletal stream. Our program can only enable and access the skeletal stream, meaning that only the coordinates of the skeletal joints are available for

users to use and view. This protects the privacy of each participant by ensuring that color and depth images are always unavailable. As an added form of protection, the RGB color camera of the Kinect is covered with black electrical tape to entirely block the float pool and the participant from its view. Our program will never use this camera, so the tape is just an added precaution.

Squeeze Ball: The custom-built Squeeze Ball is a wireless hand dynamometer that uses a 5-volt rechargeable Lithium Phosphate battery to measure the pressure change in a handheld squeeze bulb and then wirelessly transmits the data from a Radio Frequency Module enclosed in a waterproof plastic housing that seals the battery and internal circuitry from the water. Participants will use this Squeeze Ball during the float to communicate subjective ratings using a continuous scale (e.g., to report how much stress they feel from not at all to extremely).

E. Statistical Methods:

Data will be analyzed using standard univariate and multivariate statistical parametric methods except for instances that require the use of non-parametric statistical analysis. Examples of these methods include analysis of variance (ANOVA), t-tests, multi-dimensional scaling, correlation analyses, clustering algorithms and functional connectivity. Both within- and between-subject effects will be explored, and whenever possible, a repeated measures mixed-model ANOVA design will be utilized to examine group by time interactions. MRI data will be analyzed using standard functional and anatomical analysis methods. In the case of anatomical images, we may utilize automated programs for parcellating brain regions, measuring cortical thickness and curvature, and specialized algorithms for specifying white matter track direction. Analysis of functional MRI data will follow the basic procedure of 1) image registration both to other functional images and the associated anatomical images, 2) conversion to standardized brain space, 3) intensity standardization followed by 4) individual inferential statistical tests such as multiple regression using convolved task time series as predictor variables. Group analyses of functional data will include both parametric and non-parametric statistical methods in standard image space in order to determine the reliability of within-group effects as well as to determine the presence of between-group differences. During data processing, analysts will remain blinded to group membership whenever possible. Between-group power analyses of fMRI data have shown that acceptable effect sizes can be obtained with 16 to 20 subjects per group. These effect sizes are significantly influenced both by voxel size, type of task, and the brain region of interest. Calculation of sample sizes will follow the statistical power analysis calculation published by Desmond and Glover (2002).

F. Gender/Minority/Pediatric Inclusion for Research

Women and minorities will be included in the study without prejudice according to their representation in the study population. Adult subjects will be recruited from the greater Metro Tulsa area and should thus share the racial and ethnic composition of this area. All efforts will be made to ensure that our participant population closely resembles the gender, ethnic and racial composition of the greater Tulsa area. Children are not included in this protocol as the studies are focused on the adult human brain.

G. Human Participants

We anticipate testing up to 300 adult participants, approximately half male and half female, with an age range between 18 and 80 years of age, who will be recruited through LIBR's screening protocol (WIRB # 20101611) or LIBR's T-1000 protocol (WIRB # 20142082). Two-thirds of the participants (n=200) will be healthy and free of any current or past neurological or psychiatric illness, and one-third of the participants (n=100) will have a psychiatric history characterized by high levels of anxiety. The inclusion

and exclusion criteria will be determined via LIBR's screening protocol (WIRB #20101611) or via LIBR's T-1000 protocol (WIRB # 20142082), unless otherwise noted below.

Table 1. Inclusion and Exclusion criteria

Inclusion criteria	Exclusion Criteria
1. DSM-IV-TR diagnosis on the MINI of an Anxiety Disorder (Generalized Anxiety Disorder, Social Anxiety Disorder, Panic Disorder, Agoraphobia) and/or Posttraumatic Stress Disorder (PTSD)<br>2. Overall Anxiety Severity and Impairment Scale (OASIS) score ≥ 8<br>3. Anxiety Sensitivity Index (ASI-3) total score ≥ 30<br>4. If taking medication, must be stably medicated prior to participation (defined as having taken the medication for 6 weeks or longer)<br>5. Between 18-55 years of age<br>6. No prior Floatation-REST experience	1. Comorbid Bipolar Disorder or Schizophrenia<br>2. Active suicidality with intent or plan<br>3. Currently receiving inpatient treatment<br>4. Current Substance Use Disorder ≥ moderate<br>5. History of neurological conditions (e.g., epilepsy, stroke, severe traumatic brain injury, Parkinson's disease, Alzheimer's disease or other forms of dementia)<br>6. Any skin conditions or open wounds that could cause pain when exposed to saltwater<br>7. Inability to swim or lay comfortably in a shallow pool of water

Inclusion criteria:

All participants must be able to provide written informed consent and must have sufficient proficiency in the English language to understand and complete interviews, questionnaires, and all other study procedures.

Participants in the healthy sample must be free of any current or past neurological or psychiatric illness.

Participants in the psychiatric sample must have an Overall Anxiety Severity and Impairment Scale (OASIS) score ≥ 8 and a DSM diagnosis of an anxiety disorder as determined by the Mini International Neuropsychiatric Interview (MINI Version 6.0). Comorbid conditions are acceptable (except those listed in the exclusion criteria). Participants from the psychiatric sample will either be outpatients who are stably medicated prior to participation (defined as having taken the medication for 6 weeks or longer) or individuals who are not currently receiving any treatment.

Exclusion criteria:

(1) Has any of the following DSM-V disorders:
    a. Schizophrenia Spectrum and Other Psychotic Disorders
    b. Bipolar and Related Disorders
(2) Is currently being treated for their psychiatric condition as an inpatient.
(3) Active suicidal ideation with intent or plan.
(4) Participant is morbidly obese (BMI > 40) or underweight (BMI < 17).

(5) Certain drugs or medications consumed within the past week including any psychoactive drugs (e.g., MDMA, LSD, psilocybin, peyote, phencyclidine, ketamine), magnesium supplements (greater than 150mg) or milk of magnesia. Any antihistamine that causes drowsiness (e.g., Benadryl) or any alcohol consumed within the past 12 hours. Caffeine or nicotine consumed within the past 3 hours. For all other medications, we require the participant to be stably medicated prior to participation (defined as having taken the medication for 6 weeks or longer).
(6) Participant has a history of unstable liver or renal insufficiency; glaucoma; diabetes; significant and unstable cardiac, vascular, pulmonary, gastrointestinal, endocrine, neurologic, hematologic, rheumatologic, or metabolic disturbance; or any other condition that, in the opinion of the investigator, would make participation not be in the best interest (e.g., compromise the well-being) of the subject or that could prevent, limit, or confound the protocol-specified assessments.
(7) Any sign of hypermagnesemia as detected in serum magnesium levels that are greater than 8 mg/dL or by the loss of the patellar reflex.
(8) Any evidence of kidney damage or disease detected in the complete blood panel. Kidney status can be calculated by using serum creatinine, age, gender, and ethnicity in the MDRD equation to estimate the glomerular filtration rate (GFR) and thus renal function. A GFR < 90 mL/min indicates mild Kidney Damage (Levey et al., 1999).
(9) Pregnancy as detected by a urine test.
(10) Non-correctable vision or hearing problems.
(11) Unwillingness or inability to complete any of the major aspects of the study protocol. However, failing to complete some individual aspects will be acceptable (e.g., being unwilling to answer individual items on a questionnaire).
(12) MRI contraindications including: cardiac pacemaker, metal fragments in eyes/skin/body (shrapnel), aortic/aneurysm clips, prosthesis, by-pass surgery/coronary artery clips, hearing aid, heart valve replacement, shunt (ventricular or spinal), electrodes, metal plates/pins/screws/wires, or neuro/bio-stimulators (TENS unit), persons who have ever been a professional metal worker/welder, history of eye surgery/eyes washed out because of metal, vision problems uncorrectable with lenses, inability to lie still on one's back for 60-120 minutes; prior neurosurgery; tattoos or cosmetic makeup with metal dyes, unwillingness to remove body piercings, and pregnancy.

Plans for recruitment and consent procedures to be followed:

Recruitment of participants will occur through LIBR's assessment team, which maintains a repository of healthy and anxious participants who have been assessed and screened through other approved Western IRB protocols (WIRB #20101611 and #20142082). We will also recruit through publicly displayed flyers and internet advertisements and through word of mouth via clinicians in the community. All participants recruited through these latter methods must first be consented and screened through LIBR's screening protocol (WIRB # 20101611) prior to commencing this study. Participants who meet our inclusion/exclusion criteria will be offered the opportunity to participate.

Consenting will be conducted in private exam rooms at the Laureate Institute for Brain Research. Consent will be obtained by members of the research team that have received training on the consenting process for this study. Written informed consent will be obtained from each participant after they have been provided a full verbal and written explanation of the study purpose, procedures, risks and benefits, and after they have been allowed sufficient opportunity to review this information and ask questions concerning any aspect of the study.

MRI screening procedures

For MRI studies, each participant will complete an MR safety-screening questionnaire immediately upon completion of the consent form. The MRI screening questionnaire is developed and distributed by the Institute for Magnetic Resonance Safety, Education, and Research (IMRSER) in Los Angeles, CA[1]. IMRSER is a non-profit organization sponsored by major MRI-related corporations, including GE (the manufacturer of LIBR's 3T magnet), to "disseminate information regarding current and emerging MR safety issues" and "to develop and provide materials and resources to facilitate MR safety-related education and training". The safety-screening questionnaire probes for possible occupational exposure to metal slivers or shavings (remnants of which may remain lodged in a subject's head or neck), surgical clips or shrapnel, cochlear implants, or any other form of ferrous metal implanted in or on the participant's body. Participants answering in the affirmative to any of these conditions will be excluded. All participants with any form of implanted wires, metal, or electronic devices will be excluded. Although there are no known risks of MR to pregnant women, there may be unknown risks. Therefore, females who are pregnant must not participate in this protocol.

H. Risks

Risks associated with self-report questionnaires:

The risks associated with completing the self-report questionnaires are minimal. Some of the questions may be uncomfortable to answer and long questionnaires may elicit boredom.

Risks associated with the physiological measures:

Most of the devices are commercially available, safe, and non-invasive, posing minimal risk to the participant. Electrodes from the EEG and EKG may leave a small indent on the participant's body that will dissipate once the device is removed. Male participants may need to shave their chest hair in order to attach the EKG electrodes. The electrode gel might leave a sticky residue that can be easily washed off with warm water. Wearing the sleep profiler EEG system may cause a minor headache related to the pressure of the system on the forehead.

All of the batteries in the physiological equipment are low voltage. If any device's battery is exposed to water, the electric circuit will be shorted. In the rare event that the device somehow passes a current beyond the battery, the current will follow the path of least resistance and dissipate throughout the salt water before passing through the surface of the skin. This means that participants will likely NOT feel an electric shock or be exposed to any electrical danger even when the battery comes into contact with the water. Salt water is very conductive compared with the human body, and much of the electric current is shunted around the outside of the body. Although the leads of the batteries will never be touching the participant directly, we can estimate a worst-case scenario by assuming that the skin resistance is at least 3 kΩ in water (Fish & Geddes, 2009). Based on the low voltage batteries being used in the physiological equipment, under a worst-case scenario the current for an individual piece of equipment should never exceed 2 mA. At this low level, the current is barely perceptible to most humans (Dalziel,

---

[1] INSTITUTE FOR MAGNETIC RESONANCE SAFETY, EDUCATION, AND RESEARCH 7511 McConnell Avenue, Suite 100, Los Angeles, CA 90045

http://www.imrser.org

1972), and at least 3 times lower than the amount of current necessary to induce a feeling of pain (*cf.* Occupational Safety & Health Administration: https://www.osha.gov/Publications/osha3075.pdf).

Risks associated with MRI:

MRI uses powerful magnetic fields and weak radio frequency pulses (electromagnetic radiation), neither of which has been associated with adverse effects in patients or laboratory animals when studied under clinical imaging protocols. MRI centers across the country are regularly using up to 4 Tesla MRI scanners for research purposes. However, there are the following sources of risk: (1) the participant may experience physical discomfort being in the scanner, (2) some participants may feel anxious or claustrophobic in the confined space of the MRI scanner, (3) the strong magnetic field will affect electronic, magnetic, and metal devices that participants carry with them or that have been implanted in the participant's body, (4) occasionally some participants may experience muscle twitching or paresthesias, especially in the torso, due to peripheral nerve stimulation effects of the MRI scanner, (5) some individuals may experience light-headedness or dizziness while in the scanner or when rising from the MRI gurney too rapidly, and (6) MRI scanners produce a loud high frequency tone that can cause hearing damage if appropriate hearing protection is not used.

Risks associated with blood draws:

The procedure for providing a blood sample is the same as that used for routine blood tests. Insertion of the needle may be painful for a brief time. The level of risk is considered to be minimal. The risks of venipuncture are minimal. Possible mild side effects include bruising at the site of the venipuncture. The risks for placement of an IVP include local bleeding, possible infection, or local inflammation of the skin and/or vein with pain and swelling. During all blood draws, a trained phlebotomist, physician, or nurse will utilize sterile techniques.

Blood samples may be stored for future genetic research. All blood samples will be labeled with a de-identified number (i.e., a number assigned to a participant which links to identifying information stored securely at LIBR). De-identified blood samples will be stored in the lab of Dr. Kent Teague at the University of Oklahoma College of Medicine, and de-identified samples that have been processed will be

Risks associated with floating:

Minor risks that have been associated with floating include: slipping and falling due to the wet environment, feeling disoriented or scared due to the dark, occasional neck discomfort while floating, visual or auditory hallucinations in a small subset of participants, minor ear ache related to the salt crystals drying in the ear canal, stinging of wounds or cuts from the salt water, burning in the eyes or a bitter taste in the mouth if directly exposed to the salt water, mild skin rash due to the salt, feeling uncomfortable being naked while floating, and general boredom associated with the experience. Major risks associated with floating are extremely rare. To date, there have only been 2 reported deaths, both of which involved individuals who consumed large quantities of sedative drugs prior to floating (Lann & Martin, 2010). In both cases, the coroner related the primary cause of death to drug toxicity. There have been no reported incidents of drowning, and the buoyancy provided by such high concentrations of Epsom salts make the possibility of drowning extremely improbable. Due to the paucity of research exploring magnesium absorption via Epsom Salt water, it is presently unknown whether floating can induce a state of hypermagnesemia. To date, hypermagnesemia has never been reported in the literature, including in individuals who floated for more than 30 floats (Bood et al., 2007), the maximum number allowed in our study.

Procedures for protecting against or minimizing potential risks:

Throughout testing, the researcher will repeatedly remind the participant that participation is strictly voluntary and they have the right to withdraw at any time without penalty.

In order to minimize risks associated with the self-report measures, we will inform the participant that they may decline to answer any specific questions or discontinue the interview at any time.

In order to minimize risks associated with the blood draw, the trained phlebotomist will always utilize sterile techniques to draw blood.

Procedures to minimize MRI associated risk:

In order to minimize risks associated with MRI, each participant will complete a safety-screening questionnaire prior to entering the MR environment. The safety pre-screening questionnaire probes subjects for possible occupational exposure to metal slivers or shavings remnants of which may remain lodged in the subject's head or neck. Subjects with surgical clips or shrapnel, cochlear implants, or any other form of ferrous metal body implanted in or on their body will be excluded. All subjects with any form of implant wires, metal or electronic device implants will be excluded. All persons involved in this protocol will receive MR safety training conducted at the Laureate Institute for Brain Research by their MR safety officer.

We intend to minimize claustrophobic problems using a series of procedures: 1) by giving a detailed explanation of the environment prior to scanning, 2) maintaining voice contact with the subjects at all times, 3) maintaining visual contact of the participant in the scanner using observational cameras placed inside the scanner room. Participants will also be provided with the opportunity to lay in a mock scanner prior to the actual MRI.

To minimize the risk of hearing damage, all participants will be fitted with ear plugs and be required to wear the hearing protection for the duration of the MRI scanning.

For women: A pregnancy test will be obtained from women of child-bearing potential prior to the fMRI scan. Women who are pregnant will be excluded from the study. A urine pregnancy test will be performed immediately prior to any MRI scanning to determine eligibility.

Procedures to minimize risks associated with floating:

Throughout testing, the researcher will repeatedly remind the participant that they are in complete control over the experience and can always exit the pool. We are mitigating the risks associated with floating in the following ways:

(1) In order to minimize the chances of slipping and falling, both float rooms contain a waterproof slip-resistant floor (Altro Marine 20: wet slip rating = 0.77). Grab bars are situated at the entrance and exit of each float pool. The participant will be explicitly instructed to enter and exit each float pool carefully and to make use of the grab bars.

(2) Feelings of disorientation and fear of the dark will be minimized by giving participants complete control over the lights. They can turn the lights on and off by pressing an air-coupled light switch in the pool anytime during the float.

(3) To minimize the effects of hallucinations it will be emphasized to the participant that they are free to leave the float pool or turn on the lights whenever they feel uncomfortable. It will also be emphasized that it is entirely natural for the brain to occasionally create visual or auditory patterns in this unique environment.

(4) To minimize the chances of an ear ache related to the salt crystals drying in the ear canal, participants will be provided with silicone waterproof earplugs that they can wear during the float. Each shower will also contain a squirt bottle containing a mixture of distilled white vinegar (which helps disintegrate the salt crystals) and isopropyl alcohol (which helps to dry any moisture), and participants will be instructed to pour this mixture into their ears after each float.

(5) Stinging of wounds or cuts from the salt water will be minimized by providing participants with a vaseline-based ointment that they are instructed to place on the wound or cut prior to floating.

(6) Stinging of eyes or the bitter taste of salt in the mouth will be minimized by forewarning all participants to be careful not to splash water in their face while floating. In the event that the salt water should get in their eyes or mouth, a squirt bottle of fresh water is located at the edge of each float pool and can be used to wash away the salt water.

(7) In order to minimize any skin rash associated with the exfoliating nature of the dry Epsom salt, all participants will shower after the float and will be provided with skin lotion for moisturizing after the shower.

(8) To minimize feelings of discomfort associated with floating naked, the door to the float room/restroom will always be locked and a sign will be placed on the door to ensure no one accidentally enters. Any time the participant interacts with the experimenter after they disrobe, they will always be wearing a hospital gown to ensure adequate privacy.

(9) Feelings of boredom during the float experience may arise and participants will be instructed that they can end the float session at anytime if such feelings become too uncomfortable.

(10) To minimize the chances of someone floating while intoxicated, we will explicitly instruct all participants to refrain from using any drugs and alcohol before or during the float experience. Participants who arrive intoxicated will immediately be excluded from the study.

(11) To minimize the chance of hypermagnesemia being developed over the course of the study, the patellar reflex will be assessed by the experimenter after every fifth float session. Any participant who is found to lack the reflex will undergo a blood draw to check serum Magnesium levels and be excluded if their levels are greater than 8 mg/dL. No further float sessions will be conducted prior to receiving the results from the serum Magnesium test.

(12) To minimize the chance of a participant experiencing an unforeseen adverse event while floating, an experimenter will always be nearby in an adjacent control room. While in the control room, the investigator will be listening to a continuous real-time audio feed from a high-powered microphone placed in the float room. Thus, the experimenter can swiftly act in the event of an emergency. If a participant reports experiencing any negative or adverse events, they will be given the opportunity to speak directly with a licensed psychiatrist or clinical psychologist.

Potential benefits and importance to the participants and others:

Participants may experience reduced levels of stress and anxiety, in addition to increased levels of relaxation during and after their float experience. The results of these studies will further the scientific community's understanding of floating and its effects on the central and autonomic nervous system. Future research may discover important clinical applications of floating for helping individuals with neurological or psychiatric illness. As such, participants may derive personal satisfaction from their contribution to the discovery process.

Plan of Action for Incidental Findings:

Upon detection of incidental findings an investigator will communicate the discovery to the participant verbally and/or by written communication. The participant will be encouraged to contact their primary-care physician about the discovery. LIBR will provide a digital copy of the suspect MR scans and/or physiological data to the primary-care physician upon request once the subject provides written consent authorizing the release of these medical records. Additionally, detection and disclosure of incidental findings will be documented in a database contained on the Laureate Institute for Brain Research computer cluster.

I. Confidentiality

To protect participant confidentiality, blood and data samples will be anonymized as follows:

1. Last name: All participants will be assigned the last name "LIBR."
2. First name: The first name will be a secure alpha cryptographic hash based on LIBR participant ID. This technique is the gold standard in computer security for one-way correlation of data.

Records of the participant's participation in this study will be held confidential, except when disclosure is required by law, or as described in the informed consent document (under "Confidentiality"). The study doctor, the sponsor or persons working on behalf of the sponsor, and under certain circumstances, the United States Food and Drug Administration (FDA) and the Institutional Review Board (IRB) will be able to inspect and copy confidential study-related records that identify the subject by name. Therefore, absolute confidentiality cannot be guaranteed. If the results of this study are published or presented at meetings, the subject will not be identified.

Subjects in MR studies will have both anatomical and functional MRI scans. Paper copies of consents, screening forms, the Research Privacy Form, and any other forms, testing results or papers containing Personally Identifiable Information (PII) will be stored in a secured room with access granted only to authorized personnel.

There is risk of possible loss of participant confidentiality. To minimize risk, all study records that identify the participant (including contact information such as phone number and email address) will be kept in a locked record room and only study personnel will have the entry code. The digital videos from each participant's post-float debriefing will only be used for the purposes of this research protocol unless the participant provides their written consent to use these films for additional purposes. The videos will always be stored on password-protected computers and de-identified, which means that there is no name, address, birthdate or other identifiable information linked to them. All hard copy data will be stored in a locked cabinet. Any study records entered into a computer system or on electronic media

will be assigned code numbers and will not be individually identifiable. Code numbers are a combination of numbers and letters.

J. Data and Safety Monitoring Plan

The risk for adverse events is minimal. There will be no external monitoring. A physician will be available for consultation for any medical or safety issues. Any adverse events, unanticipated adverse events, and/or reportable deviations will be reported immediately to the Laureate Institute for Brain Research Human Protection Administrator at (918) 502-5155 or via email at hpa@laureateinstitute.org. Any adverse events including unanticipated adverse events, and/or reportable deviations will be reported to the Western IRB as required. Additionally, due to the relative novelty of this line of work, we will remain vigilant throughout the study. An investigator will remain in the control room any time a participant is floating. While in the control room, the investigator will be listening to a continuous real-time audio feed from a high-powered microphone placed in the float room. Thus, the investigator can swiftly act in the event of an emergency. Any Serious Adverse Event (SAE) will be immediately reported to the LIBR Scientific Director (Dr. Martin Paulus) and a SAE form from the NIH Common Data Element will be completed. An SAE is defined as any untoward medical occurrence that: results in death, is life-threatening, requires inpatient hospitalization or prolongation of existing hospitalization, results in persistent or significant disability/incapacity, or is a congenital anomaly/birth defect. While we don't anticipant any SAE's occurring, we will always be prepared in case of emergency with first aid kits, fire extinguishers, and medical staff onsite.

The protocol, informed consent document, and relevant supporting information must be submitted to the IRB for review and must be approved before the study is initiated. In addition, any participant recruitment materials must be approved by the IRB prior to use. This study will be conducted in accordance with the ethical principles that have their origin in the Declaration of Helsinki and that are consistent with Good Clinical Practice and applicable regulatory requirements. The study must be conducted in accordance with the regulations of the United States Food and Drug Administration (FDA) as described in 21 CFR 50 and 56, applicable laws and the IRB requirements.

The sponsor must submit any change to the protocol to the IRB for review and approval before implementation. A protocol change intended to eliminate an apparent immediate hazard to participants may be implemented immediately, provided the reviewing IRB is notified within 5 working days.

It is the responsibility of the investigator to provide each subject with full and adequate verbal and written information, before inclusion in the study, using the IRB approved informed consent document, including the objective and procedures of the study and the possible risks involved. Informed consent must be obtained prior to performing any study-related procedures. A copy of the signed informed consent document must be given to the study subject.

K. Literature Cited:

Asmundson, G. J., & Stein, M. B. (1994). Triggering the false suffocation alarm in panic disorder patients by using a voluntary breath-holding procedure. *The American Journal of Psychiatry*, *151*(2), 264-266

Barabasz, A., Barabasz, M., Dyer, R., & Rather, N. (1993). Effects of chamber REST, flotation REST and relaxation on transient mood state. In *Clinical and Experimental Restricted Environmental Stimulation* (pp. 113-120). Springer New York.

Bonnet, M. H., & Arand, D. L. (2010). Hyperarousal and insomnia: state of the science. *Sleep medicine reviews*, *14*(1), 9-15.

Bood, S. Å., Sundequist, U., Kjellgren, A., Norlander, T., Nordström, L., Nordenström, K., & Nordström, G. (2006). Eliciting the relaxation response with the help of flotation-rest (restricted environmental stimulation technique) in patients with stress-related ailments. *International Journal of Stress Management*, *13*(2), 154-175.

Bood, S. Å., Sundequist, U., Kjellgren, A., Nordström, G., & Norlander, T. (2007). Effects of flotation rest (restricted environmental stimulation technique) on stress related muscle pain: are 33 flotation sessions more effective than 12 sessions?. *Social Behavior and Personality: an international journal*, *35*(2), 143-156.

Campbell-Sills, L., et al., *Validation of a brief measure of anxiety-related severity and impairment: the Overall Anxiety Severity and Impairment Scale (OASIS).* J Affect Disord, 2009. *112*(1-3), 92-101.

Christensen, T.C., Barrett, L.F., Bliss-Moreau, E., Lebo, K., and Kaschub, C. (2003). A practical guide to experience-sampling procedures. J. Happiness Stud. 4, 53–78.

Cocco, K.M. and Carey K.B. (1998). Psychometric properties of the Drug Abuse Screening Test in psychiatric outpatients. Psychological Assessment, *10*(4), 408-414.

Cohen, S., Kamarck, T., & Mermelstein, R. (1983). A global measure of perceived stress. *Journal of health and social behavior*, 385-396.

Crandall, R. A. (1986). The use of ultraviolet light in the treatment of water in public spas and hot tubs. *Journal of Environmental Health*, *49*(1), 16-23.

Dalziel, C. F. (1972). Electric shock hazard. *Spectrum, IEEE*, *9*(2), 41-50.

Deacon, B., & Abramowitz, J. (2006). Anxiety sensitivity and its dimensions across the anxiety disorders. *Journal of Anxiety Disorders*, *20*(7), 837-857.

Del Re, A. C., Fluckiger, C., Goldberg, S. B., & Hoyt, W. T. (2013). Monitoring mindfulness practice quality: an important consideration in mindfulness practice. *Psychother Res, 23*(1), 54-66.

Dierendonck, D., & Te Nijenhuis, J. (2005). Flotation restricted environmental stimulation therapy (REST) as a stress-management tool: A meta-analysis. *Psychology & Health*, *20*(3), 405-412.

Fine, T. H., & Turner, J. W. (1982). The effect of brief restricted environmental stimulation therapy in the treatment of essential hypertension. *Behaviour Research and Therapy*, *20*(6), 567-570.

Fine, T., Mills, D., & Turner Jr, J. (1993). Differential effects of wet and dry flotation REST on EEG frequency and amplitude. In *Clinical and Experimental Restricted Environmental Stimulation* (pp. 205-213). Springer New York.

Fish, R. M., & Geddes, L. A. (2009). Conduction of electrical current to and through the human body: a review. *Eplasty*, *9,* 407-421.

Fliege, H., Rose, M., Arck, P., Walter, O. B., Kocalevent, R. D., Weber, C., & Klapp, B. F. (2005). The Perceived Stress Questionnaire (PSQ) reconsidered: validation and reference values from different clinical and healthy adult samples. *Psychosomatic medicine*, *67*(1), 78-88.

Fordyce, M. W. (1988). A review of research on the happiness measures: A sixty second index of happiness and mental health. *Social Indicators Research*, *20*(4), 355-381

Forgays, D. G., & Belinson, M. J. (1986). Is flotation isolation a relaxing environment?. *Journal of Environmental Psychology*, *6*(1), 19-34.

Friedman, L., Stern, H., Brown, G. G., Mathalon, D. H., Turner, J., Glover, G. H., ... & Potkin, S. G. (2008). Test–retest and between-site reliability in a multicenter fMRI study. *Human brain mapping*, *29*(8), 958-972.

Jacobs, G. D., Heilbronner, R. L., & Stanley, J. M. (1984). The effects of short term flotation REST on relaxation: a controlled study. *Health Psychology*, *3*(2), 99-112.

John, O.P. and S. Srivastava, *The Big-Five trait taxonomy: History, measurement, and theoretical perspectives.*, in *Handbook of Personality: Theory and Research*, L.A. Pervin and O.P. John, Editors. 1999, Guilford Press: New York. p. 102-138.

Kjellgren, A., Sundequist, U., Norlander, T., & Archer, T. (2001). Effects of flotation-REST on muscle tension pain. *Pain Research & Management 6*(4), 181-189.

Koula, G. M., Kemp, J. C., Keane, K. M., & Belden, A. D. (1990). Replication of a clinical outcome study on a hospital-based stress management and behavioral medicine program utilizing flotation REST and biofeedback. In *Restricted Environmental Stimulation* (pp. 202-209). Springer, New York.

Kroenke, K., R.L. Spitzer, and J.B. Williams, *The PHQ-9: validity of a brief depression severity measure.* JGen Intern Med, 2001. *16*(9), 606-13.

Levey AS, Bosch JP, Lewis Jb, Grenne T, Rogers N, Roth D. (1999). A more accurate method to estimate glomerular filtration rate from serum creatinine: a new prediction equation. Modification of diet in renal disease study group. *Ann Intern Med,* 130, 461-470.

Lilly, John C. and Jay T. Shurley. 1961. "Experiments in Solitude in Maximum Achievable Physical Isolation with Water Suspension of Intact Healthy Persons" in Psychophysiological Aspects of Space Flight. Columbia University Press, New York. 238-247.

Lilly, J. C. (1977). *The deep self: Profound relaxation and the tank isolation technique*. Simon & Schuster.

Norlander, T., Kjellgren, A., & Archer, T. (2001). The experience of flotation-REST as a function of setting and previous experience of altered state of consciousness. *Imagination Cognition and Personality*, *20*(2), 161-178.

Norman, S.B., et al., *Development and validation of an Overall Anxiety Severity And Impairment Scale (OASIS).* Depress Anxiety, 2006. *23*(4), 245-9.

Nummenmaa, L., Glerean, E., Hari, R., & Hietanen, J. K. (2014). Bodily maps of emotions. *Proceedings of the National Academy of Sciences*, *111*(2), 646-651.

O'Leary, D. S., & Heilbronner, R. L. (1990). Flotation REST and information processing: A reaction time study. In *Restricted Environmental Stimulation* (pp. 113-124). Springer New York.

Paulus, M. P., Feinstein, J. S., Castillo, G., Simmons, A. N., & Stein, M. B. (2005). Dose dependent decrease of activation in bilateral amygdala and insula by lorazepam during emotion processing. Archives of General Psychiatry, 62, 282-288.

Pudvah, M. B., & Rzewnicki, R. (1990). Six months in the tank: Long-term effects of flotation isolation on state anxiety, hostility, and depression. *Restricted Environmental Stimulation: Theoretical and empirical developments in flotation REST*, 169-173.

Schulz, P., & Kaspar, C. H. (1994). Neuroendocrine and psychological effects of restricted environmental stimulation technique in a flotation tank. *Biological Psychology*, *37*(2), 161-175.

Sheehan, D.V., et al., *The Mini-International Neuropsychiatric Interview (M.I.N.I.): The development and validation of a structured diagnostic psychiatric interview for DSM-IV and ICD-10.* Journal of Clinical Psychiatry, 1998. *59,* 22-33.

Shurley, J. T. (1960). Profound experimental sensory isolation. *American Journal of Psychiatry*, *117*(6), 539-545.

Shurley, J. T. (1966). Stress and adaptation as related to sensory/perceptual isolation research. *Military medicine*, *131*(3), 254-258.

Spielberger, C. D. (2010). *State-Trait Anxiety Inventory*. John Wiley & Sons, Inc.

Stein, M. B., Simmons, A., Feinstein, J. S., & Paulus, M. P. (2007). Increased amygdala and insula activation during emotion processing in anxiety-prone subjects. The American Journal of Psychiatry, 164, 318-327.

Suedfeld, P., & Coren, S. (1989). Perceptual isolation, sensory deprivation, and rest: Moving introductory psychology texts out of the 1950s. *Canadian Psychology*, *30*(1), 17.

Suedfeld, P., Ballard, E. J., & Murphy, M. (1983). Water immersion and flotation: From stress experiment to stress treatment. *Journal of Environmental Psychology*, *3*(2), 147-155.

Suedfeld, P., & Eich, E. (1995). Autobiographical memory and affect under conditions of reduced environmental stimulation. *Journal of Environmental Psychology*, *15*(4), 321-326.

Taylor, S., Zvolensky, M. J., Cox, B. J., Deacon, B., Heimberg, R. G., Ledley, D. R., ... & Cardenas, S. J. (2007). Robust dimensions of anxiety sensitivity: development and initial validation of the Anxiety Sensitivity Index-3. *Psychological assessment*, *19*(2), 176.

Thompson FE, Dixit-Joshi S, Potischman N, Dodd KW, Kirkpatrick SI, Kushi LH, Alexander GL, Coleman LA, Zimmerman TP, Sundaram ME, Clancy HA, Groesbeck M, Douglass D, George SM, Schap TE, Subar AF. Comparison of interviewer-administered and automated self-administered 24-hour dietary recalls (ASA24) in three diverse integrated health systems. *Am J Epidemiol*, in press.

Turner Jr, J. W., & Fine, T. H. (1983). Effects of relaxation associated with brief restricted environmental stimulation therapy (REST) on plasma cortisol, ACTH, and LH. *Biofeedback and Self-regulation*, *8*(1), 115-126.

Turner, J. W., & Fine, T. H. (1991). Restricting environmental stimulation influences levels and variability of plasma cortisol. *Journal of Applied Physiology*, *70*(5), 2010-2013.

Turner Jr, J., Gerard, W., Hyland, J., Nieland, P., & Fine, T. (1993). Effects of wet and dry flotation REST on blood pressure and plasma cortisol. In *Clinical and Experimental Restricted Environmental Stimulation* (pp. 239-247). Springer New York.

Zubek, J. P. (1969). *Sensory deprivation: Fifteen years of research*. Appleton-Century-Crofts and Fleschner Publishing Company.
